# Insight into Genotype-Phenotype Associations through eQTL Mapping in Multiple Cell Types in Health and Immune-Mediated Disease

**DOI:** 10.1371/journal.pgen.1005908

**Published:** 2016-03-25

**Authors:** James E. Peters, Paul A. Lyons, James C. Lee, Arianne C. Richard, Mary D. Fortune, Paul J. Newcombe, Sylvia Richardson, Kenneth G. C. Smith

**Affiliations:** 1 Department of Medicine, University of Cambridge School of Clinical Medicine, Cambridge, United Kingdom; 2 Cambridge Institute For Medical Research, University of Cambridge, Cambridge, United Kingdom; 3 MRC Biostatistics Unit, Cambridge Institute of Public Health, Cambridge, United Kingdom; 4 Immunoregulation Section, Autoimmunity Branch, National Institute of Arthritis and Musculoskeletal and Skin Diseases, Bethesda, United States of America; 5 JDRF/Wellcome Trust Diabetes and Inflammation Laboratory, Department of Medical Genetics, University of Cambridge, Cambridge, United Kingdom; University College London, UNITED KINGDOM

## Abstract

Genome-wide association studies (GWAS) have transformed our understanding of the genetics of complex traits such as autoimmune diseases, but how risk variants contribute to pathogenesis remains largely unknown. Identifying genetic variants that affect gene expression (expression quantitative trait loci, or eQTLs) is crucial to addressing this. eQTLs vary between tissues and following *in vitro* cellular activation, but have not been examined in the context of human inflammatory diseases. We performed eQTL mapping in five primary immune cell types from patients with active inflammatory bowel disease (n = 91), anti-neutrophil cytoplasmic antibody-associated vasculitis (n = 46) and healthy controls (n = 43), revealing eQTLs present only in the context of active inflammatory disease. Moreover, we show that following treatment a proportion of these eQTLs disappear. Through joint analysis of expression data from multiple cell types, we reveal that previous estimates of eQTL immune cell-type specificity are likely to have been exaggerated. Finally, by analysing gene expression data from multiple cell types, we find eQTLs not previously identified by database mining at 34 inflammatory bowel disease-associated loci. In summary, this parallel eQTL analysis in multiple leucocyte subsets from patients with active disease provides new insights into the genetic basis of immune-mediated diseases.

## Author Summary

The human immune system has evolved to protect us from infection and cancer, whilst avoiding damage to healthy tissue. If this complex system goes wrong, immune cells may cause inappropriate inflammation and damage, resulting in clinical disease. Examples include inflammatory bowel disease and autoimmune vasculitis, characterised by inflammation in the gut and blood vessels respectively. Genetic studies have identified many variants in our DNA code that predispose to such immune-mediated diseases. The majority of these variants lie outside protein-coding regions, and so how they influence disease risk remains largely unclear. Examining how genetic variants affect gene expression can help bridge this gap in our knowledge, but these effects are highly dependent on the cellular or environmental context such as tissue type or cellular activation status. We investigated the genetic control of gene expression in five white blood cell subtypes taken from patients with active inflammatory bowel disease and autoimmune vasculitis, and from healthy controls. We report the novel observation of distinct variants that only affect gene expression in patients with active inflammatory disease, and show that these effects can disappear following treatment. These findings provide new insights into the genetic basis of important immune-mediated diseases.

## Introduction

Most of the hundreds of disease-associated single nucleotide polymorphisms (SNPs) identified by genome-wide association studies (GWAS) lie outside protein-coding regions, and are presumed to act by regulating gene expression [1, 2]. Investigating the effects of allelic variation on transcription by expression quantitative trait locus (eQTL) mapping provides insights into how risk loci influence disease susceptibility, and may identify pathways amenable to pharmacological intervention. eQTLs vary considerably between tissues and cell types [3–5], and so when attempting to interpret GWAS signals through eQTL data, the context in which eQTLs are present is critically important. eQTLs have been previously examined in cell lines [6, 7] or in one or two primary immune cell types [5, 8, 9], but a comparative analysis of eQTLs across a broad range of the major leucocyte subsets implicated in immune-mediated disease pathogenesis has not yet been carried out.

Most autoimmune diseases exhibit less than 50% concordance in monozygotic twins, highlighting the importance of environmental factors in their pathogenesis [10]. Studies in model organisms show that eQTLs vary in different environmental conditions [11, 12], and *in vitro* stimulation of primary human immune cells can both abrogate and induce eQTLs [13–15]. These experiments cannot, however, reproduce the *in vivo* inflammation that characterizes human autoimmune and inflammatory disease. Moreover, these studies have typically been limited to a few hundred genes (e.g. ref.s [14, 15]).

We hypothesised that an analysis of gene expression across different immune cell types in both health and active inflammatory disease could provide additional insight into associations between genotype and phenotype. Our study across five immune cell types provides the most comprehensive comparison to date of the cells known to play roles in immune-mediated disease, and includes neutrophils, a key immune cell type for which a systematic eQTL analysis has not been reported. We examined eQTLs across approximately 8,000 genes selected in an unbiased manner, and by including both patients with active inflammatory disease and healthy controls, we reveal eQTLs present only in the context of human *in vivo* inflammation. We anticipate that such eQTLs may be important in understanding the heterogeneity in immune responses between individuals, and may have implications for understanding the inter-individual variation in clinical course seen in autoimmune and infectious diseases.

## Results

We performed eQTL mapping using CD4 T cells, CD8 T cells, B cells, monocytes, and neutrophils isolated from the peripheral blood of healthy volunteers (HVs) and patients with rigorously defined evidence of active inflammatory bowel disease (IBD: ulcerative colitis or Crohn’s disease) or anti-neutrophil cytoplasmic antibody (ANCA)-associated vasculitis (AAV). Blood was taken from IBD and AAV patients prior to initiation of treatment of the disease flare. We performed transcriptome profiling using Affymetrix HuGene ST1.1 gene expression microarrays. IBD patients and HVs were genotyped using the Illumina Human OmniExpress12v1.0 BeadChip, and AAV patients using the Affymetrix SNP 6.0 platform.

### Multiple immune cell-type eQTL analysis

Limiting the initial analysis to 91 IBD patients and 43 HVs genotyped on the Illumina Human OmniExpress12v1.0 BeadChip (see Table 1 for sample sizes by cell type), we mapped local-acting (*cis*) eQTLs using an additive genetic model for 9,041 probesets corresponding to 7,552 unique Entrez Gene IDs selected by highest variance (see Methods). Using separate analysis of each cell type, we found a *cis* eQTL in at least one cell type for 4,524 (59.9%) of the 7,552 genes analysed (5% FDR, see S1 Data for summary statistics).

**Table 1:** Sample sizes.

	CD4 T cell	CD8 T cell	Monocyte	Neutrophil	B cell	All	All*	N
Healthy	42	41	41	39	20	18	35	43
IBD	79	67	83	82	60	47	58	91
AAV	41	40	45	43	-	-	33	46

Number of individuals with expression and genotype data available following quality control, by disease and cell type. Column ‘All’ indicates the numbers of individuals for whom expression data were available for all cell types.

*Samples available for all cell types excluding B cells. Column ‘N’ indicates the total number of individuals in each cohort.

We next investigated sharing of eQTLs across cell types. Previous reports have claimed that eQTLs are highly specific to different leucocyte subsets [5]. These conclusions were based on separate eQTL analysis in each cell type, followed by comparison of the resulting lists of significant hits in each. As Flutre *et al* [16] highlighted, this approach fails to account for incomplete power and leads to exaggerated claims of cell-type specificity. An eQTL truly present in two cell types may pass the significance threshold in one but may fail to meet significance in another, and thus falsely be called a cell-type specific eQTL. This may be due to random noise, or because there is insufficient power to detect an eQTL which is weaker but nonetheless present in the second cell type.

**Fig 1:**
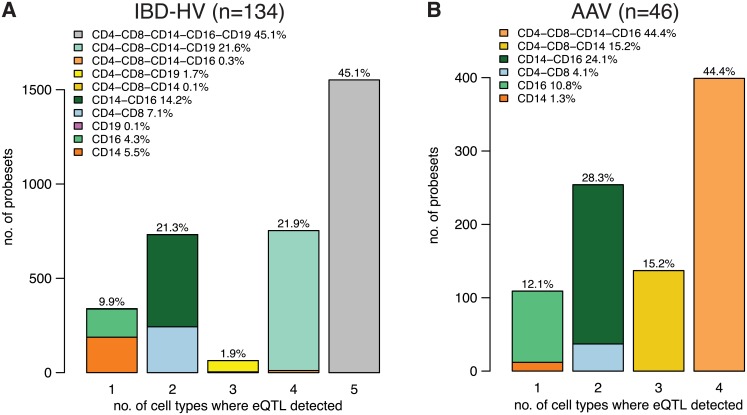
Estimates of eQTL sharing across leucocyte subsets from joint modelling of expression data across cell types (eQTLBMA). Number of probesets with an eQTL, subsetted according to the number of cell types in which the eQTL was declared present. Each bar is subdivided according to which cell type the eQTL was detected in. The denominator for the percentages shown is the total number of probesets for which an eQTL was detected in at least one cell type. Analysis using PEER-adjusted expression data. (A) IBD and HV analysis. (B) AAV analysis. B cell RNA was not available for a sufficient number of AAV patients for analysis. Key: CD4 = CD4 T cells, CD8 = CD8 T cells, CD14 = monocytes, CD16 = neutrophils, CD19 = B cells.

In order to more accurately estimate the degree of sharing of eQTLs across immune cell subsets, we performed joint analysis across the 5 cell types using a Bayesian model (eQTL Bayesian Model Averaging, or ‘eQTLBMA’ [16]) that, for each SNP-gene pair, assesses the evidence for each possible configuration of eQTL absence/presence across the 5 cell types. There are thus 2^5^ − 1 = 31 possible configurations for an eQTL to be present in at least one of 5 tissues. Through joint analysis of all 5 cell types, we found that 3,440 probesets (corresponding to 3,185 unique Entrez Gene IDs) had a significant *cis* eQTL (5% Bayes FDR). For each of these probesets, we took the SNP with the highest posterior probability of being the eQTL. Then for the resulting list of SNP-probeset pairs, we compared the posterior probabilities for all possible configurations of eQTL presence/absence across cell types. This approach revealed that 45.1% of eQTLs have the highest posterior probability for presence across all 5 cell types, whereas only 9.9% have the highest posterior probability for presence of the eQTL in just one cell type (Fig 1A). In stark contrast, using the naïve approach of separate analysis of each cell type, followed by comparison of the lists of significant eQTLs found in each, over half of eQTLs identified (5% FDR) were detected in only one cell type (S1 and S2 Figs and see S1 Text). This finding suggests that previous reports of marked immune cell-type eQTL specificity from separate tissue analysis [5] were overstated.

For the AAV patients (n = 46), expression data were not available for B cells, so we used eQTLBMA to perform eQTL modelling jointly across 4 cell types. This analysis detected eQTLs for 899 probesets (corresponding to 845 genes), using a 5% Bayes FDR. Consistent with the analysis of the IBD-HV data, 44.4% of eQTLs had the best posterior probability for presence across all 4 cell types, and only 12.1% were specific to a single cell type (Fig 1B). However, there was a greater proportion of probesets with neutrophil-specific eQTLs in the AAV analysis (10.8%, versus 4.3% in the IBD-HV data), perhaps reflecting the increased neutrophil turnover or activation that is prominent in AAV. This observation did not simply reflect that fewer cell types were examined in the AAV analysis; re-analysis of the IBD-HV data excluding B cells found that 4.8% were neutrophil-specific (S1 Text, S3 Fig). The finding of a higher proportion of neutrophil-specific eQTLs was also apparent from the one-at-a-time cell-type analysis; in AAV 27.2% of eQTLs were detected in neutrophils only versus 9.7% in the IBD-HV data (S1 Fig).

**Fig 2:**
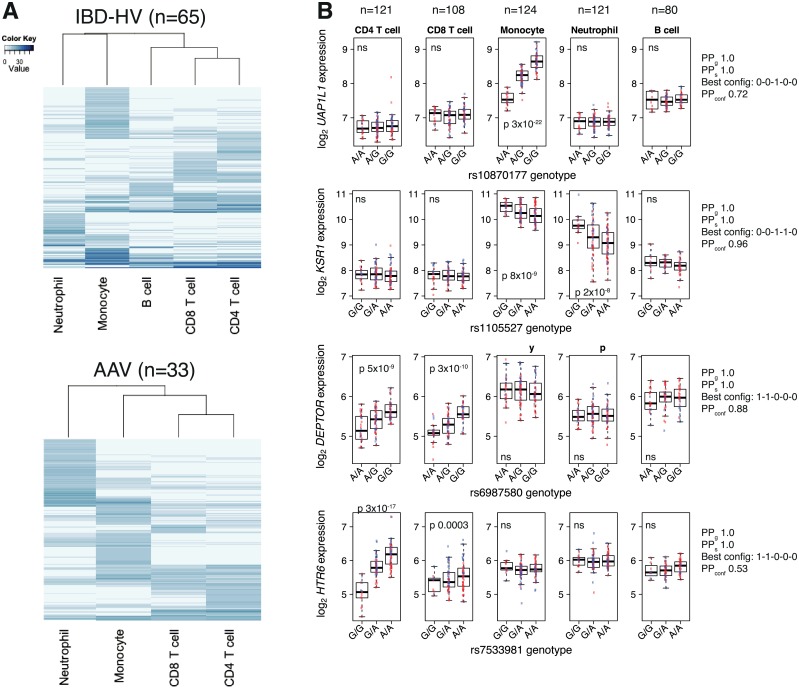
eQTL sharing is related to haematopoietic lineage. (A) Heatmap representation of the matrix of association statistics (chi-squared scores) from one-at-a-time cell-type *cis* eQTL scans using PEER-adjusted expression data. Rows are SNP-gene pairs, columns are cell types, and the association statistic (chi-squared score) is represented by the shading in the colour key. The stronger the association the darker the blue shading. Rows and columns have both been hierarchically clustered. Analysis has been limited to the set of individuals with complete expression data across cell types to ensure equal statistical power (5 and 4 cell types in the IBD-HV and AAV analyses respectively). (B) Examples of cell- and lineage-specific eQTLs. From top: monocyte-specific, myeloid-specific, and two T lymphocyte-specific eQTLs. For ease of biological interpretation, here we show RMA-normalised expression values before adjustment for batch, sex and latent factors with PEER. Boxplots indicate median, and upper and lower quartiles. For PEER-adjusted values, see S4 Fig. Blue and red dots represent HV and IBD respectively. P-values are from association using PEER-adjusted data. ns = not significant. PP_g_ = posterior probability that the gene has at least one eQTL in at least one cell type (joint cell type analysis with eQTLBMA). PP_s_ = posterior probability that the SNP is an eQTL for the gene in at least one cell type, given that the gene has an eQTL. ‘Best config’ = best configuration of eQTL presence (1) / absence (0) across the 5 cell types. PP_conf_ = posterior probability for the best configuration.

Hierarchical clustering of cell types by their eQTL test statistics from separate cell-type analyses recapitulated their haematopoietic differentiation; the myeloid and lymphoid cells cluster together, with CD4 and CD8 T lymphocytes most closely related (Fig 2A). Examples of this sharing of eQTLs between related cell types include a myeloid-specific eQTL for *KSR1* (at a GWAS locus for Crohn’s disease) and T cell-specific eQTLs for *DEPTOR* and *HTR6* (Fig 2B, S4 Fig). We quantified the similarity in eQTL profiles between cell types using the Jaccard coefficient. The Jaccard coefficient is defined as the intersect divided by the union of two sets (here, the number of eQTLs common to a pair of cell types, as a proportion of the total number of eQTLs identified in either or both cell types). Using the Bayesian method of joint analysis across cell types, the Jaccard coefficient for CD4 and CD8 T cells was 100% for both the IBD-HV dataset and the AAV dataset (S5 Fig). Sharing of eQTLs between lymphoid and myeloid cells was less common, with, for example, Jaccard coefficients of 48% and 45% between CD4 T cells and neutrophils in the IBD-HV and AAV data respectively.

For comparison, we also calculated the Jaccard coefficients based on eQTLs detected through separate analysis of each cell type. To do this, we restricted the analysis to individuals for whom expression data were available for all cell types being compared (n = 65 for the 5 celltypes in the IBD-HV analysis, and n = 33 for the 4 cell types in the AAV analysis). By using the same set of individuals, the sample size and genotype matrix (predictor variables) were identical for each cell type. This both ensures equal statistical power for each cell type and controls for inter-individual variability. Nevertheless, as highlighted by Flutre *et al* [16], separate tissue analysis results in under-estimation of eQTL sharing as a consequence of incomplete power (as occurs with any finite sample size), even if power is equal for each tissue. Using separate cell-type analysis, CD4 and CD8 T cells again had the most similar eQTL profiles, but the Jaccard coefficient was 40% in the IBD-HV analysis, and only 31% in AAV (S1 Fig). The lower proportion in the AAV dataset highlights how, as sample size and power decrease, the under-estimation of eQTL sharing that results from separate cell-type eQTL analysis worsens. This confirms that separate cell-type analysis identifies fewer shared eQTLs than joint analysis across cell types with the Bayesian method.

To investigate whether the Bayesian model unduly favoured declaring eQTLs as shared across all cell types, we randomly permuted the sample labels for the CD4 T cell expression data, leaving the genotype data and the expression data for the other cell types unchanged, and then re-ran the analysis with eQTLBMA. After permutation of the CD4 T cell expression data, very few eQTLs were declared in CD4 T cells, and the Jaccard coefficient between CD4 and CD8 T cells was 0.1% (S1 Text, S6 Fig), indicating that eQTLBMA is behaving appropriately.

To explore the possibility that eQTL cell-type specificity reflected lack of gene expression in some cell types, we re-ran the joint eQTL mapping across cell types in the IBD-HV data, limiting analysis to 5,186 probesets with evidence of robust expression in all 5 cell types (Methods). This showed that eQTL sharing was greater amongst these genes, with 64% of eQTLs found in all 5 cell types (S7 Fig). Therefore, lack of expression accounts for some, but not all, of the eQTL cell-type specificity that we observed.

**Fig 3:**
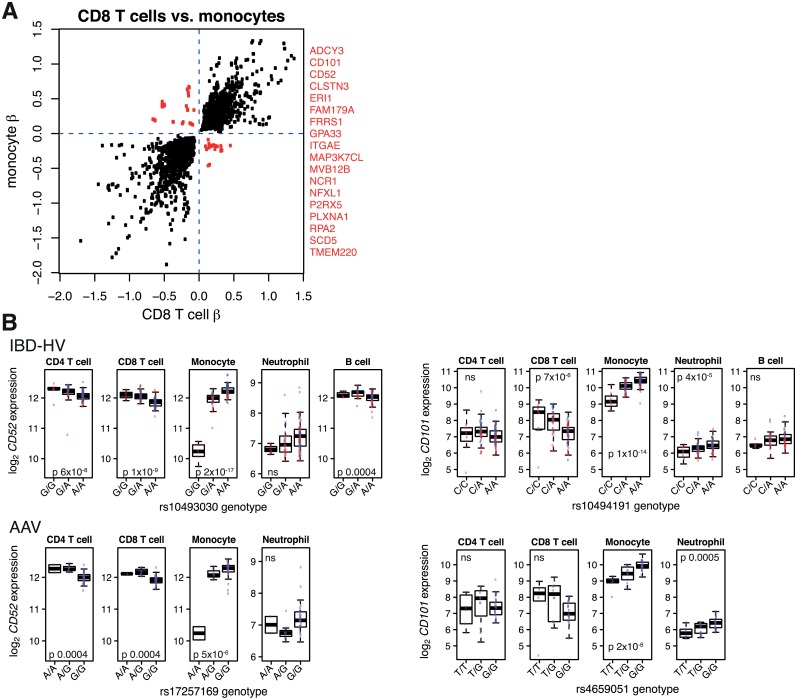
eQTLs with opposing directions of effect between cell types. (A) Detection of eQTLs with opposing directions of effect between CD8 T cells and monocytes (IBD-HV data). Each point represents a SNP-gene association which was significant in both cell types. The axes show the estimated effect size (beta) of genotype on expression in each cell type. SNP-gene pairs with opposing directions of effect between cell types, and the names of these genes, are shown in red. (B) Two examples of such divergent eQTLs. Upper panels: IBD-HV data genotyped on Illumina Human OmniExpress Beadchip (red points = IBD patients, blue = HVs). Lower panels: this observation is replicated in AAV cohort genotyped on Affymetrix SNP 6.0 platform. *r*^2^ 1 between rs10493030 and rs17257169. *r*^2^ 0.6 between rs10494191 and rs4659051.

Finally, whilst most eQTLs shared across cell types had the same direction of effect, a small proportion of these “shared” eQTLs acted in opposing directions (Fig 3A, S8–S11 Figs). Examples include eQTLs for *CD52* and *CD101* (Fig 3B). Independent analysis of the AAV cohort confirmed this observation, indicating that these eQTLs with discordant effects between cell types are unlikely to be false positives (S1 Text).

### Identification of eQTLs specific for active inflammatory disease

To identify eQTLs that are specific for active inflammatory disease in a statistically robust manner, we analysed the IBD-HV data using a linear model with a genotype × disease interaction (G×D) term. A significant G×D interaction term for a SNP-gene pair indicates that the effect of genotype on expression is significantly different in IBD versus health. In biological terms, this includes (i) eQTLs present in health but abrogated in IBD, (ii) eQTLs present in IBD but not in health, (iii) eQTLs with opposing directions of effect in health compared to IBD, and (iv) eQTLs whose effects in health and IBD are in the same direction, but of significantly different magnitudes. More formally, the interaction term assesses whether there is a significant difference in the slope of the genotype-expression regression line between healthy individuals and IBD patients i.e. whether the effect size of a unit change in allele dose on expression is significantly different between health and disease (S12 Fig). Fitting a model with an interaction term is statistically more robust than the naïve approach of separate analysis of IBD and HV cohorts, followed by comparison of the resulting lists to find eQTLs specific to one group or the other (see Methods and S1 Text).

**Fig 4:**
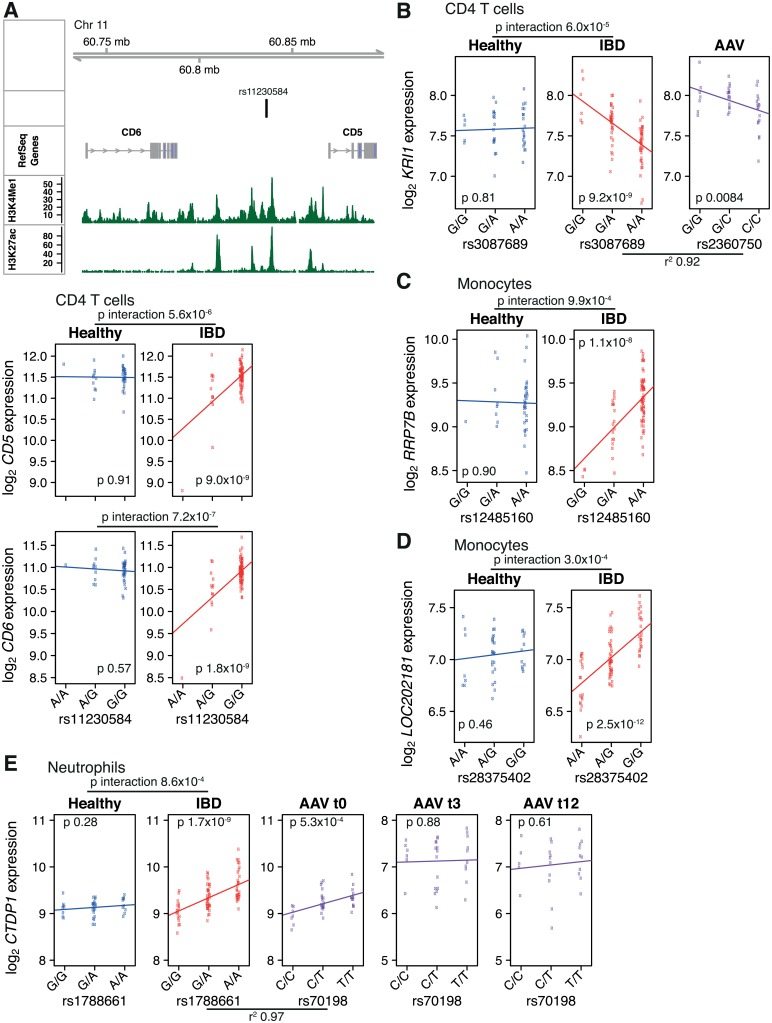
Distinct eQTLs in active inflammatory disease. Analysis using expression data batch-corrected with ComBat. (A) An eQTL for *CD5* and *CD6* is present in CD4 T cells from patients with active IBD but not in HVs. Upper panel: genomic features around the eQTL SNP including nearby genes and histone marks from ENCODE lymphoblastoid cell line (hg19 build). Lower panels: expression versus genotype boxplots. The lines indicate the estimated best fit line from the regression of expression on genotype within each cohort. The p-value for the test of the null hypothesis that the coefficient for the genotype term equals zero is shown on the plot. The p-value for genotype × disease interaction term is shown above. (B-D) Further examples of inflammatory disease-dependent eQTLs. Where a proxy SNP was present on the Affymetrix SNP 6.0 array, the AAV expression data is also shown. (E) Inflammation-specific eQTL. t0 = 0 months, t3 = 3 months, t12 = 12 months.

We used a ‘two-step’ procedure to increase power (Methods) and a 15% FDR as the significance threshold. Given the relative lack of statistical power to detect interaction effects compared to main effects, and our relatively modest sample sizes, we felt a 15% FDR provided a reasonable balance between false positives and false negatives. Using this threshold, we identified 13 genes with an eQTL exhibiting a G×D interaction effect across the cell types examined (Fig 4, S1 Table). In CD4 T cells, for example, rs11230584, a SNP located between *CD5* and *CD6* was associated with expression of both genes in IBD patients but not in healthy individuals (Fig 4A). For *CD6*, uncorrected p-values, Benjamini-Hochberg adjusted p-values and q-values for the G×D interaction were 7.2×10^−7^, 0.00048, and 0.00048 respectively, and for *CD5* 5.6×10^−5^, 0.0038, and 0.0019. CD5 and CD6 play important roles in lymphocyte signalling (see Discussion), and therefore this inflammatory disease-specific eQTL is likely to lead to differences in immune responses according to genotype. Interestingly, not all the genes with disease-specific eQTLs have strictly immunological functions. For example, in neutrophils we identified an eQTL for *CTDP1* in both the IBD and the AAV cohorts that was absent in health (Fig 4E). *CTDP1* encodes a protein phosphatase, FCP1, which regulates gene expression by dephosphorylating the C-terminus of the largest subunit of RNA polymerase II. Thus this analysis reveals the novel and potentially clinically relevant observation of eQTLs present only in the context of inflammatory disease.

For patients with AAV, in addition to the baseline samples, we also measured gene expression in peripheral blood monocytes and neutrophils taken 3 months and 12 months into treatment (Table 2). The treatment of active AAV begins with induction therapy with intensive immunosuppression. This consists of high-dose corticosteroids, and cyclophosphamide (a cytotoxic agent) or rituximab (a monoclonal antibody against CD20 which causes B cell depletion). The corticosteroid dose is slowly weaned, and once a period of stable remission has been achieved, patients are switched to maintenance immunosuppression (typically low-dose corticosteroids plus azathioprine). In the example of *CTDP1*, we found that the eQTL present in active IBD and AAV is absent in AAV patients at 3 or 12 months, providing convincing evidence that the eQTL is inflammation- rather than disease-specific.

**Table 2:** AAV pre- and post-treatment sample sizes.

	time 0	3 months	12 months
Monocyte	45	29	24
Neutrophil	43	31	26

Number of AAV patients with expression and genotype data available following quality control, by cell type and time point.

**Fig 5:**
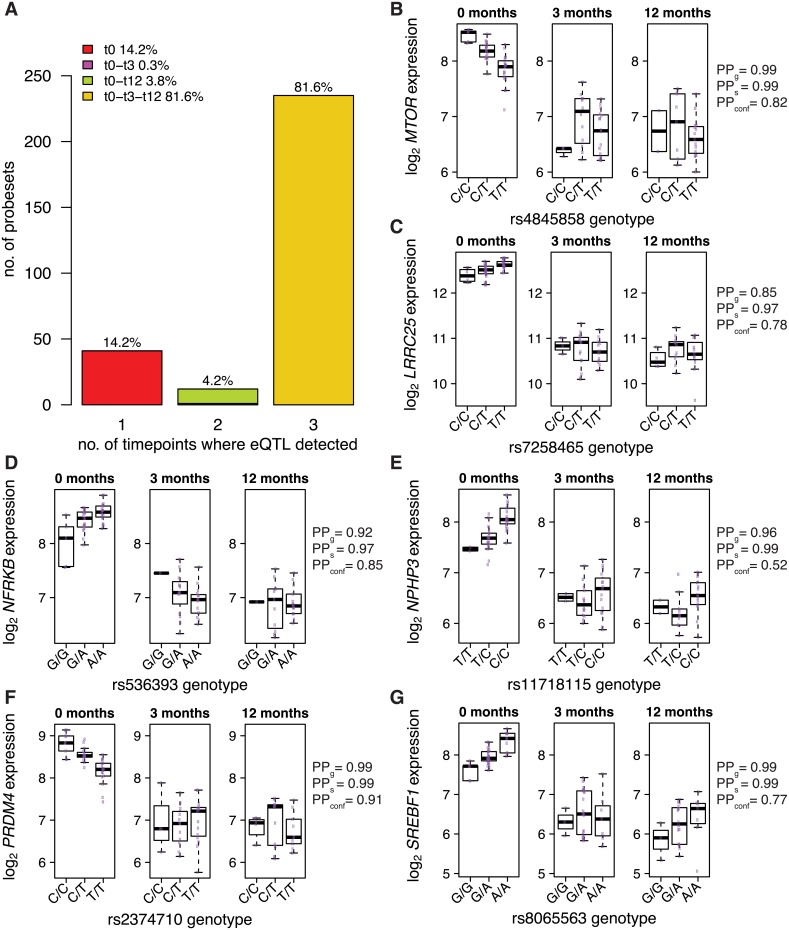
eQTLs present in active inflammatory disease can resolve after treatment. (A) Number of probesets with eQTLs in neutrophils in active AAV and after treatment. (B-G) Examples of neutrophil eQTLs present in active vasculitis which disappear with treatment. PP_g_ = posterior probability that the gene has at least one eQTL in at least one time-point. PP_s_ = posterior probability that the SNP is an eQTL for the gene in at least one timepoint, given that the gene has an eQTL. PP_conf_ = posterior probability for the ‘configuration’ present at time zero and absent at both 3 months and 12 months.

To compare eQTL profiles in AAV pre- and post-treatment more generally, we used the same Bayesian model averaging method (eQTLBMA) that we used for the analysis of cell-type specificity. For this analysis, the subgroups were not cell types, but instead were time points. These were a) time 0 (pre-treatment, when vasculitis is newly diagnosed or flaring), b) time 3 months (3 months into induction therapy), and c) time 12 months (patients on maintenance immunosuppressive therapy). In mononcytes, no time zero-specific eQTLs were identified. In neutrophils, joint analysis of the three timepoints identified eQTLs for 288 probesets corresponding to 262 unique genes (5% Bayes FDR). For 14% of these genes, the ‘best’ model (i.e. the configuration with the highest posterior probability) was that eQTL is only present at time zero (Fig 5A). Examples include *MTOR*, *NPHP3*, and *SREBF1* (Fig 5). Thus we show that eQTLs present in active untreated AAV can disappear after treatment. These eQTLs are probably inflammation-dependent, although their absence post-treatment could reflect drug effects independent of the resolution of inflammation.

### eQTLs provide insights into disease-associated variants from GWAS

To identify the effects of disease-associated SNPs on gene expression, we took SNPs associated with complex traits listed in the National Human Genome Research Institute GWAS catalogue, and proxy SNPs in high linkage disequilibrium (LD) (*r^2^* >0.8), and examined their overlap with eQTL SNPs (eSNPs) from our analyses (IBD-HV S2 Table, AAV S3 Table). We initially focused on IBD as its genetic basis has been extensively studied; a recent GWAS meta-analysis identified 163 independent risk loci [17]. At 34 IBD-associated loci (CD, UC, or both) we identified eQTLs from the analysis of the IBD-HV data that were not previously apparent from eQTL database mining [17] (18 where no eQTL was identified previously, and 16 where we found eQTLs for additional genes; S13 and S14 Figs). When combined with those described in ref. [17], our findings increase the number of IBD-risk loci with eQTLs from 64 to 82.

**Fig 6:**
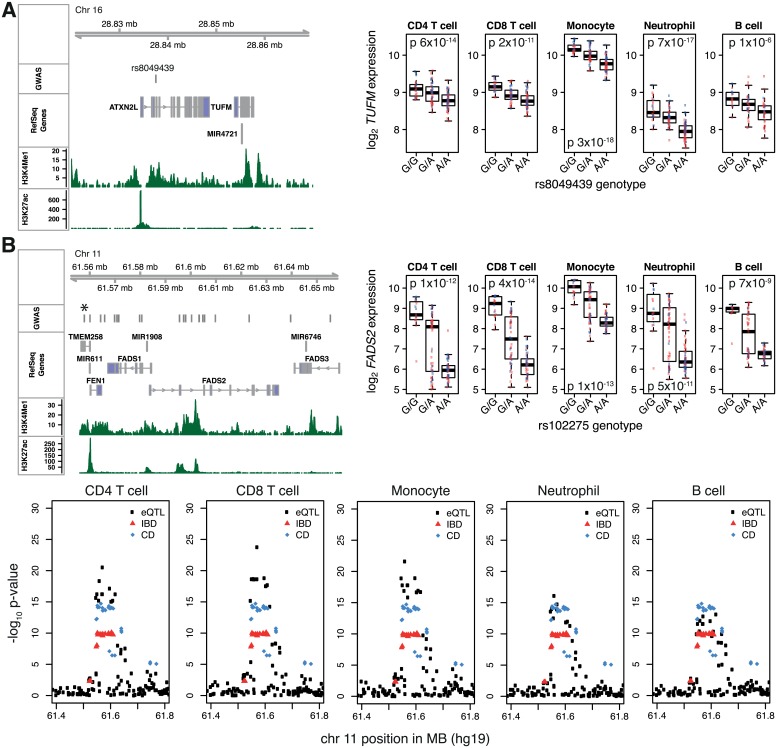
Multiple cell-type analysis provides new insights into effects of IBD-associated SNPs on gene expression. (A) An intronic SNP in *ATXN2L* associated with early-onset IBD is an eQTL for *TUFM* in all cell types examined (FDR <0.0001). Left: Genomic features in the region showing position of the disease-associated SNP, nearby genes, and ENCODE histone marks. Right: eQTL boxplots. Red and blue points indicate IBD patients and HVs respectively. (B) An IBD-associated SNP, rs102275, is an eQTL for *FADS2*. Left: genomic features in the region. “GWAS” indicates disease-associated SNPs from NHGRI GWAS catalogue. *position of rs102275. Right: Crohn’s disease (CD) risk allele (G) is associated with increased FADS2 expression. P-values calculated from score tests. Below: Manhattan plots showing *FADS2* eQTL association signal (black), and IBD and CD association signals from the meta-analysis by Jostins *et al* [17]. eQTL p-values are from linear regression of expression on genotype with disease status as covariate (lm function in R).

Disease-associated SNPs are often assumed to exert their effect through the nearest gene but we found that intronic disease-associated SNPs may instead affect the expression of other genes in the region. For example, rs8049439, an intronic SNP in *ATXN2L* associated with early-onset IBD [18], is an eQTL for the neighbouring *TUFM* in all five cell types examined (Fig 6A), but not for *ATXN2L* itself. These data allow the creation of a revised list of ‘IBD-associated genes’ based not on proximity to disease-associated SNPs, but instead upon the biological effects that these SNPs have on gene expression (S15 and S16 Figs, S4 Table).

Analysis of the direction of effect and cell-type specificity of eQTLs can provide important insights into the role of genetic variation on disease pathogenesis. To illustrate this, three examples will be described. The first demonstrates how detailed eQTL analysis can revise assumptions about genetic contributions to pathogenesis. rs102275, an intronic variant in *TMEM258* associated with Crohn’s disease [19], is an eQTL for *FADS2* in all cell types examined (Fig 6B). *FADS2* encodes fatty acid desaturase 2, a rate-limiting enzyme in the conversion of linoleic acid to pro-inflammatory arachidonic acid. *FADS2* knockout mice develop duodenal and ileocecal ulceration [20], leading to speculation that *FADS2* expression is protective against IBD [19]. However, the risk allele (G) for Crohn’s disease is associated with increased, not decreased, *FADS2* expression (Fig 6B), suggesting that higher *FADS2* expression may in fact increase IBD susceptibility. Simply demonstrating that a SNP is associated with both disease and gene expression is not sufficient to infer causality as there may be two distinct causal variants in LD. We therefore performed colocalisation testing [21] of the eQTL for *FADS2* and for IBD. In all five cell types, this provided strong evidence that the causal variant for the eQTL and Crohn’s disease susceptibility was the same (posterior probability >98%).

**Fig 7:**
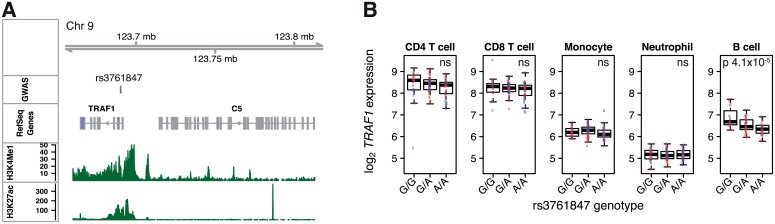
eQTL analysis helps identify genes mediating autoimmune disease risk. (A) *TRAF1*-*C5* locus with genomic features as in Figs 4 and 6. (B) Rheumatoid arthritis susceptibility SNP rs3761847 (risk allele G) is an eQTL for *TRAF1* in B cells. Red and blue points indicate IBD and HV respectively.

Detailed eQTL mapping may also help differentiate between multiple biologically plausible candidate genes. For example, GWAS have identified an association between rheumatoid arthritis and rs3761847, a SNP in an LD block that encompasses two genes that have been implicated in chronic inflammation: *TRAF1* and *C5* [22, 23] (Fig 7A, S17 Fig). Our data reveal that this SNP is an eQTL for *TRAF1* expression in B cells (Fig 7B) and *C5* expression in monocytes. In B cells rs3761847 is the most significant eSNP for *TRAF1* expression (p 4×10^-5^). However, rs3761847 is in weak LD (*r^2^* 0.35) with the most significant eSNP for monocyte *C5* expression (rs10818504, p 3×10^-9^). After conditioning on rs10818504, there is no residual association of the rs3761847 with monocyte *C5* expression (p 0.98), leading to the conclusion that rs3761847 is likely to drive disease susceptibility through *TRAF1* in B cells, and not through modulating *C5*.

**Fig 8:**
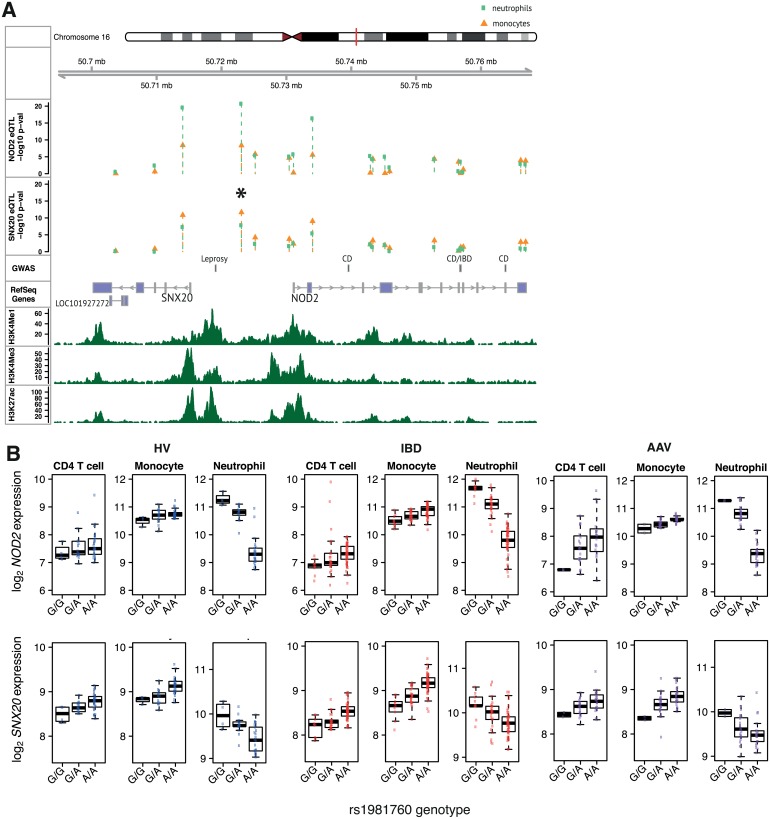
Multiple cell-type eQTL analysis and the genetic aetiology of autoimmune and infectious diseases. An eQTL for both *NOD2* and *SNX20*, with opposing directions of effect in neutrophils versus CD4 T cells and monocytes, at a leprosy-associated locus (*r^2^* 0.922 between eSNP rs1981760 (*) and leprosy-associated SNP rs9302752). (A) Genomic features in the *SNX20*-*NOD2* region. Tracks from top: 1) Chromosome ideogram. Red line indicates the position of the region. 2) Chromosomal position. 3–4) Manhattan plots of association signals for *NOD2* and *SNX20* expression in monocytes and neutrophils (IBD-HV analysis). 5) Disease associated SNPs from the NHGRI GWAS catalogue. 6) RefSeq genes. 7–9) Histone marks from ENCODE. (B) Expression versus genotype boxplots.

Finally, comprehensive eQTL mapping may implicate new candidate genes. NOD2 is central to responses to intracellular pathogens, and SNPs in the *NOD2* region have been linked to leprosy and Crohn’s disease [17, 24]. rs9302752 lies between *NOD2* and *SNX20* in a susceptibility locus for leprosy but not Crohn’s disease, and has been presumed to predispose to leprosy via altering *NOD2* expression [24, 25]. We find an eQTL at this locus not only for *NOD2* but also for *SNX20* in neutrophils, monocytes and CD4 T cells, with the direction of effect on expression of both genes in neutrophils opposite to that in monocytes and CD4 T cells (Fig 8). Independent analysis of the AAV data confirmed the discordant effect of the eQTL in neutrophils. *NOD2* and *SNX20* are transcribed in opposite directions, and so may share a common regulatory element. Consistent with this, the Pearson correlation coefficients between *NOD2* and *SNX20* mRNA levels are 0.69, 0.54, 0.54 in neutrophils, monocytes and CD4 T cells respectively (PEER-adjusted IBD-HV expression data), and ENCODE data suggests active regulatory elements in this region (Fig 8). SNX20 cycles P-selectin glycoprotein ligand-1 (PSGL1) into endosomes, controlling its interaction with the cell adhesion molecules P-, E- and L- selectin on myeloid cells and activated T cells. Whilst *NOD2* remains the leading candidate gene at this locus, the eQTL data indicates consideration should also be given to the role of *SNX20*.

## Discussion

We performed eQTL mapping in the context of either active inflammatory disease or health in five leucocyte subsets that play key roles in the pathogenesis of immune-mediated diseases. Previous reports have suggested that eQTLs are highly specific to immune cell subsets [5]. A naïve interpretation of our data would lead to the same conclusion. Performing eQTL analysis separately in each cell type, and comparing the results obtained from each, suggested that just 5% of genes share eQTLs across all the cell types examined. While closely related cell types appeared to share more eQTLs, even among CD4 and CD8 T cells less than half of eQTLs were detected in both. However, this approach fails to accounts for incomplete power and leads to substantial underestimates of eQTL sharing. When our data are analysed with a more sophisticated joint modelling approach, estimates of the proportion of eQTLs present in all five cell types examined rises to 45%.

eQTL analysis of multiple cell types, whether performed with joint modelling across cell types or through one-at-a-time analysis of each cell type, identified many more eQTLs than analysis of any given single cell type. This highlights the importance of studying eQTLs in relevant cell types, and emphasises that studies using a single tissue or mixed cell populations such as whole blood [26] will fail to detect the effects of many GWAS loci on transcription.

We identified eQTLs with opposing directions of effect between cell types, including eQTLs for *CD52* and *CD101*. Soluble CD52 inhibits activated T cells through SIGLEC-10 [27], while CD101 is an inhibitor of T cell proliferation. This implies that a single genetic variant with contrasting effects on gene expression in different cell types could result in complex downstream impacts on immunity. eQTLs with discordant effects between cell types tended to have smaller effect sizes than eQTLs where the direction of effect was consistent (S8–S11 Figs). We suspect this general pattern is biologically meaningful. eQTLs with large effect sizes that are concordant between cell types may reflect a different underlying regulatory mechanism to the more subtle regulatory variants that can have different directions of effect in different contexts.

Our eQTL analysis provides new insights into disease-associated genetic variants. At 34 IBD-associated loci we identified eQTLs that were not previously apparent from eQTL database mining [17], highlighting the power of studying multiple leucocyte subsets from patients with active inflammatory disease. This eQTL study not only helps prioritise candidate genes, but also provides information on whether disease-associated SNPs up- or down-regulate these genes. This is important because it may challenge current assumptions about the roles such candidate genes play in pathogenesis. For example, our data indicate that higher *FADS2* expression may increase IBD susceptibility, which was not apparent from the phenotype of *FADS2* knock out mice.

This study reveals eQTLs present only in the context of inflammatory disease. For example, we identified a SNP, rs11230584, that is associated with both *CD5* and *CD6* expression in patients with active IBD, but not in healthy individuals. CD5 and CD6 are both transmembrane glycoproteins expressed on T cells and on the B1 subset of B cells. CD5 acts a negative regulator of T cell receptor signalling [28]. In addition, CD5 expression activates multiple intracellular pathways, leading to the production of the type II cytokines IL-5, IL-10 and IL-13. CD6 is involved in the immunological synapse acting as a T cell co-stimulatory molecule, and anti-CD6 monoclonal antibodies have been used in clinical trials for the treatment of psoriasis [29]. Accordingly, this eQTL may result in genotype-dependent differences in immune responses, and perhaps even in responses to anti-CD6 therapy. In addition to identifying eQTLs dependent on disease state, we also show that eQTLs present in active inflammatory disease can disappear following treatment.

In order to identify inflammatory disease-dependent eQTLs in a statistically rigorous manner, we used a linear model with a genotype × disease (G×D) interaction term. The statistical power to detect interaction effects is lower than for so-called ‘main’ effects (in this study, the marginal effect of genotype on expression). A limitation of this study was the relatively modest sample sizes, and we highlight that we used a 15% FDR for the G×D interaction analysis compared to a 5% FDR throughout the rest of the analyses. Because our power to detect G×D effects was limited, it is likely that a higher proportion of eQTLs identified in our analyses of the IBD and AAV cohorts are disease-specific than is apparent from the results of the interaction analysis. Of note, the proportion of neutrophil-specific eQTLs in the AAV analysis was over twice that in the joint IBD-HV analysis. Given the prominent neutrophil turnover and activation seen in AAV, we hypothesise that this observation may be driven by AAV-specific eQTLs in neutrophils. Whilst eQTLs present only after *in vitro* activation of immune cells have been described [13–15], such experiments are unphysiological and cannot fully recapitulate the complexity of cellular activation *in vivo*. The eQTLs we identified, using relevant tissues in the context of *in vivo* inflammation, are important to the appropriate interpretation of the functional effects of SNPs identified through GWAS.

Clinical outcomes vary considerably between patients with the same autoimmune disease, with some individuals experiencing a relatively indolent course, while others suffer from more aggressive disease. The majority of genetic studies to date have focussed on identifying genetic variants that predispose to disease, but candidate SNP studies have revealed differential outcomes according to genotype in Crohn’s disease, rheumatoid arthritis, malaria and tuberculous meningitis [30, 31]. Genetic variants that drive outcome once disease is established may only act as eQTLs in patients with active inflammatory disease, and not in healthy individuals. Therefore inflammatory-disease specific eQTLs, such as the eQTL for *CD5* and *CD6*, may be important for understanding the heterogeneity of clinical course in autoimmune and infectious diseases.

In summary, we reveal that the consequences of genetic variation on gene expression are complex, and may vary between cell types, between health and inflammatory disease, and before and after treatment. This study, using parallel eQTL mapping in a broad panel of relevant cell types from both healthy individuals and patients with active inflammatory disease, provides increased understanding of the biology underlying the genetic basis of immune-mediated diseases.

## Methods

### Ethics statement

Ethical approval for this work was obtained from the Cambridgeshire Regional Ethics Committee (REC08/H0306/21). All participants provided written informed consent.

### Subjects

We recruited a cohort of adult healthy volunteers, free from autoimmune disease or any other chronic condition and not taking any regular medication at the time of enrolment. Adult patients with active inflammatory bowel disease (Crohn’s disease or ulcerative colitis) were recruited from a specialist inflammatory bowel disease clinic at Addenbrooke’s hospital in Cambridge, UK, prior to commencing treatment, as described previously [32]. Diagnosis was made using standard endoscopic, histopathological, and radiological criteria. Patients receiving immunomodulators or corticosteroids were excluded to avoid potential confounding effects on gene expression. Patients with a diagnosis of ANCA-associated vasculitis (either granulomatosis with polyangiitis, formerly Wegener’s granulomatosis, or microscopic polyangiitis, but not eosinophilic granulomatosis with polyangiitis, formerly Churg-Strauss syndrome) and evidence of active disease were recruited from a specialist clinic at Addenbrooke’s Hospital. Active disease was defined by Birmingham Vasculitis Activity Score (BVAS), elevated acute phase markers, and the clinical assessment that induction immunosuppression would be required to manage disease, as described previously [33]. 27 of 46 AAV patients were venesected at the time of first clinical presentation prior to treatment. The remainder had clearly defined flares of previously quiescent disease on stable maintenance therapy, and required treatment with induction immunosuppression. See S5 Table for more detailed demographics.

### Immune cell subsets

Cell separations were performed using positive selection with magnetic-activated cell sorting (MACS) as previously described [34]. Blood samples (100 ml) were collected into 4% sodium citrate and PBMC isolated by centrifugation over Histopaque 1077 (Sigma). The PBMC sample was split into two aliquots and CD14 monocytes were isolated from one aliquot and CD19 B cells from the other by magnetic cell sorting using CD14 and CD19 microbeads (Miltenyi Biotec) according to the manufacturer’s instructions. CD4 and CD8 T cells were then isolated from the CD14 and CD19 negative fractions, respectively, by magnetic cell sorting using CD4 and CD8 microbeads as described by the manufacturer. CD16 neutrophils were obtained from the red cell/granulocyte pellet by red cell lysis followed by positive selection using CD16 microbeads as described by the manufacturer.

### Nucleic acid extraction

RNA was extracted from cell lysates using either Qiagen RNEasy Mini Kits or Qiagen Allprep kits according to the manufacturer’s instructions. RNA quality was assessed using an Agilent Bioanalyser 2100 (Agilent Technologies) and quantified by spectrophotometry using a NanoDrop ND-1000 spectrophotometer (Thermo Scientific). Genomic DNA was extracted using Qiagen Allprep kits according to the manufacturer’s instructions and quantified as above.

### Genomic DNA genotyping and genotype data quality control (QC)

Patients with IBD and healthy controls were genotyped using the Illumina Human OmniExpress12v1.0 BeadChip at the Wellcome Trust Sanger Institute according to the manufacturer’s protocol. After genotype calling with Illumina GenCall software, SNP data was processed in the following sequence using PLINK [35]. Samples with a sex mismatch, abnormal heterozygosity, proportion of missing SNPs >5%, and duplicated or related samples (identified using Identity by State) were removed. SNPs with missing calls >5%, extreme deviation from Hardy-Weinberg Equilibrium (p-value <1×10^-8^), or which were monomorphic were removed. Finally, principal components analysis of the post-QC genotype calls combined with calls from HapMap3 founder individuals (downloaded from http://hapmap.ncbi.nlm.nih.gov/), using the R/Bioconductor snpStats package, confirmed all samples were of European ancestry (S18 Fig). Genotype data was read into R version 3.02 (http://www.R-project.org/) using the snpStats package, and stored as a SnpMatrix object. SNPs with minor allele frequency (MAF) <5% were excluded from eQTL analysis, leaving 626,858 autosomal SNPs. These data have been deposited in the European Genome-phenome Archive (EGA) and are available on request (EGAS00001001251).

Genotype data for AAV patients were available from a previous GWAS [36]. Briefly, genotyping was performed by AROS Applied Biotechnology (Aarhus, Denmark) using the Affymetrix SNP 6.0 Platform. SNP genotypes were called using the CRLMM algorithm [37], and standard QC performed as described previously [36].

### Gene expression measurement, pre-processing and QC

200 ng RNA was processed for hybridization onto Affymetrix Human Gene ST 1.1 microarrays, according to the manufacturer’s instructions, prior to scanning. Microarrays were processed with the automated GeneTitan instrument which uses a 96-well plate. Chip probe intensity files (.CEL) were read into R version 3.02 using the Bioconductor oligo package [38]. Raw intensity values were pre-processed with the Robust Multi-array Average (RMA) algorithm which performs background correction, quantile normalization, log_2_ transformation and summarization to gene level via a median polish. Probe annotation was obtained from the pd.hugene.1.1.st.v1 Bioconductor package. The resulting expression matrix and phenotype data was stored as an ExpressionSet object. The raw intensities for the expression data for each cell type were read in and normalised separately. The arrayQualityMetrics package was run on the normalised expression matrices before and after batch correction with ComBat (see below) and poor quality samples removed. Microarray data are available in the ArrayExpress database (www.ebi.ac.uk/arrayexpress) under accession number E-MTAB-3554.

### Removal of unwanted expression heterogeneity

Principal components analysis of the expression matrices indicated batch effects. To address this, we used two alternative approaches. The first approach was removal of unwanted expression heterogeneity by adjustment for latent factors using Probabilistic Estimation of Expression Residuals (PEER) [39]. The use of PEER has increased eQTL discovery compared to standard methods in a number of studies [4, 40]. PEER can model both known confounders and hidden latent factors in the expression data. We ran PEER specifying batch and sex as known confounders, and used the expression residuals as the dependent variable in the subsequent eQTL scan. For the analysis of cell-type specificity, comparison of direction of effect between cell types, and for the intersection of eQTLs with GWAS SNPs, we used eQTLs detected from association scans using PEER residuals.

Alternatively, we performed batch correction using the ComBat function from the sva (Surrogate Variable Analysis) Bioconductor package [41]. ComBat was implemented specifying diagnosis as a covariate of biological interest whose effect should be preserved. This method was used for the genotype × disease interaction analysis and the pre- and post-treatment analysis.

### Samples used to create expression matrices

The GGtools Bioconductor package [42] was used to combine SnpMatrix and ExpressionSet objects for patients for whom we had both good quality genotype and expression data to create an smlSet object. Table 1 shows the numbers of samples available for analysis by disease and cell type. For the Bayesian joint analysis across cell types presented in Fig 1, we used all available samples, even if this meant differing sample sizes for each cell type. For the estimates of eQTL cell-type specificity from one-at-a-time cell-type analysis, we confined eQTL mapping to individuals with expression data available for all cell types to ensure equal power for each cell type. In the latter scenario, subsetting was performed after pre-processing and adjustment for latent factors or batch correction. In order to make direct comparisons with results from separate cell-type analysis, we also used the Bayesian method on the subset of individuals with expression data available for all cell types (S19 Fig). For the detection of eQTLs to intersect with GWAS SNPs, we used all available samples to maximise power, even if this meant differing sample sizes for each cell type. The expression versus genotype boxplots in Figs 2, 3, 6, 7 and 8 show all individuals for whom we have good quality expression and genotype data rather than restricting to the subset of individuals with expression data available for all cell types.

### Filtering the expression data

Filtering of probesets in the expression data was performed prior to the eQTL scan. Filtering of expression data has been shown to increase power to detect differentially expressed genes, and should not bias FDR estimates as long as the variable of interest is not used in the filtering step [43]. Probesets on sex chromosomes and those which did not map to an Entrez gene ID were removed. The remaining probesets were then filtered by variance (Bioconductor genefilter package). As well as increasing power, filtering by variance has two additional benefits. First, genes which are expressed homogeneously are not informative in an eQTL analysis. Second, unexpressed genes will show little variability between samples and thus filtering on the basis of lack of expression variability is a reliable method for detecting and excluding unexpressed genes from further analysis [44]. In the downstream analysis we compared eQTLs detected in each cell type. In order make these comparisons valid, it was necessary to perform eQTL mapping on the same filtered list of probesets in each cell type. To achieve this, we retained any gene whose variance across samples was in the upper quartile in any one of the cell types. For the joint IBD-HV analysis, this resulted in 9,041 probesets when using all available samples, and 9,242 when restricting to the 65 individuals with expression data available for all cell types. For the AAV analysis, the same filtering procedure resulted in 8,916 probesets when using all available samples, and 8,979 when restricting analysis to the 33 patients with complete expression data available for CD4 T cells, CD8 T cells, monocytes and neutrophils.

### Analysis limited to 5,186 probesets expressed in all cell types

This analysis was performed using the IBD-HV dataset, restricted to the individuals with expression data available for all 5 cell types (n = 65). To identify probesets targeting robustly expressed genes in each cell type, the following procedure was performed. First we calculated the mean expression of each probeset across all samples from a given cell type (using expression data that had been RMA-normalised and then batch-corrected with ComBat). To determine thresholds for declaring probesets as expressed or non-expressed in that cell type, we used the normalmixEM function from R package mixtools to fit a mixture of two Gaussian distributions to the vector of mean probeset expression values. Starting values for the expectation–maximization (EM) algorithm were chosen assuming 50% of expressed probesets, and the initial mean values of the underlying Gaussian distributions were set equal to the first and third quartile of the probeset expression distribution. The distribution with the lowest mean expression was considered as that corresponding to non-expressed genes. A detection threshold was set by taking the 95th percentile of this distribution. Probesets whose mean expression was above the detection threshold were considered to be expressed in that cell type. Subsequently, probesets that did not map to an Entrez gene ID were removed. The lists of probesets expressed in each cell type were then intersected to find those expressed in all cell types. Probesets on the sex chromosomes, and those which did not map uniquely to an Entrez ID were removed. This resulted in 5,186 probesets. The matrix of PEER-adjusted expression data was then subsetted to retain only these probesets, and the eQTL scan was performed using the residuals from PEER as the dependent variable.

### eQTL mapping

We performed *cis* eQTL mapping with the All.cis function in the GGtools Bioconductor package. Previous work has suggested that the most *cis* eQTLs are approximately centred around the transcribed region, with few eQTLs lying more than 50kB from the gene [6, 7, 45]. For each gene, we defined *cis* SNPs as those lying in the region spanning 100kB up and downstream from the gene start and end positions respectively. SNP location information was obtained using the Bioconductor package SNPlocs.Hsapiens.dbSNP.20120608 (dbSNP Build 137). Sensitivity analysis using varying distances to define *cis* found no increase in eQTL discovery for *cis* distances greater than 100 kB using a 5% FDR significance threshold.

The All.cis function calculates association statistics (score tests) for each *cis* SNP-transcript pair. Specifically, the function fits a generalized linear model with transcript abundance as the continuous dependent variable and genotype as the predictor variable. Optionally, potential confounders of an expression-genotype association can be included as covariates. Genotype is a discrete variable coded 0, 1, or 2 according to allelic dose (i.e. homozygous SNP genotypes are coded 0 or 2 and heterozygous genotypes are coded 1). For each gene, each SNP in the *cis* region was tested in turn for association with mRNA abundance (after adjustment with PEER) using an additive genetic model; after the base model was fitted (i.e. a model with an intercept and any specified covariates), a score test was then performed for addition of genotype to the model. The tests used are asymptotic chi-squared tests based on the vector of first and second derivatives of the log-likelihood with respect to the parameters of the additive model. Where the dataset comprised more than one disease grouping (i.e. IBD and healthy individuals), the diagnostic grouping was included as an additional covariate. We did not include sex as a covariate, as sex had already been specified as a known confounder when running PEER.

GGtools provides estimates of the FDR through estimation of the distribution of statistics under the null by breaking expression-genotype relationships through permutation of sample labels (the ‘plug-in FDR’ method [46]- see S1 Text). A 5% FDR was used to declare statistical significance.

### *cis* eQTL cell-type specificity

To evaluate eQTL cell-type specificity, we used the eQTLBMA package which implements the method described by Flutre *et al* [16]. We ran this according to the instructions in the user manual. Briefly, we first transformed the expression level of each gene into the quantiles of a standard Normal distribution. Ties were broken randomly. We then ran the eqtlbma_bf programme, to compute the Bayes factors assessing the support in the data for each probeset-SNP pair being an eQTL. We analysed the IBD patient and HV data together, specifying disease status as a covariate. To maximise power, we used all available samples (from 91 IBD patients and 43 HVs), even if this meant that for some individuals we did not have full expression data for all cell types. In this scenario we used the option --error hybrid. Samples from AAV patients were analysed separately to avoid difficulties that might arise from combining genotype data from a separate platform. Again, we used all available samples. The output file containing the Bayes factors was then fed into the eqtl_hm programme, to fit the hierarchical model with an EM algorithm, and get to maximum-likelihood estimates of hyper-parameters and the configuration probabilities. To obtain the posterior probabilities, we need an estimate of the probability for a gene to have no eQTL in any tissue, *π*_0_. To do this, we implemented the EBF procedure, using the gene-level Bayes factors averaged over the grid and configuration weights (estimated via the EM algorithm). Finally we ran eqtlbma_avg_bfs to obtain (i) the posterior probability (PP) for the gene to have an eQTL in at least one cell type, (ii) the PP for a SNP to be “the” eQTL, assuming one eQTL per gene, (iii) the PP for the SNP to be an eQTL, (iv) the PP for the eQTL to be active in a given tissue, and (v) the PP for the eQTL to be active in a given configuration. We used a PP corresponding to a 5% Bayes FDR as the significance threshold.

To evaluate estimates of eQTL cell-type specificity from one-at-a-time cell-type analysis, we limited the IBD-HV analysis to 65 individuals (47 IBD patients, 18 HVs) in whom samples passing expression quality control were available for all five cell types. By using the same set of individuals, the sample size and genotype matrix (predictor variables) were identical for each cell type. In order to make a fair comparison between the results from the one-at-a-time cell-type analysis and those from eQTLBMA, we re-ran eQTLBMA on these same 65 individuals with full expression data for all 5 cell types. In this latter scenario we used the option --error mvlr (S19 Fig and S1 Text).

### Discovery of eQTL with opposing directions of effects between cell types

For all possible pairings of the cell types, we took those eSNP-gene associations that were statistically significant in both members of the pair in the one-at-a-time cell-type analyses (FDR <0.05), and compared the direction of effect on expression in each. We did this by plotting the estimated coefficient (beta) for the genotype term in the first cell type against that in the second. The betas were estimated from regressing PEER-adjusted expression on genotype using the lm function in R. For the IBD-HV analysis, disease status was included as a covariate. eQTLs with opposing directions of effect between cell types have a positive beta in one cell type but a negative beta in the other (see S1 Text for more detailed discussion).

### Genotype × disease interaction analysis

To identify eQTLs influenced by the presence or absence of inflammatory disease we analysed the joint IBD-HV dataset using a linear model with a genotype × disease interaction term. Expression data for each cell type was RMA normalised separately, but within each cell type expression data from healthy individuals and IBD patients were normalised together. We used expression data that had been batch-corrected with ComBat, as adjustment for latent factors with PEER might remove genuine disease effects. We again restricted testing for each gene to *cis* SNPs. For each gene-SNP pair, a linear model was fitted using expression as the dependent variable, and genotype (coded 0,1, or 2), disease status (coded 0 and 1 for healthy and IBD respectively), and a genotype × disease interaction term as the predictor variables. This was performed in R using the lm function.

Fitting a model with an interaction term is statistically more robust than the naïve approach of separate analysis of IBD and HV cohorts, followed by comparison of the resulting lists to find eQTLs common to both groups, and those identified only in IBD or only in HVs. The latter approach would result in an excess of false declarations of group-specific eQTLs. In particular, the comparison of lists approach is unduly influenced by the difference in statistical power to detect eQTLs in each group when the group sample sizes are different. The IBD group was larger than the HV group, and so there was greater power to detect eQTLs in the former. This would have led to many false declarations of IBD-specific eQTLs had we used the comparison of lists approach (see S1 Text for more detailed discussion).

The analysis was performed for CD4 T cells, CD8 T cells, monocytes and neutrophils. B cells were not analysed due to the low number of samples available from healthy individuals. For each cell type, we used all available IBD and HV samples (see Table 1 for sample sizes). We limited testing of SNPs to those with MAF of 10% or higher, as power decreases with decreasing MAF. Probesets were filtered in the following order: first, removal of probesets that were non/lowly expressed in that cell type, and those which did not map to an Entrez gene ID; second, filtering of the remaining probesets by variance to retain only those in the upper 40%.

Interaction effects are harder to detect than main effects, so to improve our power we employed a ‘two-step’ procedure adapted from methods used to detect genotype-environment interactions in GWAS [47]. ‘Two-step’ procedures aim to reduce the multiplicity burden associated with having a large number of variables to explore by applying some filter (step 1) on the number of variables actually tested (step 2). In order to prevent biasing of FWER/FDR estimates, the filtering procedure used in step 1 should be independent of the final test statistics in step 2. In step 1 we performed linear regression of expression on genotype (main effect of genotype), with no covariates. Importantly, by not including disease status as a covariate, we ensured independence of the step 2 statistics [47]. SNP-gene pairs passing the step 1 threshold *α*_1_ were eligible for step 2 testing. We used a p-value of 5×10^-5^ for *α*_1_. A more liberal *α*_1_ allows more SNP-gene pairs through to step 2, but at the price of more multiple testing and a more stringent threshold in step 2. In addition, because there were multiple correlated SNPs due to LD, where multiple SNPs were significantly associated with the same gene at the threshold *α*_1_, only the most significant SNP per gene was taken forward to step 2.

In step 2, we fitted the full model with regression of expression on genotype, disease and a genotype × disease interaction term. P-values for the interaction terms were then adjusted for the number of tests performed in step 2 with the p.adjust function in R using the Benjamini-Hochberg procedure, and a 0.15 FDR threshold used to declare significance. Q-values were also calculated.

### Comparison of eQTLs in AAV pre- and post-treatment

Each cell type was analysed separately. We used eQTLBMA to jointly analyse expression data from the 3 timepoints: baseline, 3 months, and 12 months. We used we used the option --error hybrid as the individuals in each subgroup were overlapping but not identical. For genes with a significant eQTL (5% Bayes FDR), we took the SNP with the highest posterior probability for being the eQTL. For this list of SNP-gene associations, we then assessed the posterior probabilities for each of the 2^3^ − 1 = 7 possible configurations.

### Intersection of eQTLs with GWAS hits

To identify the effects of disease-associated SNPs on gene expression, and how this varies between cell types, we examined the overlap of SNPs associated with complex traits from the NIH National Human Genome Research Institute GWAS catalogue (http://www.genome.gov/gwastudies/) (download date 20 May 2015) with eQTL SNPs (eSNPs) discovered in each cell type in our analysis. The SNPs identified in eQTL studies or GWAS are not necessarily themselves causal variants, but instead may be in LD with them. Therefore GWAS tag SNPs, and proxy SNPs in high LD with them (*r^2^* >0.8), were cross-referenced with *cis* eQTLs found in each cell type. SNAP was used to identify proxy SNPs (http://www.broadinstitute.org/mpg/snap/), using the 1000 Genomes (http://www.1000genomes.org/about) pilot 1 data (CEU individuals) as the reference population.

To define the eSNPs for cross-referencing, we repeated the analysis using two levels of stringency for declaring colocalisation of eQTL and GWAS signals. For the basic criteria, a GWAS signal was declared to colocalise with the eQTL if the GWAS SNP or one of its proxies was a significant eQTL (FDR <0.05). For the more stringent criteria, in addition to the basic criteria, the GWAS SNP or one of its proxies had to be the most significant *cis* eSNP for that gene (S1 Text, S16 Fig).

For the detailed examination of the effects of IBD-associated SNPs on gene expression, we took SNPs identified in the IBD GWAS meta-analysis [16] as hits in either Crohn’s disease (CD), ulcerative colitis (UC) or both (IBD) (Supplementary Table 2 of that paper), and cross-referenced these SNPs and proxies (*r^2^* >0.8) with the eQTL SNPs from our analysis as above (see S1 Text).

### Colocalisation testing

Colocalisation testing was performed using the coloc package [21]. We used the publically available summary statistics for IBD, CD and UC GWAS meta-analysis by Jostins *et al* [17] along with our eQTL data.

### Data availability

1) Expression data has been deposited into ArrayExpress (accession number E-MTAB-3554). 2) Genotype data has been deposited into the EGA (study accession number EGAS00001001251, url https://www.ebi.ac.uk/ega/studies/EGAS00001001251). Controlled access for scientific researchers is available via requests to the data access committee (EGAC00001000338). 3) Summary statistics for all significant eQTLs for each cell type and dataset are included as a compressed tarball (S1 Data).

## Supporting Information

S1 Fig Estimates of eQTL cell-type specificity from separate cell-type analysis and ‘comparison of lists’. Analysis using PEER-adjusted expression data. **(a)** Overlap in genes with significant eQTLs (FDR <0.05) according to cell type in the joint IBD-HV analysis (n = 65), and the AAV analysis (n = 33). **(b)** Number of genes with an eQTL, subsetted according to the number of cell types in which the eQTL was detected. The bar for eQTLs detected in only one cell type is subdivided according to which cell type the eQTL was detected in. The denominator for the percentages shown is the total number of genes for which an eQTL was detected in at least one cell type. **(c)** Jaccard coefficients (as %) for similarity in eQTL profiles between all possible pairwise comparisons of cell types.
(PDF)

S2 Fig Overlap in significant (*cis*) SNP-gene associations (FDR <0.05) according to cell type (IBD-HV data) using separate cell-type analysis. In contrast, S1 Fig shows the number of *genes* with eQTLs in each cell type. Analysis restricted to 65 individuals (IBD patients and HVs) with expression data available for all cell types. PEER residuals were used in the eQTL scans, with disease as a covariate.
(PNG)

S3 Fig Estimates of eQTL sharing across leucocyte subsets from joint modelling of expression data across cell types (eQTLBMA), restricted to 93 individuals (IBD-HV data) with complete expression data for CD4 T cells, CD8 T cells, monocytes and neutrophils. **(a)** Number of probesets with an eQTL, subsetted according to the number of cell types in which the eQTL was declared present. Each bar is subdivided according to which cell type the eQTL was detected in. The denominator for the percentages shown is the total number of genes for which an eQTL was detected in at least one cell type (5% Bayes FDR). Analysis using PEER-adjusted expression data. %s rounded to 1 d.p. **(b)** Jaccard coefficients, as %. Key: CD4 = CD4 T cells, CD8 = CD8 T cells, CD14 = monocytes, CD16 = neutrophils.
(PDF)

S4 Fig Examples of cell-type- and lineage-specific eQTLs from Fig 2B showing expression values after adjustment with PEER. For ease of biological interpretation, in Fig 2B boxplots of the RMA-normalised expression values *before* adjustment with PEER were shown. Here we show expression values after adjustment of the log_2_ RMA-normalised expression values for batch, sex and latent factors with PEER. It should be noted that these PEER-adjusted expression values, and not the expression values in Fig 2B, were used in the eQTL scan, with disease status included as a covariate. Blue and red dots represent healthy volunteers and IBD patients respectively. Sample sizes: CD4 T cell 121, CD8 T cell 108, monocyte 124, neutrophil 121, and B cell 80. ns = not significant using a 5% FDR significance threshold. **(a)** Monocyte-specific, **(b)** myeloid-specific, and **(c-d)** T lymphocyte-specific eQTLs.
(PDF)

S5 Fig Jaccard coefficients for eQTL sharing between cell types from the analysis using eQTLBMA. **(a)** IBD-HV analysis (n = 134). **(b)** AAV analysis (n = 46). All available samples were used, even if expression data was missing for some cell types. For Jaccard coefficients from the analysis restricted to 65 individuals with complete expression data across all 5 cell types, see S19 Fig.
(PDF)

S6 Fig Estimates of eQTL ‘sharing’ using eQTLBMA, after permutation of CD4 T expression data. **(a)** Number of probesets with an eQTL, subsetted according to the number of cell types in which the eQTL was declared present (taking the cell-type configuration with the highest posterior probability). **(b)** Jaccard coefficients, as %.
(PDF)

S7 Fig eQTL cell-type specificity is partly driven by lack of expression in certain cell types. Estimates of eQTL sharing from eQTLBMA, with analysis limited to probesets expressed in all 5 cell types. This analysis was performed on the 65 individuals (IBD patients and HVs) with expression data available for all cell types. PEER residuals were used in the eQTL scans, with disease status as a covariate. **(a)** Number of probesets with an eQTL, subsetted according to the number of cell types in which the eQTL was declared present. Each bar is subdivided according to which cell type the eQTL was detected in. The denominator for the percentages shown is the total number of probesets for which an eQTL was detected in at least one cell type. **(b)** Jaccard coefficients for eQTL sharing (as %).
(PDF)

S8 Fig Detection of eQTLs with opposing directions of effects between pairs of cell types using the subset of IBD patients and HVs for whom we have expression and genotype data in CD4 and CD8 T cells, monocytes and neutrophils (n = 93). Each point represents a SNP-gene association that was statistically significant in both cell types (FDR <0.05). For each SNP-gene association the axes show the estimated effect size (beta) in each cell type. eQTLs with opposing directions of effect between cell types have a positive effect size in one cell type but a negative one in the other (points in the upper left and lower right quadrants). The names of genes with such eQTLs are printed in red. As a consequence of LD, there are sometimes multiple eSNPs significantly associated with one gene, so there are more points in the upper left and lower right quadrants than gene names printed on the plot.
(PDF)

S9 Fig Detection of eQTLs with opposing directions of effects between pairs of cell types using the subset of IBD patients and HVs for whom we have expression and genotype data in all *five* cell types (n = 65). In contrast in S8 Fig we showed the n = 93 individuals for whom we had complete data across CD4 and CD8 T cells, monocytes and neutrophils. Each point represents a SNP-gene association that was statistically significant in both cell types (FDR <0.05).
(PDF)

S10 Fig Effect sizes by cell type in the analysis of the IBD cohort alone, using all available IBD samples. N = 79, 67, 83, 82 and 60 for CD4 T cells, CD8 T cells, monocytes, neutrophils and B cells respectively.
(PDF)

S11 Fig Effect sizes by cell type in the analysis of the AAV cohort alone, using all available AAV samples. N = 41, 40, 45, and 43 for CD4 T cells, CD8 T cells, monocytes and neutrophils respectively.
(PDF)

S12 Fig Illustration of genotype × disease (G×D) interaction effects. Genotype is coded 0,1 or 2 according to the number of copies of the minor allele.
(PDF)

S13 Fig IBD-associated SNPs that overlap with eQTLs. SNPs identified in the IBD meta-analysis by Jostins *et al* [17] as associated with IBD (both CD and UC) that are eQTLs are listed. SNPs are ordered by chromosome and position. Red blocks indicate that the SNP, or a proxy in high LD (*r^2^* >0.8), is an eQTL in our analysis. eQTLs identified in ref. [17] are shown in grey. An asterix indicates additional information added by our analysis (either an eQTL where none was reported, or where we find the SNP is an eQTL for a different gene in at least one cell type from those previously identified; such novel candidate genes are indicated in bold). SNPs where neither we nor previous database mining [17] identify an eQTL are included in S4 Table. SNPs associated with Crohn’s disease or ulcerative colitis (but not both) that are eQTLs are shown in S14 Fig.
(PDF)

S14 Fig SNPs associated with Crohn’s disease (CD) or ulcerative colitis (UC) (but not both) which are eQTLs. **(a)** CD-specific SNPs from the IBD GWAS meta-analysis by Jostins *et al* [17] which are eQTLs. **(b)** UC-specific SNPs which are eQTLs. SNPs associated with both CD and UC are shown in S13 Fig. SNPs were taken from Supplementary Table 2 of the IBD GWAS meta-analysis by Jostins *et al* [17], and are ordered by chromosome and position. Red blocks indicate that the SNP or a proxy in high LD (*r*^2^ >0.8) is an eQTL in our analysis (using PEER-adjusted expression data and all available IBD and HV samples, i.e. not restricted to the individuals for whom genotype and expression data was available in all cell types). The gene(s) whose expression is associated with that SNP are printed on the plot. eQTLs identified through database mining in ref. [17] are shown in grey. Any SNP highlighted with an asterix indicates additional information added by our analysis (either an eQTL where none was reported in ref. [17], or where we find the SNP is an eQTL for a different gene in at least one cell type from those previously identified; such novel candidate genes are indicated in bold). SNPs where neither we nor ref. [17] identify an eQTL are not shown here, but are included in S4 Table.
(PDF)

S15 Fig Genes whose expression is associated with IBD susceptibility SNPs. We took eQTL SNPs identified in each cell type in the joint HV-IBD analysis (using PEER-adjusted expression data, FDR <0.05), and intersected them with the list of IBD-associated SNPs from the NHGRI GWAS catalogue and proxies in high LD (*r*^2^ >0.8). We thus identified SNPs which are both eQTLs and are associated with IBD. Here we show the genes whose expression is associated with these SNPs in each cell type. Grey shading indicates the gene has a *cis* eQTL which is an IBD hit or one of its proxies. The ordering of the cell types and genes is the result of hierarchical clustering. Here we use all eSNPs passing the 5% FDR significance threshold as the eQTL SNPs. In contrast, S16 Fig uses only the *best* eQTL per gene.
(PDF)

S16 Fig Overlap of IBD-associated SNPs with eQTLs: more conservative definition for overlap (genes whose best eQTL is an IBD susceptibility SNP). From the list of significant eQTL SNPs (FDR <0.05, joint HV-IBD analysis, PEER-adjusted expression data), we took only the best *cis* eQTL SNP per gene (in contrast with S15 Fig where we used all significantly associated SNPs). We intersected these SNPs with the list of IBD-associated SNPs from the NHGRI GWAS catalogue and proxies in high LD (*r*^2^ >0.8). Grey shading indicates the gene’s best *cis* eQTL is an IBD GWAS hit or one of its proxies. The ordering of the cell types and genes is the result of hierarchical clustering.
(PDF)

S17 Fig LD block around the rheumatoid arthritis-associated SNP (rs3761847) in the *TRAF1-C5* region. **(a)** LD structure visualised using Haploview. **(b)** *r*^2^ to rs3761847 in CEU population (plot made using SNAP https://www.broadinstitute.org/mpg/snap/). Positions shown are hg18 genome build.
(PDF)

S18 Fig Principal components analysis of the genotype data from IBD patients and HVs, and from HapMap individuals. Each point represents an individual. Our samples are coloured black. Ethnicity of HapMap individuals is indicated by the legend in the bottom left corner.
(PDF)

S19 Fig Estimates of eQTL sharing from eQTLBMA, with analysis restricted to the 65 individuals (IBD-HV dataset) with complete expression data across all 5 cell types. **(a)** eQTLs divided according to their ‘best’ configuration across cell types. **(b)** Jaccard coefficients, as %.
(PDF)

S20 Fig Distribution of Shannon entropies according to the ‘best’ configuration for eQTL presence/absence across cell types. The Shannon entropy is a measure of uncertainty, where a higher entropy indicates more uncertainty. Shannon entropies were calculated on the configuration posterior probabilities for the best SNP for each gene with a significant eQTL (5% Bayes FDR). Results from joint modelling across 5 cell types using eQTLBMA on the 65 individuals (IBD-HV dataset) with complete expression data across all 5 cell types. CD4 = CD4 T cells, CD8 = CD8 T cells, CD14 = monocytes, CD16 = neutrophils, CD19 = B cells.
(PDF)

S1 Table eQTLs with significant genotype × disease interaction terms. eQTL mapping with expression data that were batch-corrected with ComBat. The columns marked ‘pval.bh’, ‘pval.bonf’ and ‘qval’ indicate Benjamini-Hochberg adjusted p-values, Bonferonni corrected p-values, and q-values respectively.
(XLSX)

S2 Table Overlap of eQTL SNPs identified in the joint IBD-HV analysis (using PEER-adjusted expression data) with GWAS SNPs from the NHGRI GWAS catalogue. Columns labelled ‘Disease.trait’, ‘PUBMEDID’, ‘Mapped gene’ and ‘Context’ are taken from the.txt file download of the NHGRI GWAS catalogue and refer to the SNP in the ‘gwasSNP’ column. Columns ‘PROBEID’, ‘ENTREZID’, and ‘SYMBOL’ refer to the probeset and corresponding gene for which the SNP (‘eSNP’) is an eQTL. The LD (*r*^2^) between the eSNP and the GWAS tag SNP is indicated in the column ‘RSquared’. The column labelled ‘scores’ shows 1 degree of freedom chi-squared scores.
(XLSX)

S3 Table Overlap of eQTL SNPs identified in the AAV analysis (using PEER- adjusted expression data) with GWAS SNPs from the NHGRI GWAS catalogue. Columns labels as per S2 Table.
(XLSX)

S4 Table Genes whose expression is associated with IBD-associated SNPs or proxy SNPs in high LD. SNP lists taken from Supplementary Table 2 of the paper by Jostins *et al* [17], as described in S1 Text. The column marked ‘snp.type’ provides SNP annotation from SNPnexus. ‘NA’ indicates no gene found to be significantly associated with the disease-associated SNP.
(XLSX)

S5 Table Sample breakdown by sex and diagnosis, indicating for which cell types expression data were available. (XLSX)

S1 Text Supplementary Information. (PDF)

S1 Data Supplementary Data. Compressed tarball containing a folder of eQTL summary statistics from each analysis.
(GZ) Click here for additional data file.

## References

[1] Hindorff LA, Sethupathy P, Junkins HA, Ramos EM, Mehta JP, Collins FS, et al Potential etiologic and functional implications of genome-wide association loci for human diseases and traits. Proc Natl Acad Sci USA. 2009 6;106(23):9362–9367. 10.1073/pnas.0903103106 19474294 PMC2687147

[2] Maurano MT, Humbert R, Rynes E, Thurman RE, Haugen E, Wang H, et al Systematic localization of common disease-associated variation in regulatory DNA. Science. 2012 9;337(6099):1190–1195. 10.1126/science.1222794 22955828 PMC3771521

[3] Dimas AS, Deutsch S, Stranger BE, Montgomery SB, Borel C, Attar-Cohen H, et al Common regulatory variation impacts gene expression in a cell type-dependent manner. Science. 2009 9;325(5945):1246–1250. 10.1126/science.1174148 19644074 PMC2867218

[4] Nica AC, Parts L, Glass D, Nisbet J, Barrett A, Sekowska M, et al The architecture of gene regulatory variation across multiple human tissues: the MuTHER study. PLoS Genet. 2011;7(2):e1002003 10.1371/journal.pgen.1002003 21304890 PMC3033383

[5] Fairfax BP, Makino S, Radhakrishnan J, Plant K, Leslie S, Dilthey A, et al Genetics of gene expression in primary immune cells identifies cell type-specific master regulators and roles of HLA alleles. Nat Genet. 2012 5;44(5):502–510. 10.1038/ng.2205 22446964 PMC3437404

[6] Stranger BE, Nica AC, Forrest MS, Dimas A, Bird CP, Beazley C, et al Population genomics of human gene expression. Nat Genet. 2007 10;39(10):1217–1224. 10.1038/ng2142 17873874 PMC2683249

[7] Veyrieras JB, Kudaravalli S, Kim SY, Dermitzakis ET, Gilad Y, Stephens M, et al High-resolution mapping of expression-QTLs yields insight into human gene regulation. PLoS Genet. 2008 10;4(10):e1000214 10.1371/journal.pgen.1000214 18846210 PMC2556086

[8] Raj T, Rothamel K, Mostafavi S, Ye C, Lee MN, Replogle JM, et al Polarization of the effects of autoimmune and neurodegenerative risk alleles in leukocytes. Science. 2014 5;344(6183):519–523. 10.1126/science.1249547 24786080 PMC4910825

[9] Wallace C, Rotival M, Cooper JD, Rice CM, Yang JH, McNeill M, et al Statistical colocalization of monocyte gene expression and genetic risk variants for type 1 diabetes. Hum Mol Genet. 2012 6;21(12):2815–2824. 10.1093/hmg/dds098 22403184 PMC3363338

[10] Selmi C, Lu Q, Humble MC. Heritability versus the role of the environment in autoimmunity. J Autoimmun. 2012 12;39(4):249–252. 10.1016/j.jaut.2012.07.011 22980030

[11] Smith EN, Kruglyak L. Gene-environment interaction in yeast gene expression. PLoS Biol. 2008 4;6(4):e83 10.1371/journal.pbio.0060083 18416601 PMC2292755

[12] Gagneur J, Stegle O, Zhu C, Jakob P, Tekkedil MM, Aiyar RS, et al Genotype-environment interactions reveal causal pathways that mediate genetic effects on phenotype. PLoS Genet. 2013;9(9):e1003803 10.1371/journal.pgen.1003803 24068968 PMC3778020

[13] Fairfax BP, Humburg P, Makino S, Naranbhai V, Wong D, Lau E, et al Innate immune activity conditions the effect of regulatory variants upon monocyte gene expression. Science. 2014 3;343(6175):1246949 10.1126/science.1246949 24604202 PMC4064786

[14] Lee MN, Ye C, Villani AC, Raj T, Li W, Eisenhaure TM, et al Common genetic variants modulate pathogen-sensing responses in human dendritic cells. Science. 2014 3;343(6175):1246980 10.1126/science.1246980 24604203 PMC4124741

[15] Ye CJ, Feng T, Kwon HK, Raj T, Wilson MT, Asinovski N, et al Intersection of population variation and autoimmunity genetics in human T cell activation. Science. 2014 9;345(6202):1254665 10.1126/science.1254665 25214635 PMC4751028

[16] Flutre T, Wen X, Pritchard J, Stephens M. A statistical framework for joint eQTL analysis in multiple tissues. PLoS Genet. 2013 5;9(5):e1003486 10.1371/journal.pgen.1003486 23671422 PMC3649995

[17] Jostins L, Ripke S, Weersma RK, Duerr RH, McGovern DP, Hui KY, et al Host-microbe interactions have shaped the genetic architecture of inflammatory bowel disease. Nature. 2012 11;491(7422):119–124. 10.1038/nature11582 23128233 PMC3491803

[18] Imielinski M, Baldassano RN, Griffiths A, Russell RK, Annese V, Dubinsky M, et al Common variants at five new loci associated with early-onset inflammatory bowel disease. Nat Genet. 2009 12;41(12):1335–1340. 10.1038/ng.489 19915574 PMC3267927

[19] Franke A, McGovern DP, Barrett JC, Wang K, Radford-Smith GL, Ahmad T, et al Genome-wide meta-analysis increases to 71 the number of confirmed Crohn’s disease susceptibility loci. Nat Genet. 2010 12;42(12):1118–1125. 10.1038/ng.717 21102463 PMC3299551

[20] Stroud CK, Nara TY, Roqueta-Rivera M, Radlowski EC, Lawrence P, Zhang Y, et al Disruption of FADS2 gene in mice impairs male reproduction and causes dermal and intestinal ulceration. J Lipid Res. 2009 9;50(9):1870–1880. 10.1194/jlr.M900039-JLR200 19351970 PMC2724775

[21] Giambartolomei C, Vukcevic D, Schadt EE, Franke L, Hingorani AD, Wallace C, et al Bayesian test for colocalisation between pairs of genetic association studies using summary statistics. PLoS Genet. 2014 5;10(5):e1004383 10.1371/journal.pgen.1004383 24830394 PMC4022491

[22] Plenge RM, Seielstad M, Padyukov L, Lee AT, Remmers EF, Ding B, et al TRAF1-C5 as a risk locus for rheumatoid arthritis–a genomewide study. N Engl J Med. 2007 9;357(12):1199–1209. 10.1056/NEJMoa073491 17804836 PMC2636867

[23] Stahl EA, Raychaudhuri S, Remmers EF, Xie G, Eyre S, Thomson BP, et al Genome-wide association study meta-analysis identifies seven new rheumatoid arthritis risk loci. Nat Genet. 2010 6;42(6):508–514. 10.1038/ng.582 20453842 PMC4243840

[24] Zhang FR, Huang W, Chen SM, Sun LD, Liu H, Li Y, et al Genomewide association study of leprosy. N Engl J Med. 2009 12;361(27):2609–2618. 10.1056/NEJMoa0903753 20018961

[25] Liu H, Irwanto A, Fu X, Yu G, Yu Y, Sun Y, et al Discovery of six new susceptibility loci and analysis of pleiotropic effects in leprosy. Nat Genet. 2015 3;47(3):267–271. 10.1038/ng.3212 25642632

[26] Zhang X, Joehanes R, Chen BH, Huan T, Ying S, Munson PJ, et al Identification of common genetic variants controlling transcript isoform variation in human whole blood. Nat Genet. 2015 4;47(4):345–352. 10.1038/ng.3220 25685889 PMC8273720

[27] Bandala-Sanchez E, Zhang Y, Reinwald S, Dromey JA, Lee BH, Qian J, et al T cell regulation mediated by interaction of soluble CD52 with the inhibitory receptor Siglec-10. Nat Immunol. 2013 7;14(7):741–748. 10.1038/ni.2610 23685786

[28] Hogquist KA, Jameson SC. The self-obsession of T cells: how TCR signaling thresholds affect fate’decisions’ and effector function. Nat Immunol. 2014 9;15(9):815–823. 10.1038/ni.2938 25137456 PMC4348363

[29] Krupashankar DS, Dogra S, Kura M, Saraswat A, Budamakuntla L, Sumathy TK, et al Efficacy and safety of itolizumab, a novel anti-CD6 monoclonal antibody, in patients with moderate to severe chronic plaque psoriasis: results of a double-blind, randomized, placebo-controlled, phase-III study. J Am Acad Dermatol. 2014 9;71(3):484–492. 10.1016/j.jaad.2014.01.897 24703722

[30] Lee JC, Espeli M, Anderson CA, Linterman MA, Pocock JM, Williams NJ, et al Human SNP links differential outcomes in inflammatory and infectious disease to a FOXO3-regulated pathway. Cell. 2013 9;155(1):57–69. 10.1016/j.cell.2013.08.034 24035192 PMC3790457

[31] Tobin DM, Vary JC, Ray JP, Walsh GS, Dunstan SJ, Bang ND, et al The lta4h locus modulates susceptibility to mycobacterial infection in zebrafish and humans. Cell. 2010 3;140(5):717–730. 10.1016/j.cell.2010.02.013 20211140 PMC2907082

[32] Lee JC, Lyons PA, McKinney EF, Sowerby JM, Carr EJ, Bredin F, et al Gene expression profiling of CD8+ T cells predicts prognosis in patients with Crohn disease and ulcerative colitis. J Clin Invest. 2011 10;121(10):4170–4179. 10.1172/JCI59255 21946256 PMC3196314

[33] McKinney EF, Lyons PA, Carr EJ, Hollis JL, Jayne DR, Willcocks LC, et al A CD8+ T cell transcription signature predicts prognosis in autoimmune disease. Nat Med. 2010 5;16(5):586–591. 10.1038/nm.2130 20400961 PMC3504359

[34] Lyons PA, Koukoulaki M, Hatton A, Doggett K, Woffendin HB, Chaudhry AN, et al Microarray analysis of human leucocyte subsets: the advantages of positive selection and rapid purification. BMC Genomics. 2007;8:64 10.1186/1471-2164-8-64 17338817 PMC1828063

[35] Purcell S, Neale B, Todd-Brown K, Thomas L, Ferreira MA, Bender D, et al PLINK: a tool set for whole-genome association and population-based linkage analyses. Am J Hum Genet. 2007 9;81(3):559–575. 10.1086/519795 17701901 PMC1950838

[36] Lyons PA, Rayner TF, Trivedi S, Holle JU, Watts RA, Jayne DR, et al Genetically distinct subsets within ANCA-associated vasculitis. N Engl J Med. 2012 7;367(3):214–223. 10.1056/NEJMoa1108735 22808956 PMC3773907

[37] Carvalho B, Bengtsson H, Speed TP, Irizarry RA. Exploration, normalization, and genotype calls of high-density oligonucleotide SNP array data. Biostatistics. 2007 4;8(2):485–499. 10.1093/biostatistics/kxl042 17189563

[38] Carvalho BS, Irizarry RA. A framework for oligonucleotide microarray preprocessing. Bioinformatics. 2010 10;26(19):2363–2367. 10.1093/bioinformatics/btq431 20688976 PMC2944196

[39] Stegle O, Parts L, Piipari M, Winn J, Durbin R. Using probabilistic estimation of expression residuals (PEER) to obtain increased power and interpretability of gene expression analyses. Nat Protoc. 2012 3;7(3):500–507. 10.1038/nprot.2011.457 22343431 PMC3398141

[40] Stegle O, Parts L, Durbin R, Winn J. A Bayesian framework to account for complex non-genetic factors in gene expression levels greatly increases power in eQTL studies. PLoS Comput Biol. 2010 5;6(5):e1000770 10.1371/journal.pcbi.1000770 20463871 PMC2865505

[41] Johnson WE, Li C, Rabinovic A. Adjusting batch effects in microarray expression data using empirical Bayes methods. Biostatistics. 2007 1;8(1):118–127. 10.1093/biostatistics/kxj037 16632515

[42] Carey VJ, Davis AR, Lawrence MF, Gentleman R, Raby BA. Data structures and algorithms for analysis of genetics of gene expression with Bioconductor: GGtools 3.x. Bioinformatics. 2009 6;25(11):1447–1448. 10.1093/bioinformatics/btp169 19349284 PMC2682516

[43] Bourgon R, Gentleman R, Huber W. Independent filtering increases detection power for high-throughput experiments. Proc Natl Acad Sci USA. 2010 5;107(21):9546–9551. 10.1073/pnas.0914005107 20460310 PMC2906865

[44] Hackstadt AJ, Hess AM. Filtering for increased power for microarray data analysis. BMC Bioinformatics. 2009;10:11 10.1186/1471-2105-10-11 19133141 PMC2661050

[45] Powell JE, Henders AK, McRae AF, Caracella A, Smith S, Wright MJ, et al The Brisbane Systems Genetics Study: genetical genomics meets complex trait genetics. PLoS ONE. 2012;7(4):e35430 10.1371/journal.pone.0035430 22563384 PMC3338511

[46] Hastie T, Tibshirani R, Friedman J, editors. The elements of statistical learning. 2nd ed Springer; 2001.

[47] Kooperberg C, Leblanc M. Increasing the power of identifying gene x gene interactions in genome-wide association studies. Genet Epidemiol. 2008 4;32(3):255–263. 10.1002/gepi.20300 18200600 PMC2955421

