# Supplementary figures and images for: Insight into Genotype-Phenotype Associations through eQTL Mapping in Multiple Cell Types in Health and Immune-Mediated Disease
Source: PLoS Genet. 2016 Mar 25;12(3):e1005908. doi: 10.1371/journal.pgen.1005908 (PMC4807835; doi:10.1371/journal.pgen.1005908)

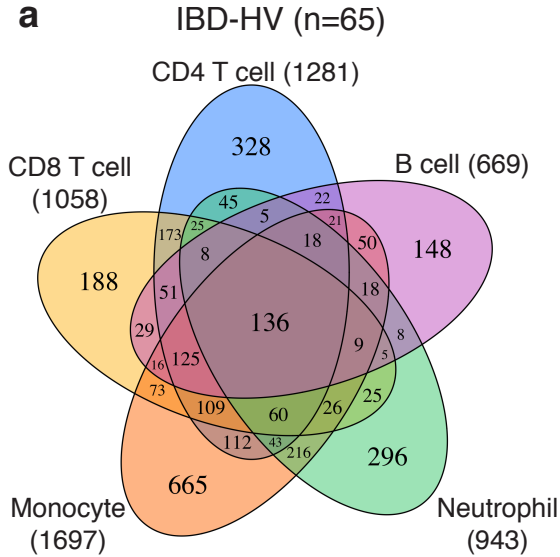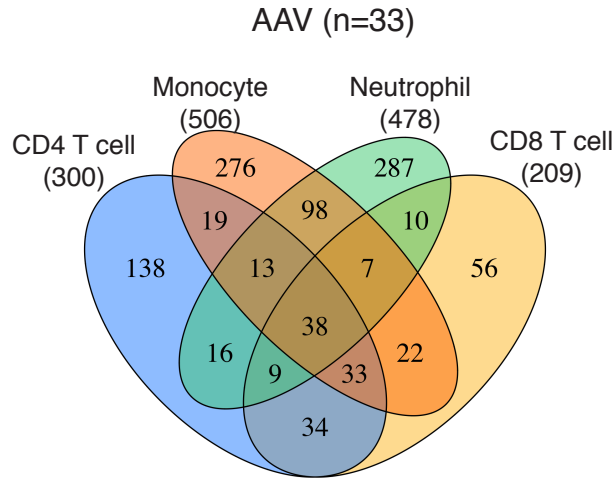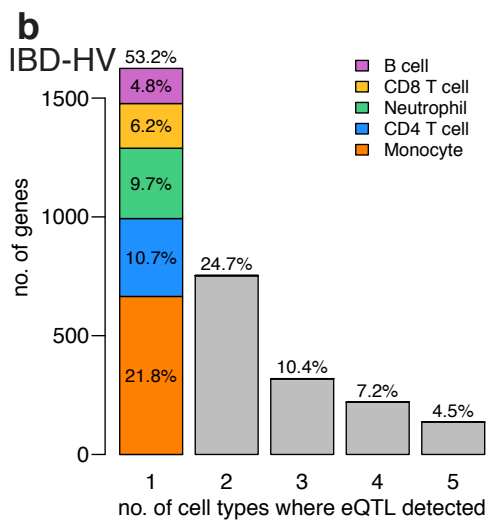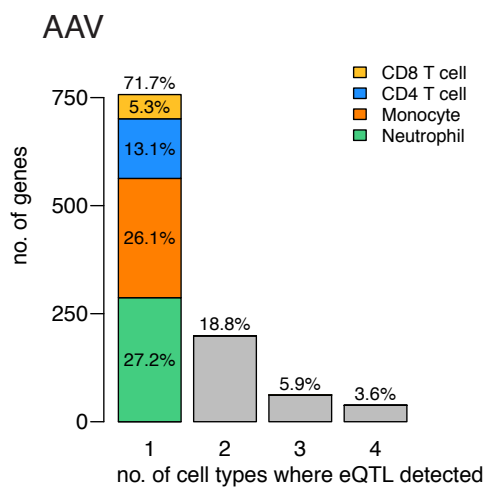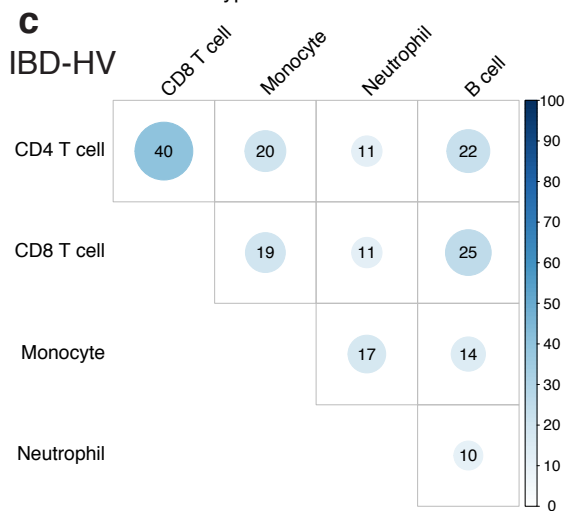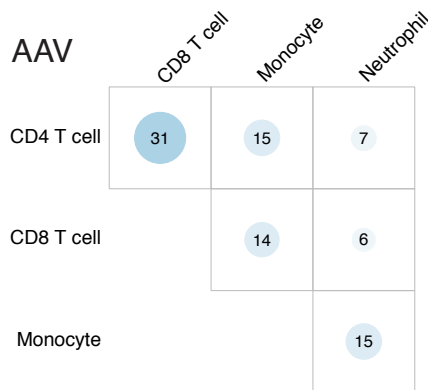

Supplement: S1 Fig — Analysis using PEER-adjusted expression data. (a) Overlap in genes with significant eQTLs (FDR <0.05) according to cell type in the joint IBD-HV analysis (n = 65), and the AAV analysis (n = 33). (b) Number of genes with an eQTL, subsetted according to the number of cell types in which the eQTL was detected. The bar for eQTLs detected in only one cell type is subdivided according to which cell type the eQTL was detected in. The denominator for the percentages shown is the total number of genes for which an eQTL was detected in at least one cell type. (c) Jaccard coefficients (as %) for similarity in eQTL profiles between all possible pairwise comparisons of cell types. (PDF) [file pgen.1005908.s001.pdf]

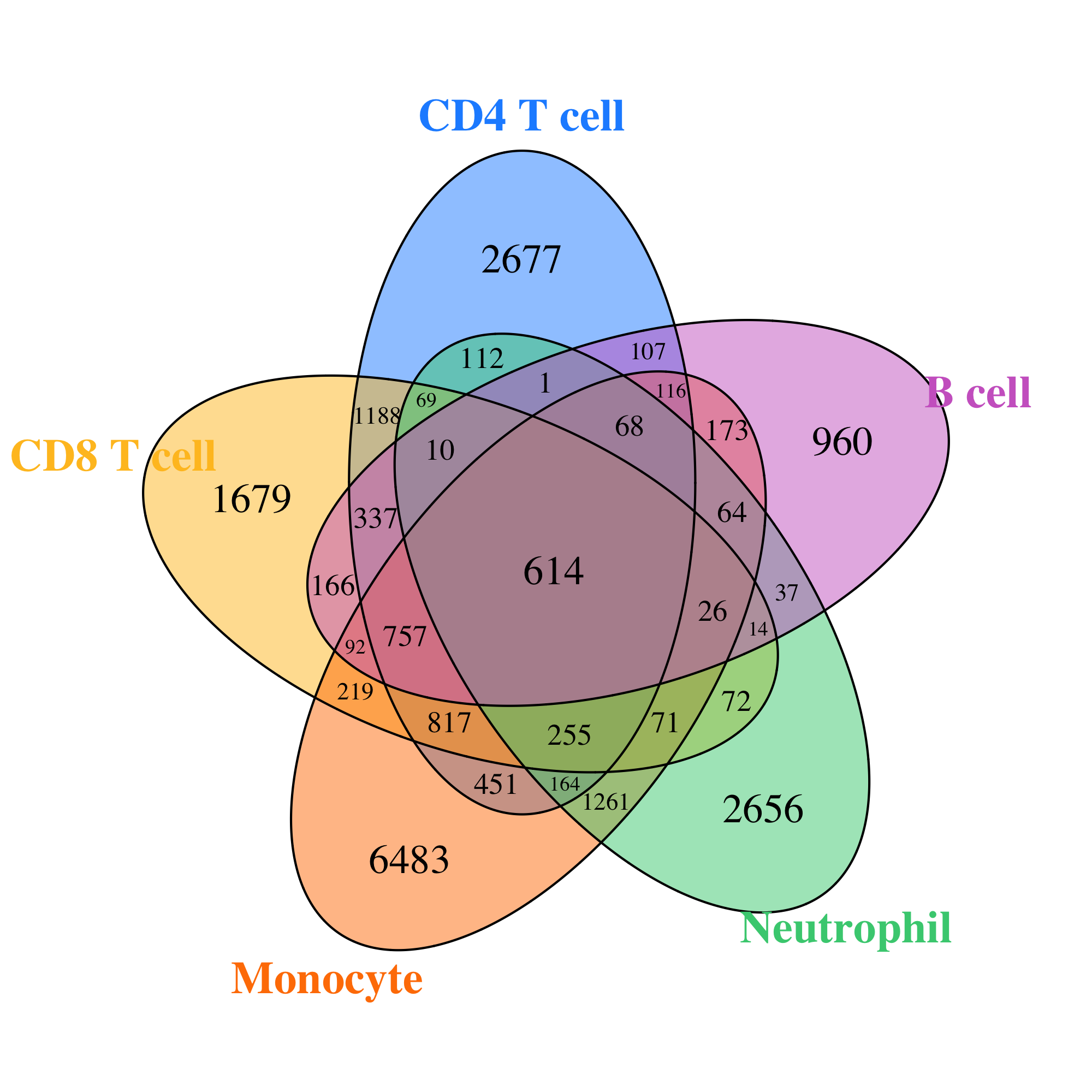

Supplement: S2 Fig — In contrast, S1 Fig shows the number of genes with eQTLs in each cell type. Analysis restricted to 65 individuals (IBD patients and HVs) with expression data available for all cell types. PEER residuals were used in the eQTL scans, with disease as a covariate. (PNG) [file pgen.1005908.s002.png]

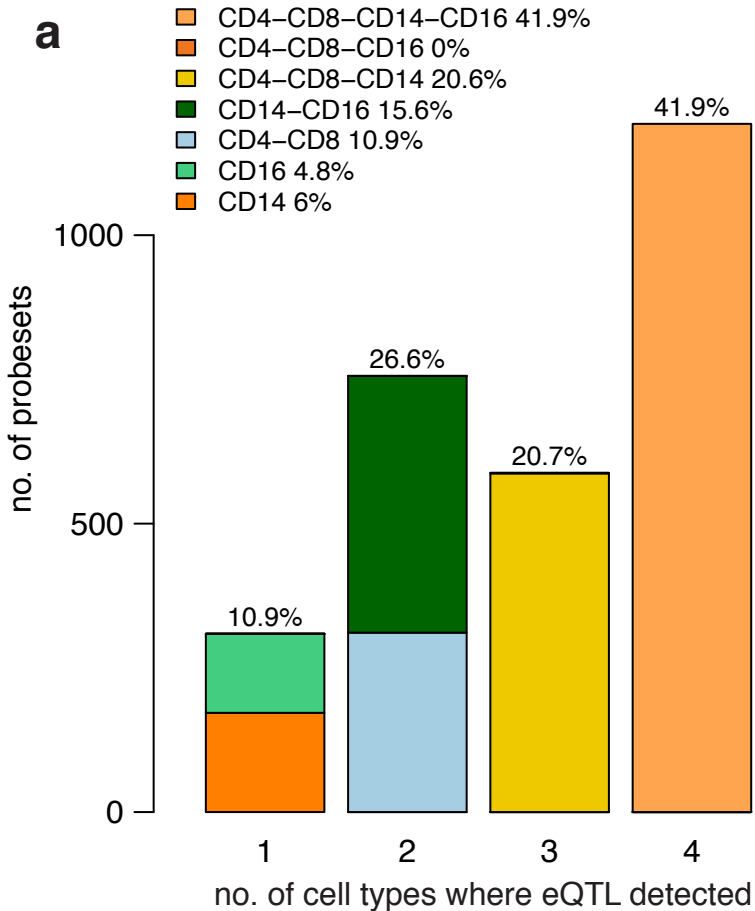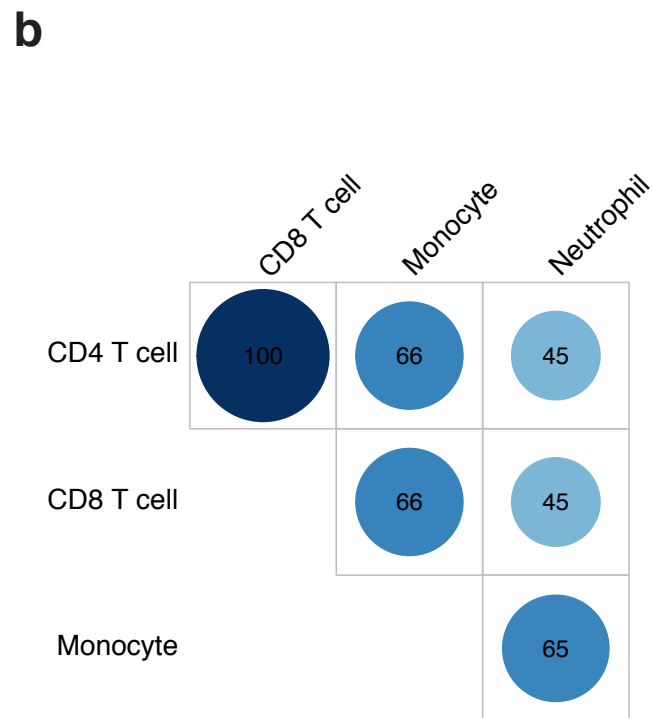

Supplement: S3 Fig — (a) Number of probesets with an eQTL, subsetted according to the number of cell types in which the eQTL was declared present. Each bar is subdivided according to which cell type the eQTL was detected in. The denominator for the percentages shown is the total number of genes for which an eQTL was detected in at least one cell type (5% Bayes FDR). Analysis using PEER-adjusted expression data. %s rounded to 1 d.p. (b) Jaccard coefficients, as %. Key: CD4 = CD4 T cells, CD8 = CD8 T cells, CD14 = monocytes, CD16 = neutrophils. (PDF) [file pgen.1005908.s003.pdf]

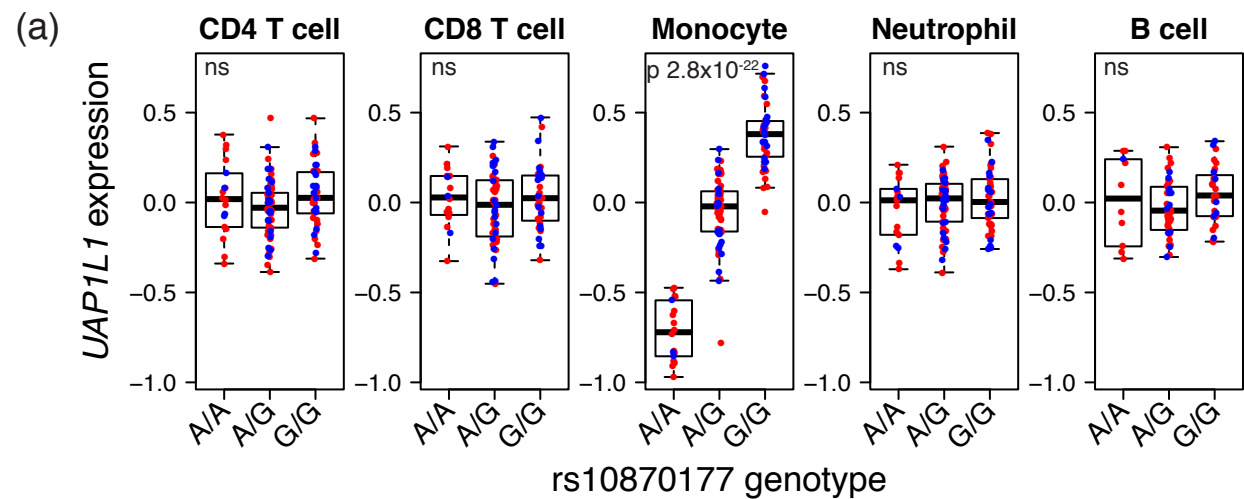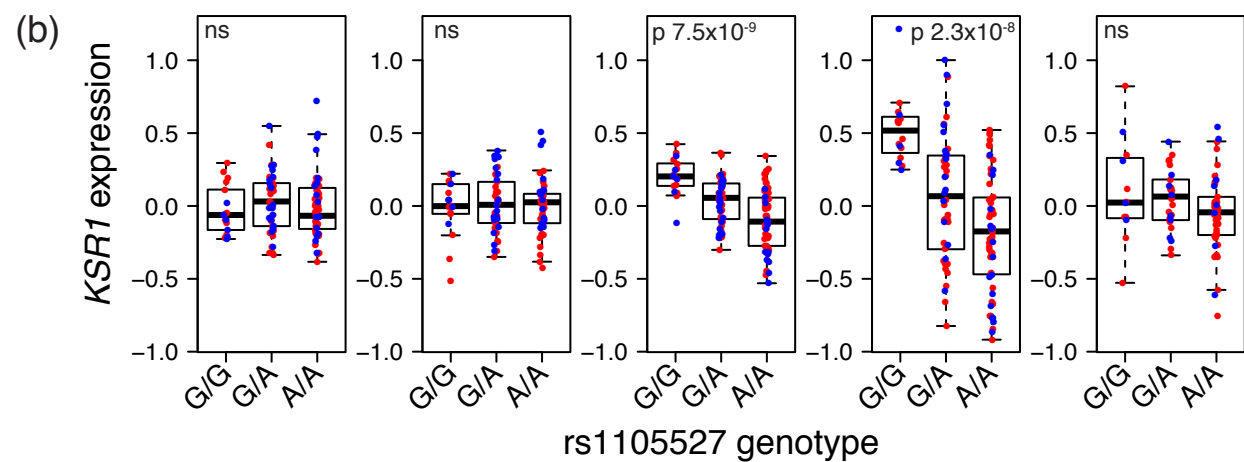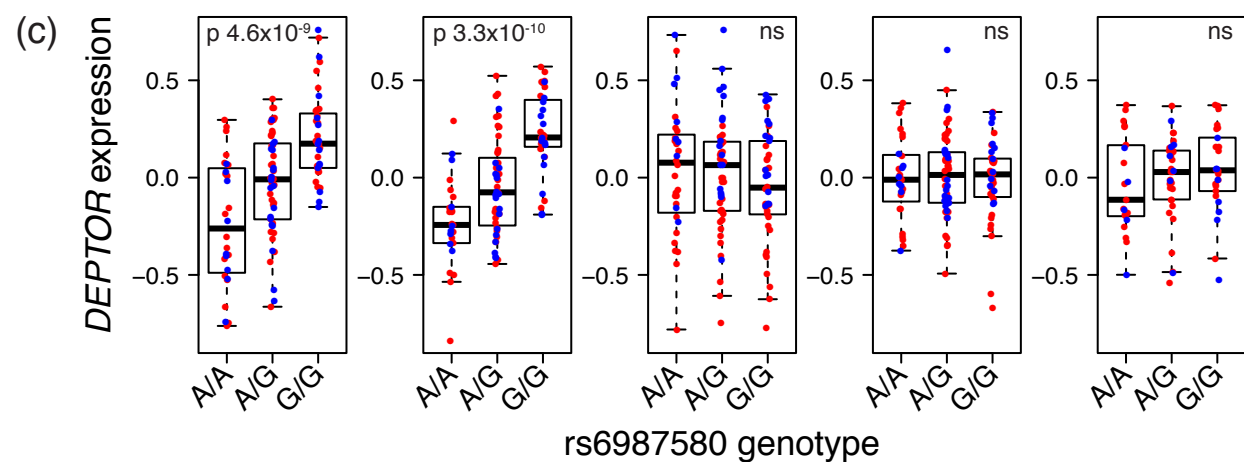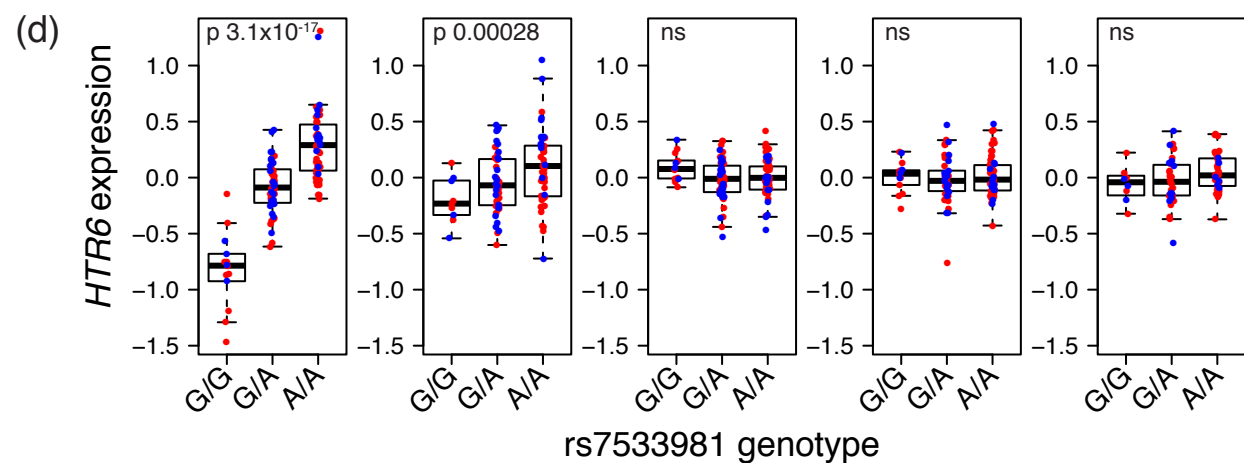

Supplement: S4 Fig — For ease of biological interpretation, in Fig 2B boxplots of the RMA-normalised expression values before adjustment with PEER were shown. Here we show expression values after adjustment of the log2 RMA-normalised expression values for batch, sex and latent factors with PEER. It should be noted that these PEER-adjusted expression values, and not the expression values in Fig 2B, were used in the eQTL scan, with disease status included as a covariate. Blue and red dots represent healthy volunteers and IBD patients respectively. Sample sizes: CD4 T cell 121, CD8 T cell 108, monocyte 124, neutrophil 121, and B cell 80. ns = not significant using a 5% FDR significance threshold. (a) Monocyte-specific, (b) myeloid-specific, and (c-d) T lymphocyte-specific eQTLs. (PDF) [file pgen.1005908.s004.pdf]

**a**

IBD-HV

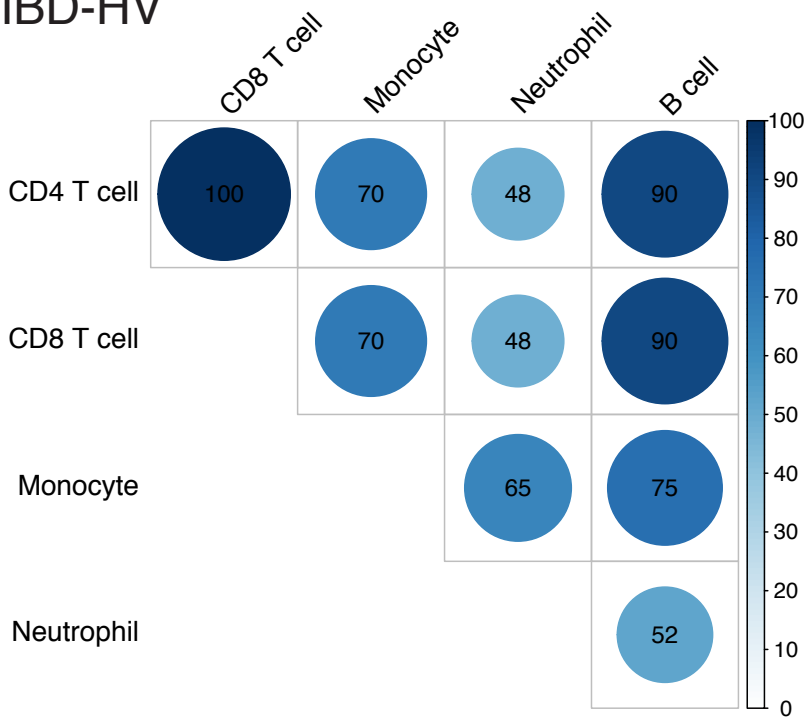

**b**

AAV

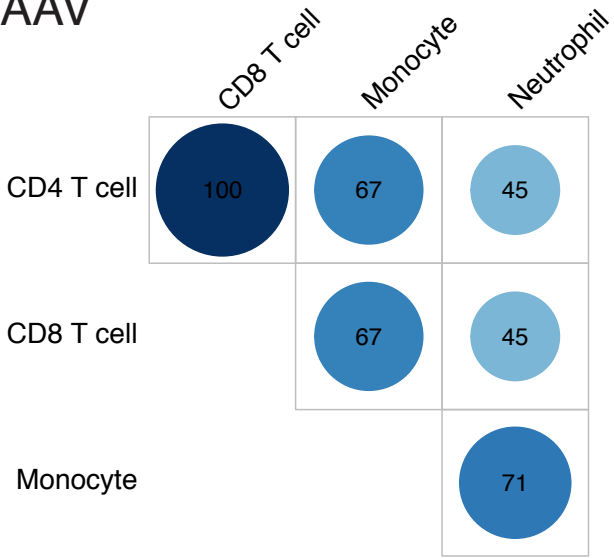

Supplement: S5 Fig — (a) IBD-HV analysis (n = 134). (b) AAV analysis (n = 46). All available samples were used, even if expression data was missing for some cell types. For Jaccard coefficients from the analysis restricted to 65 individuals with complete expression data across all 5 cell types, see S19 Fig. (PDF) [file pgen.1005908.s005.pdf]

**a**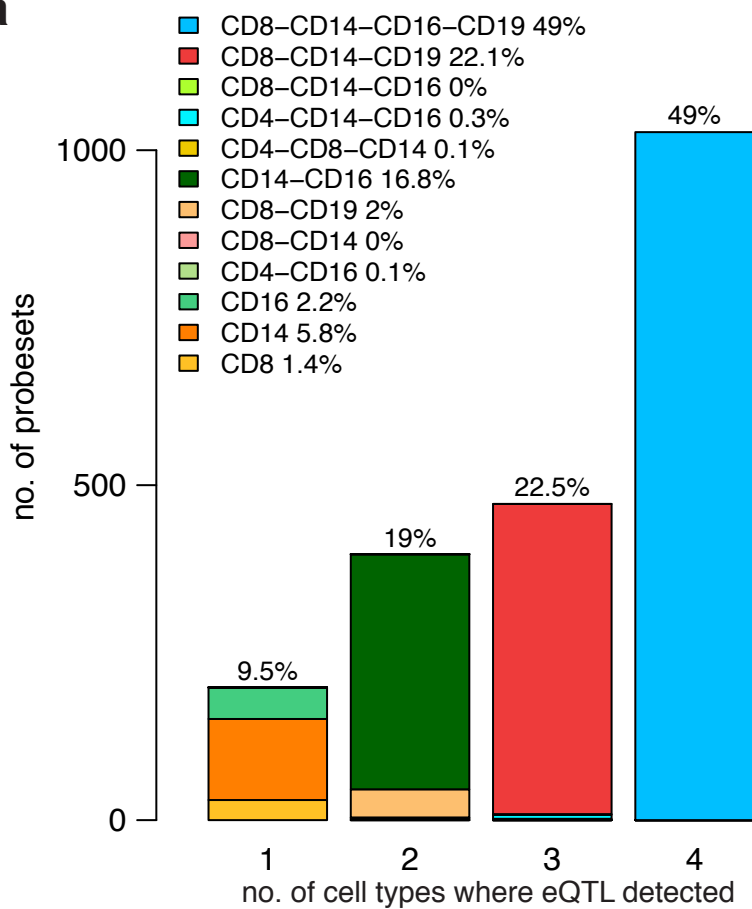**b**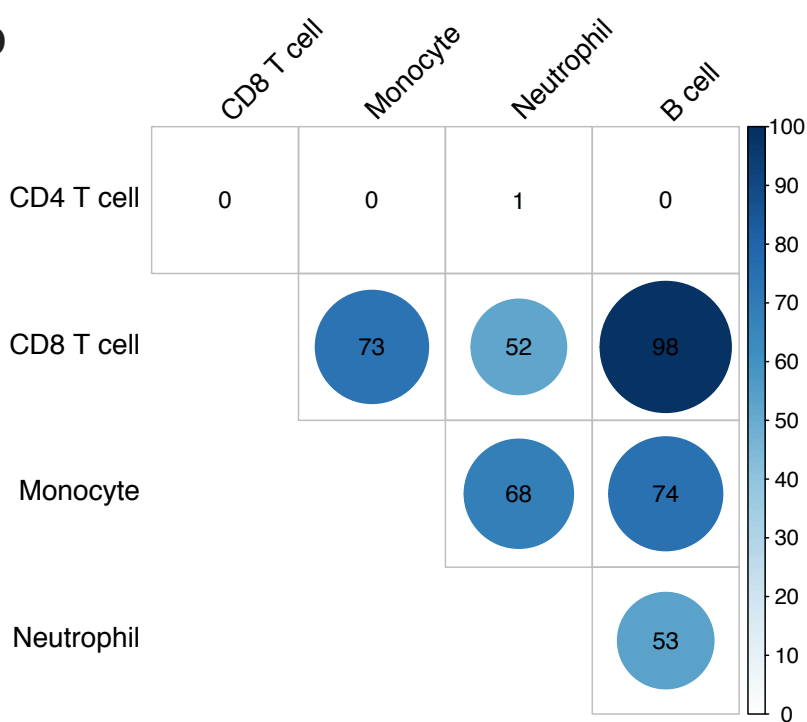

Supplement: S6 Fig — (a) Number of probesets with an eQTL, subsetted according to the number of cell types in which the eQTL was declared present (taking the cell-type configuration with the highest posterior probability). (b) Jaccard coefficients, as %. (PDF) [file pgen.1005908.s006.pdf]

**a**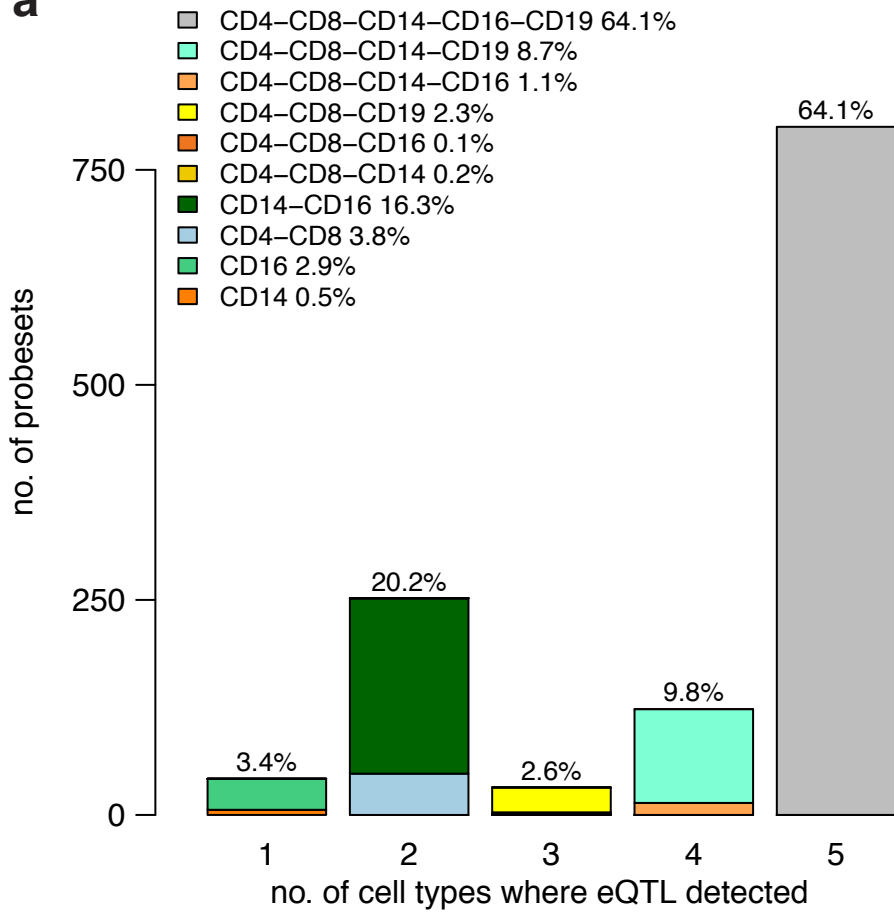**b**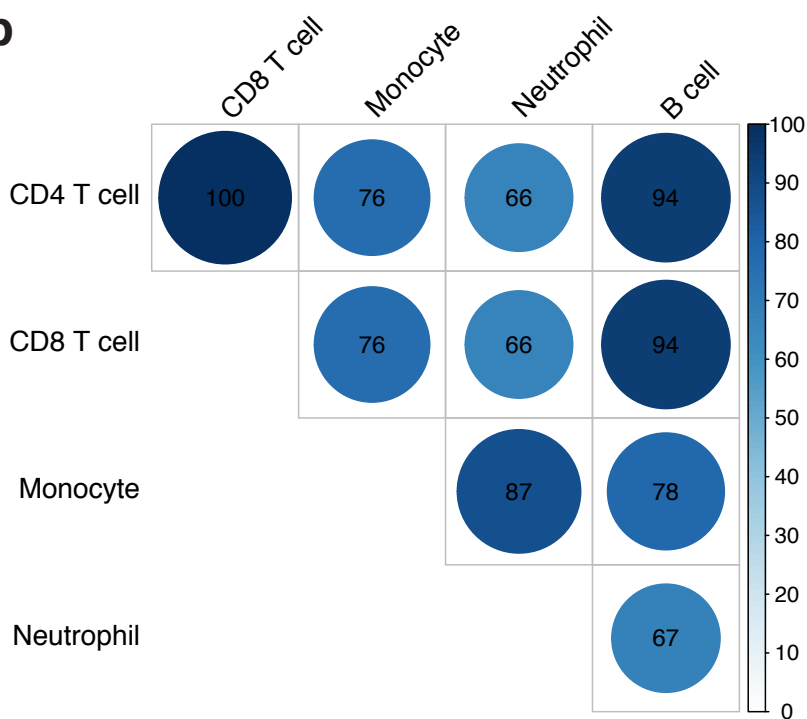

Supplement: S7 Fig — Estimates of eQTL sharing from eQTLBMA, with analysis limited to probesets expressed in all 5 cell types. This analysis was performed on the 65 individuals (IBD patients and HVs) with expression data available for all cell types. PEER residuals were used in the eQTL scans, with disease status as a covariate. (a) Number of probesets with an eQTL, subsetted according to the number of cell types in which the eQTL was declared present. Each bar is subdivided according to which cell type the eQTL was detected in. The denominator for the percentages shown is the total number of probesets for which an eQTL was detected in at least one cell type. (b) Jaccard coefficients for eQTL sharing (as %). (PDF) [file pgen.1005908.s007.pdf]

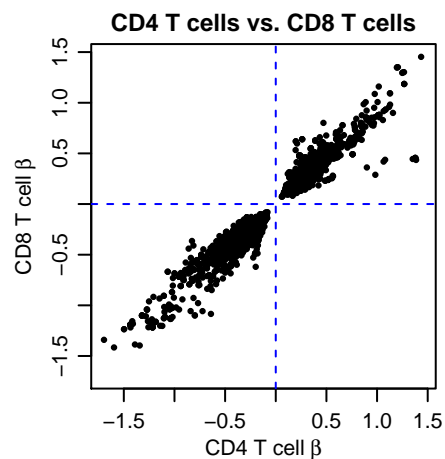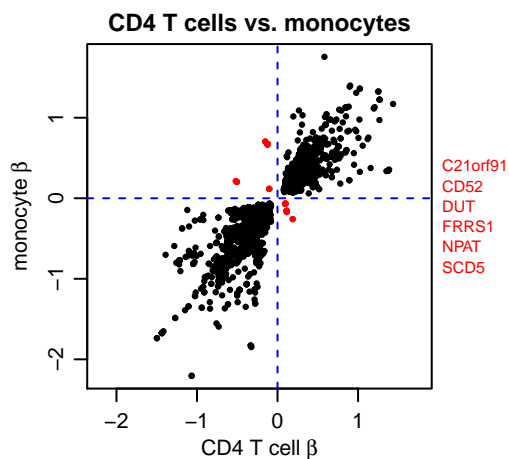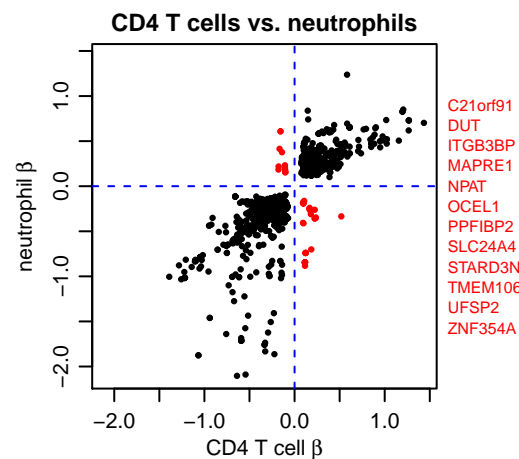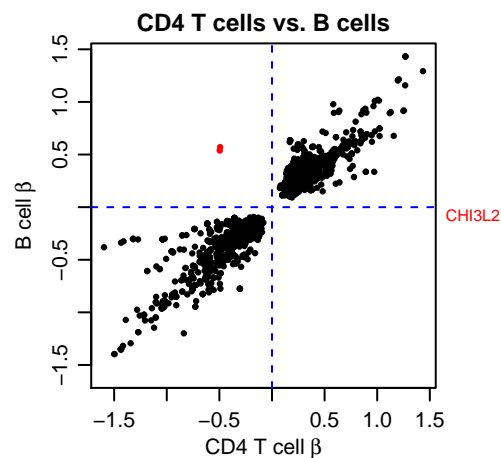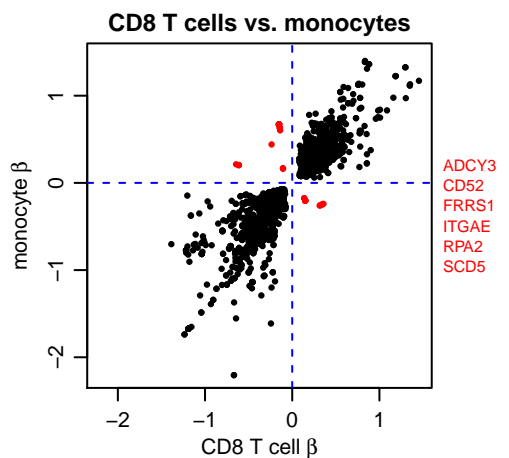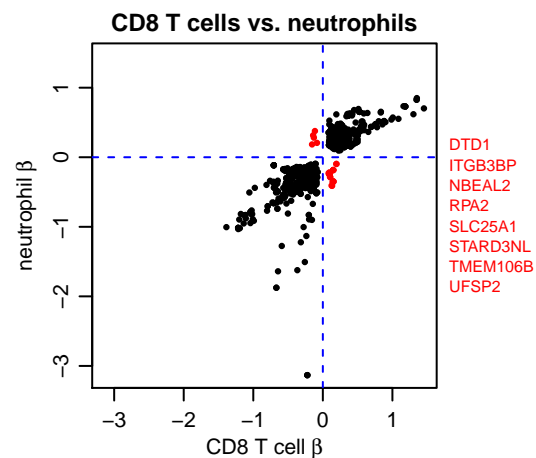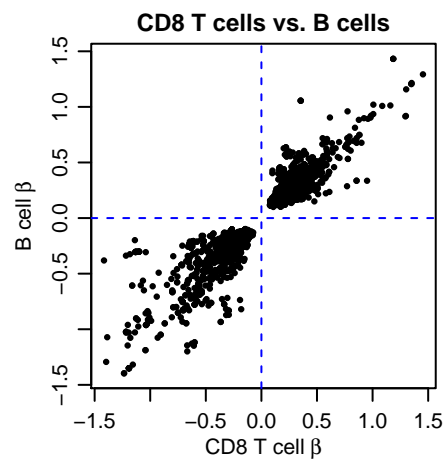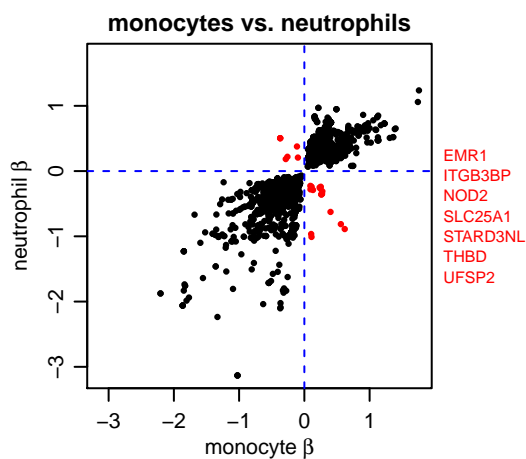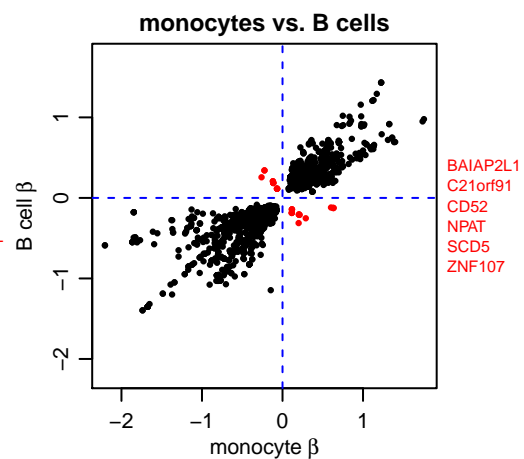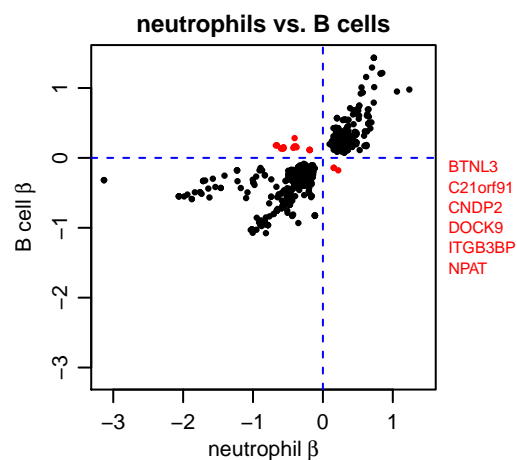

Supplement: S9 Fig — In contrast in S8 Fig we showed the n = 93 individuals for whom we had complete data across CD4 and CD8 T cells, monocytes and neutrophils. Each point represents a SNP-gene association that was statistically significant in both cell types (FDR <0.05). (PDF) [file pgen.1005908.s009.pdf]

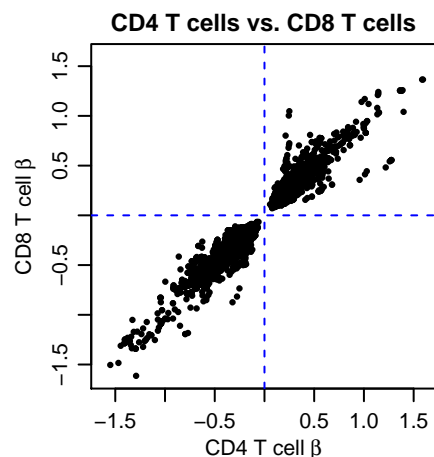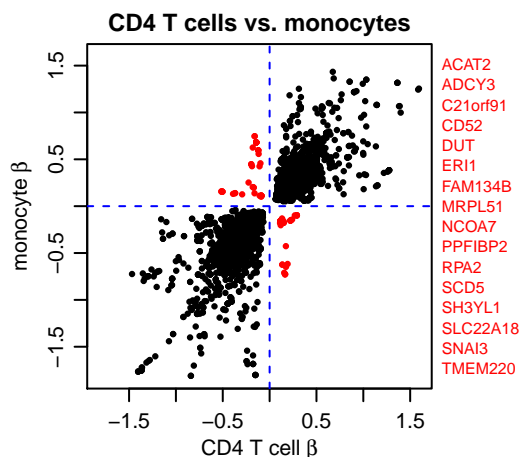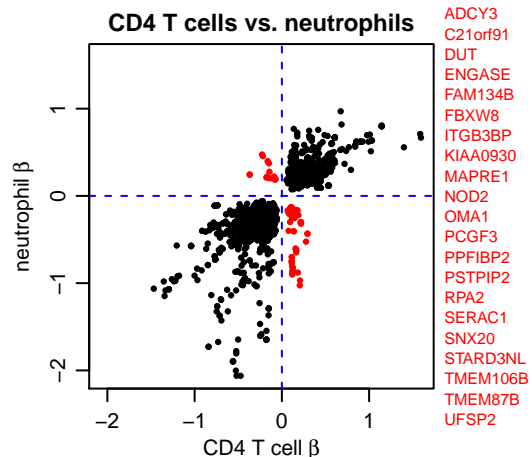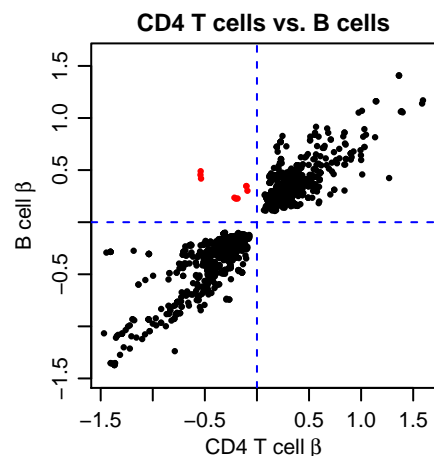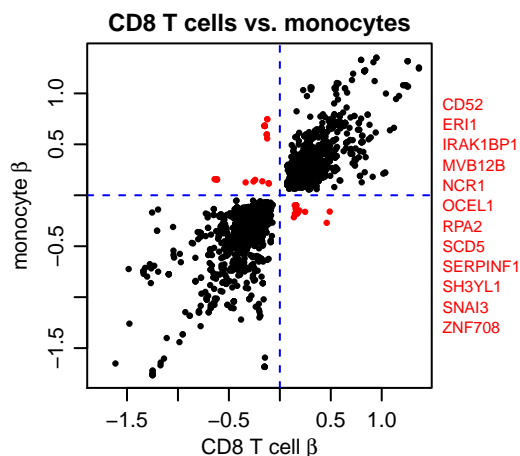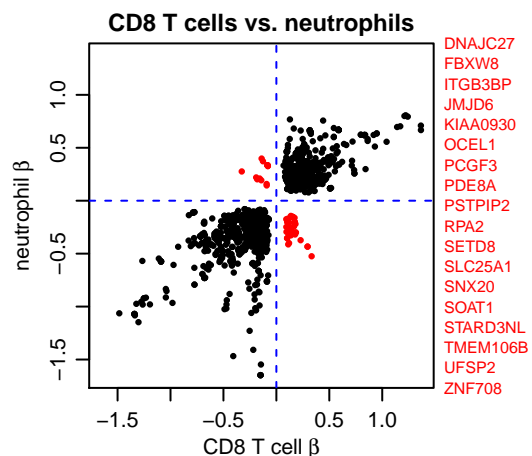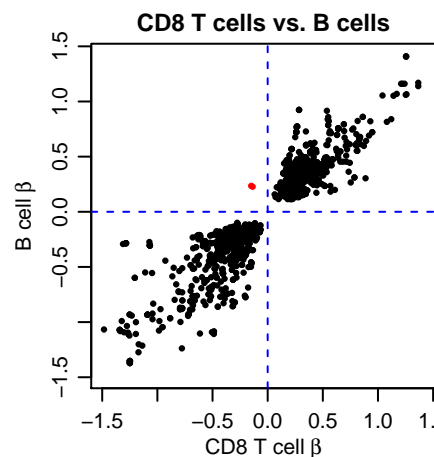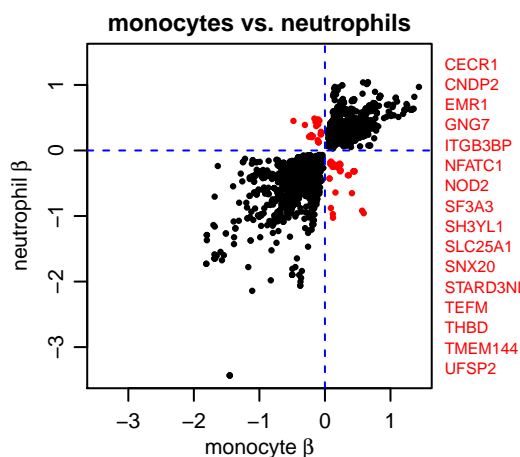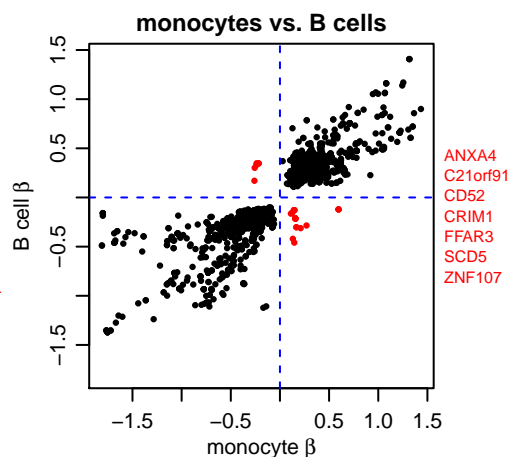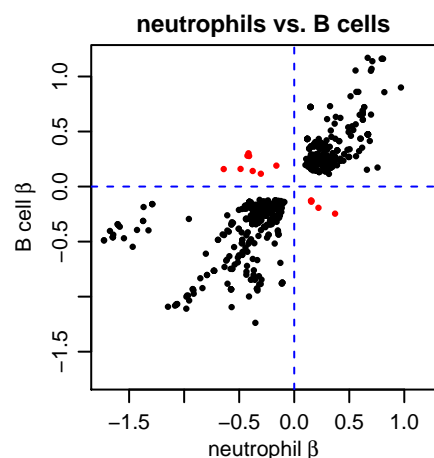

Supplement: S10 Fig — N = 79, 67, 83, 82 and 60 for CD4 T cells, CD8 T cells, monocytes, neutrophils and B cells respectively. (PDF) [file pgen.1005908.s010.pdf]

## GxD interaction

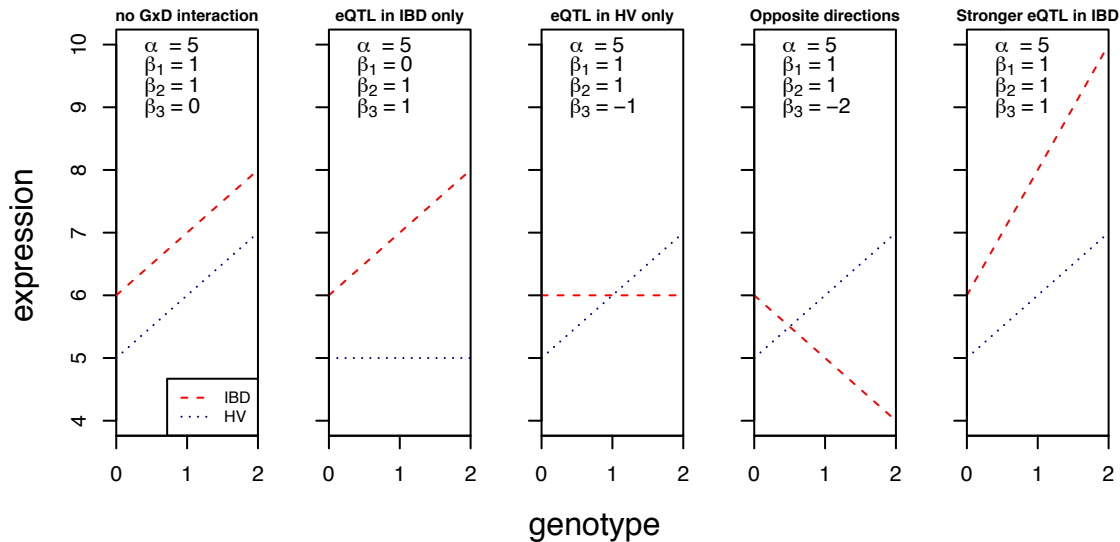

Supplement: S12 Fig — Genotype is coded 0,1 or 2 according to the number of copies of the minor allele. (PDF) [file pgen.1005908.s012.pdf]

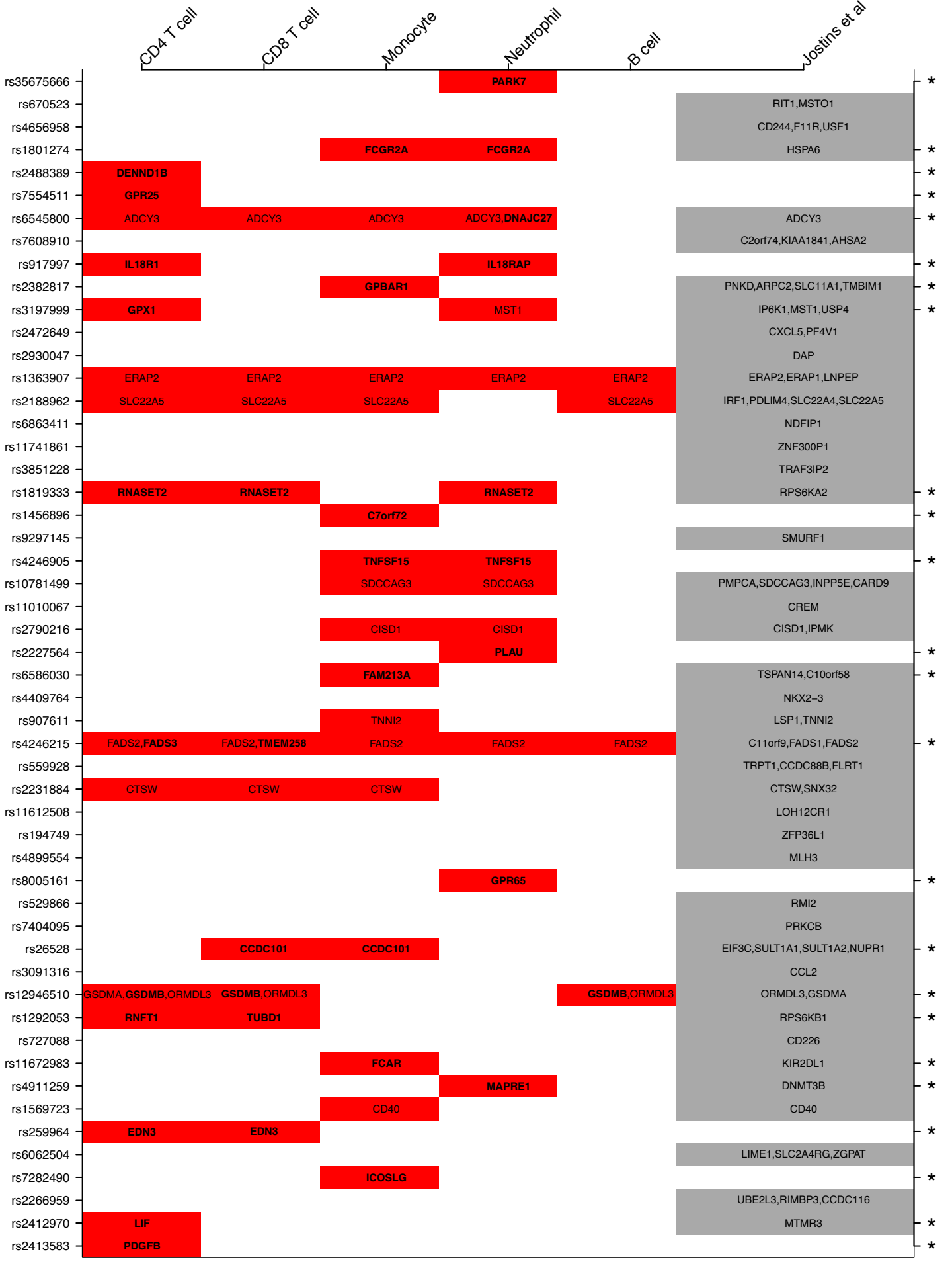

Supplement: S13 Fig — SNPs identified in the IBD meta-analysis by Jostins et al [17] as associated with IBD (both CD and UC) that are eQTLs are listed. SNPs are ordered by chromosome and position. Red blocks indicate that the SNP, or a proxy in high LD (r2 >0.8), is an eQTL in our analysis. eQTLs identified in ref. [17] are shown in grey. An asterix indicates additional information added by our analysis (either an eQTL where none was reported, or where we find the SNP is an eQTL for a different gene in at least one cell type from those previously identified; such novel candidate genes are indicated in bold). SNPs where neither we nor previous database mining [17] identify an eQTL are included in S4 Table. SNPs associated with Crohn’s disease or ulcerative colitis (but not both) that are eQTLs are shown in S14 Fig. (PDF) [file pgen.1005908.s013.pdf]

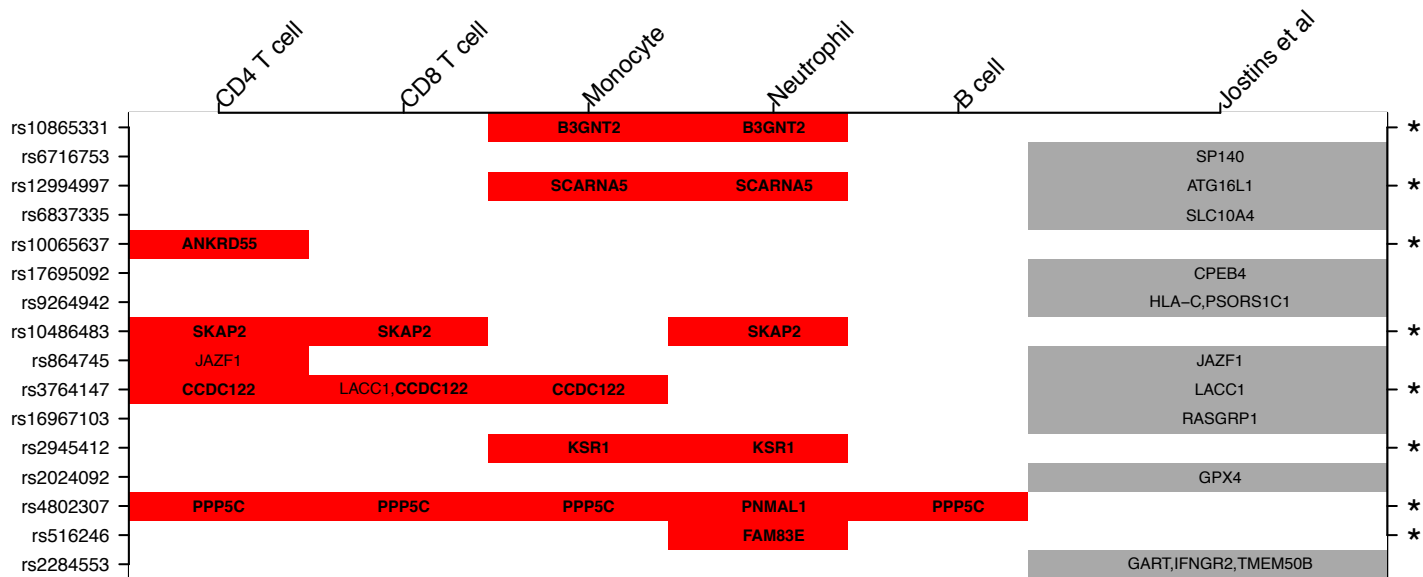

(a)

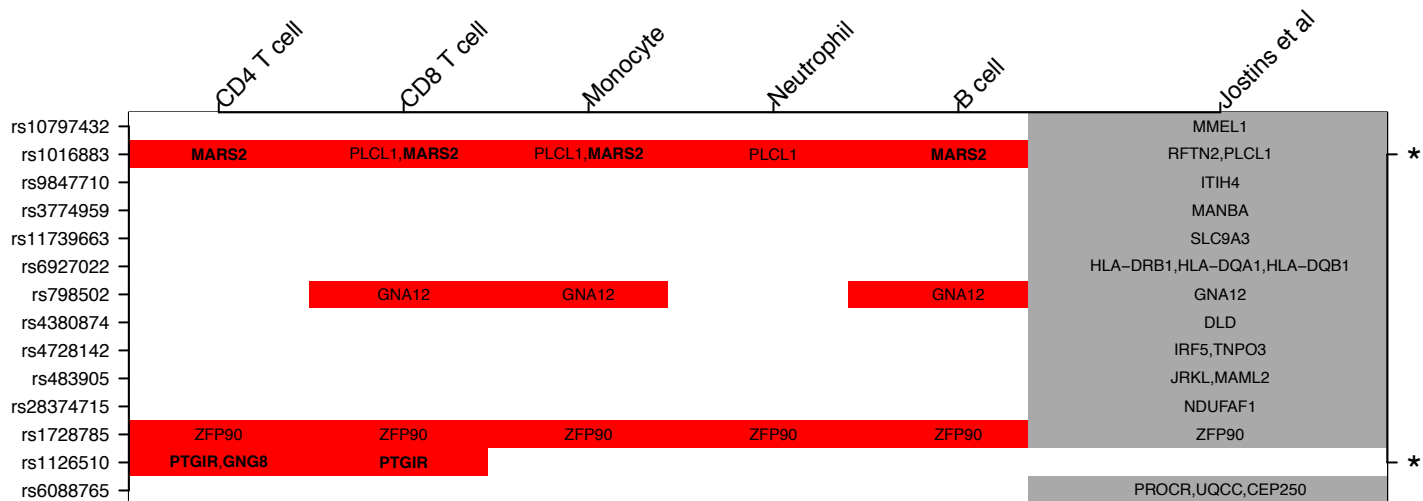

(b)

Supplement: S14 Fig — (a) CD-specific SNPs from the IBD GWAS meta-analysis by Jostins et al [17] which are eQTLs. (b) UC-specific SNPs which are eQTLs. SNPs associated with both CD and UC are shown in S13 Fig. SNPs were taken from Supplementary Table 2 of the IBD GWAS meta-analysis by Jostins et al [17], and are ordered by chromosome and position. Red blocks indicate that the SNP or a proxy in high LD (r2 >0.8) is an eQTL in our analysis (using PEER-adjusted expression data and all available IBD and HV samples, i.e. not restricted to the individuals for whom genotype and expression data was available in all cell types). The gene(s) whose expression is associated with that SNP are printed on the plot. eQTLs identified through database mining in ref. [17] are shown in grey. Any SNP highlighted with an asterix indicates additional information added by our analysis (either an eQTL where none was reported in ref. [17], or where we find the SNP is an eQTL for a different gene in at least one cell type from those previously identified; such novel candidate genes are indicated in bold). SNPs where neither we nor ref. [17] identify an eQTL are not shown here, but are included in S4 Table. (PDF) [file pgen.1005908.s014.pdf]

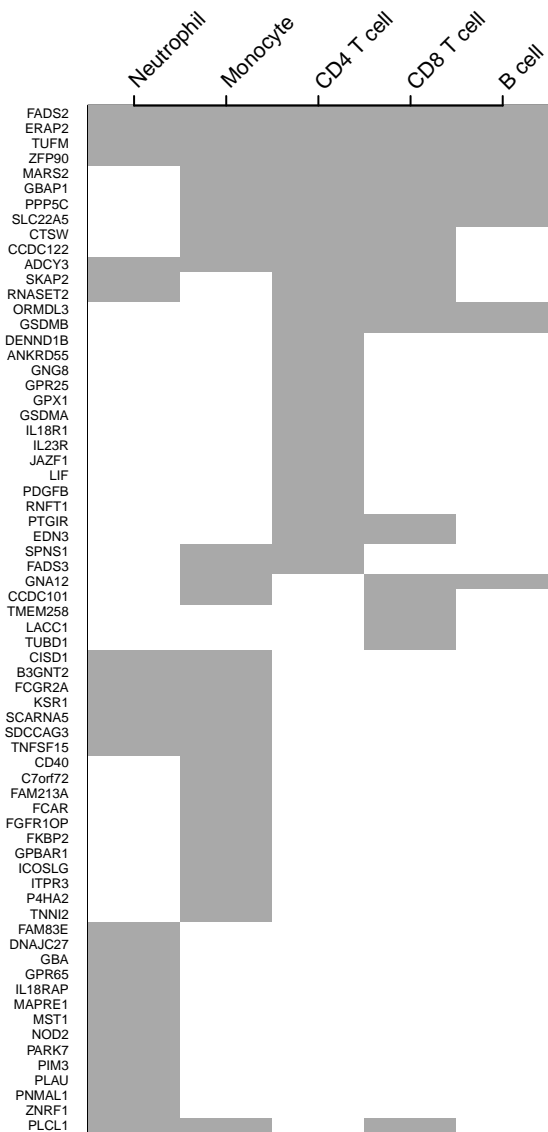

Supplement: S15 Fig — We took eQTL SNPs identified in each cell type in the joint HV-IBD analysis (using PEER-adjusted expression data, FDR <0.05), and intersected them with the list of IBD-associated SNPs from the NHGRI GWAS catalogue and proxies in high LD (r2 >0.8). We thus identified SNPs which are both eQTLs and are associated with IBD. Here we show the genes whose expression is associated with these SNPs in each cell type. Grey shading indicates the gene has a cis eQTL which is an IBD hit or one of its proxies. The ordering of the cell types and genes is the result of hierarchical clustering. Here we use all eSNPs passing the 5% FDR significance threshold as the eQTL SNPs. In contrast, S16 Fig uses only the best eQTL per gene. (PDF) [file pgen.1005908.s015.pdf]

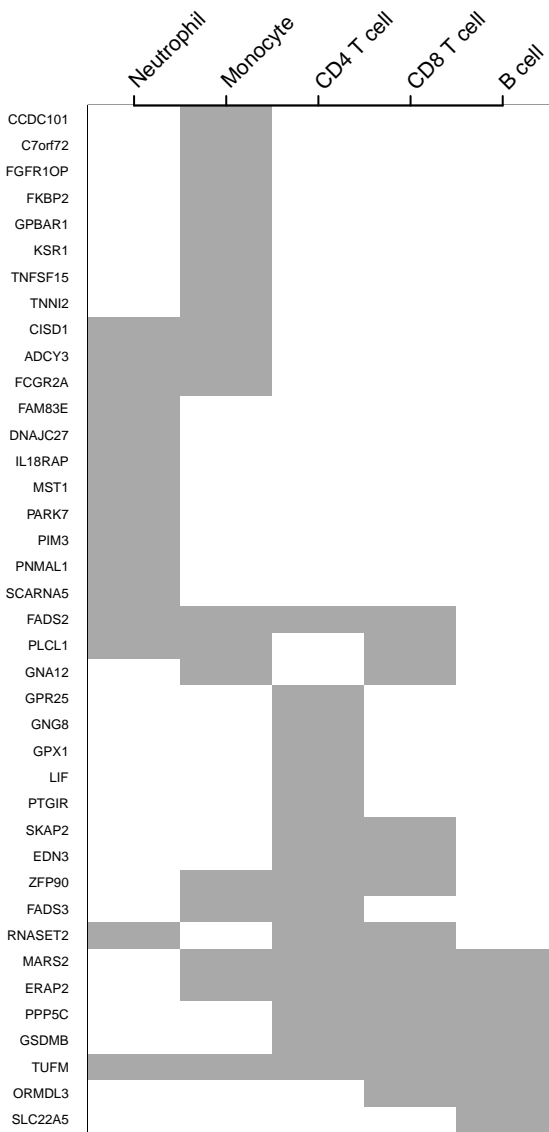

Supplement: S16 Fig — From the list of significant eQTL SNPs (FDR <0.05, joint HV-IBD analysis, PEER-adjusted expression data), we took only the best cis eQTL SNP per gene (in contrast with S15 Fig where we used all significantly associated SNPs). We intersected these SNPs with the list of IBD-associated SNPs from the NHGRI GWAS catalogue and proxies in high LD (r2 >0.8). Grey shading indicates the gene’s best cis eQTL is an IBD GWAS hit or one of its proxies. The ordering of the cell types and genes is the result of hierarchical clustering. (PDF) [file pgen.1005908.s016.pdf]

**a**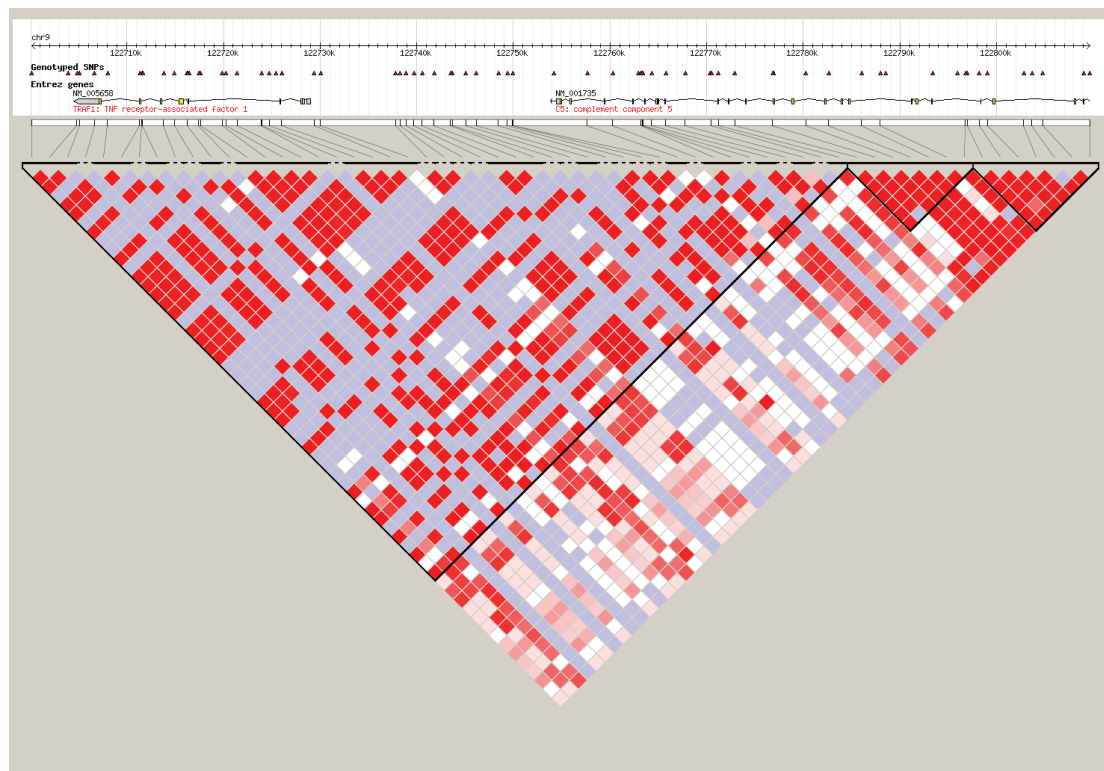**b**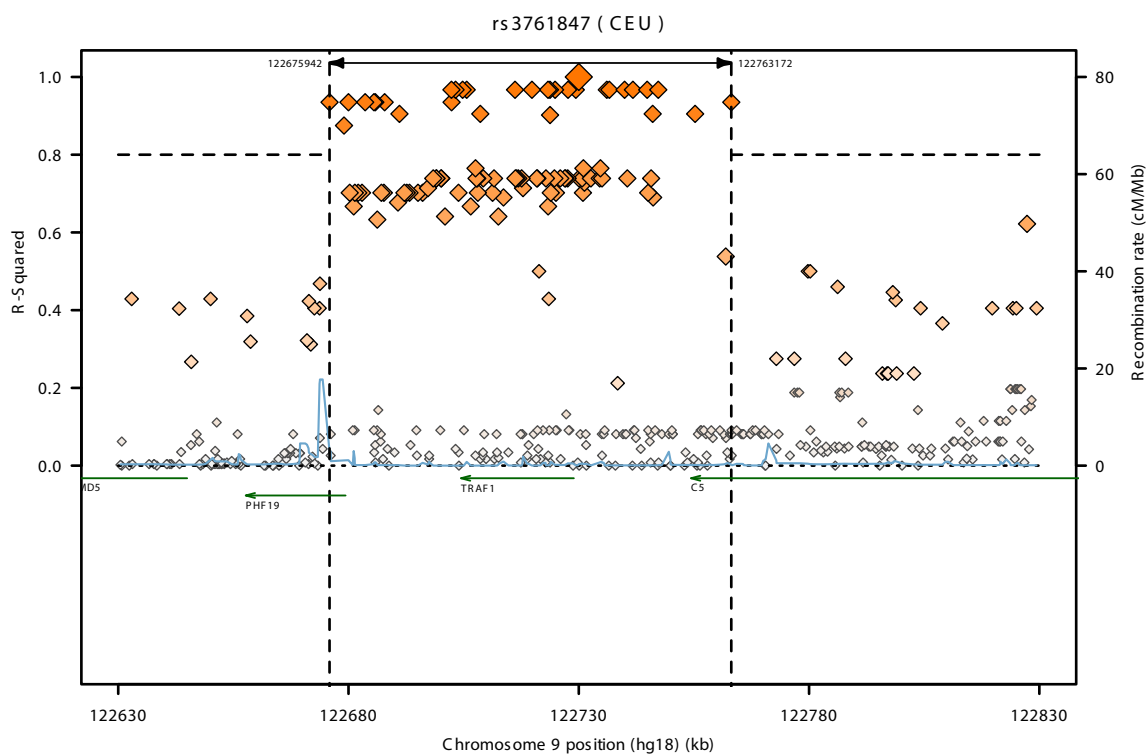

Supplement: S17 Fig — (a) LD structure visualised using Haploview. (b) r2 to rs3761847 in CEU population (plot made using SNAP https://www.broadinstitute.org/mpg/snap/). Positions shown are hg18 genome build. (PDF) [file pgen.1005908.s017.pdf]

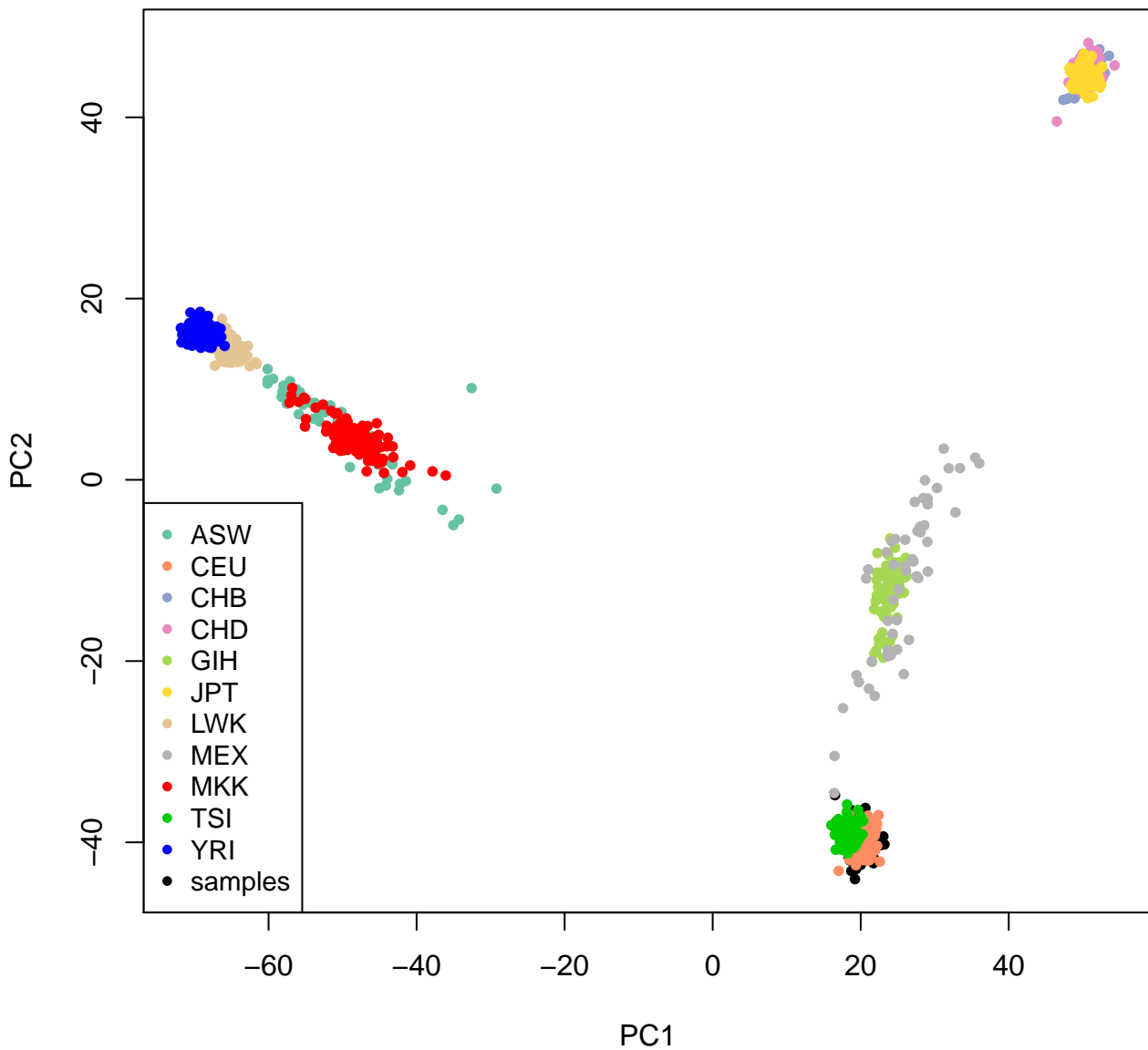

Supplement: S18 Fig — Each point represents an individual. Our samples are coloured black. Ethnicity of HapMap individuals is indicated by the legend in the bottom left corner. (PDF) [file pgen.1005908.s018.pdf]

**a**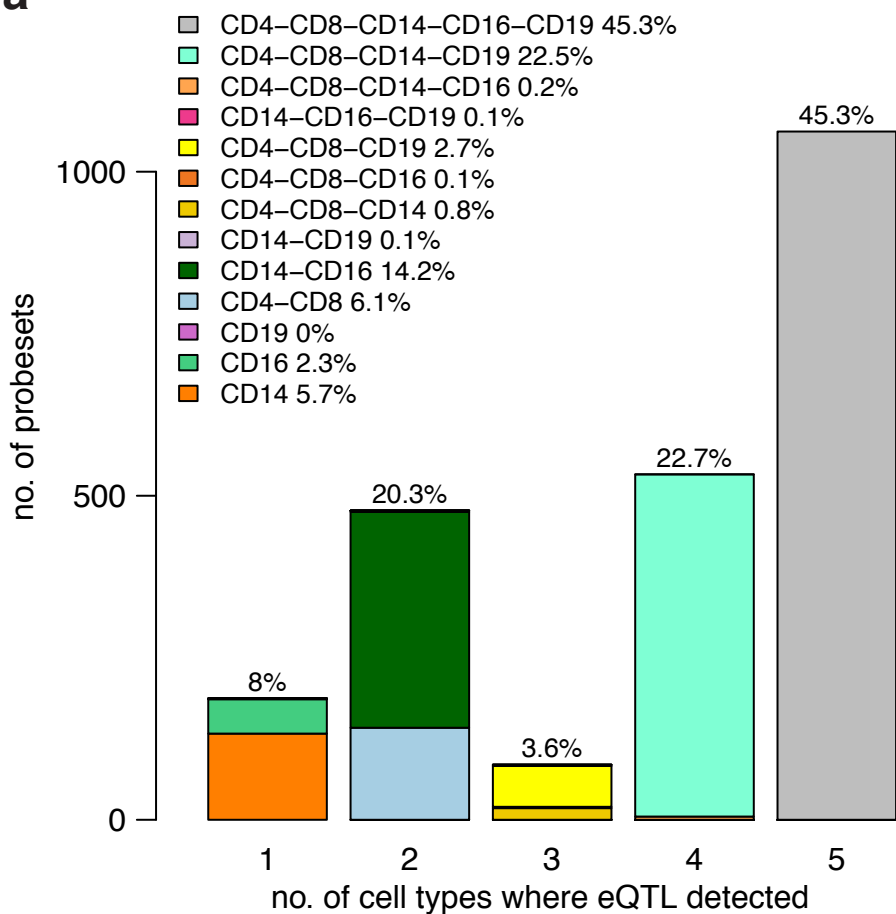**b**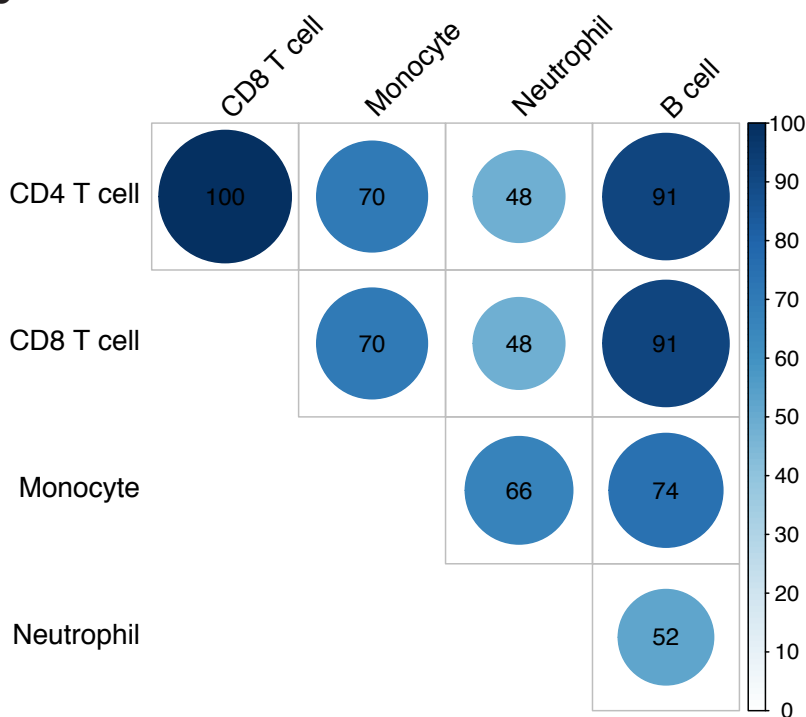

Supplement: S19 Fig — (a) eQTLs divided according to their ‘best’ configuration across cell types. (b) Jaccard coefficients, as %. (PDF) [file pgen.1005908.s019.pdf]

**CD14**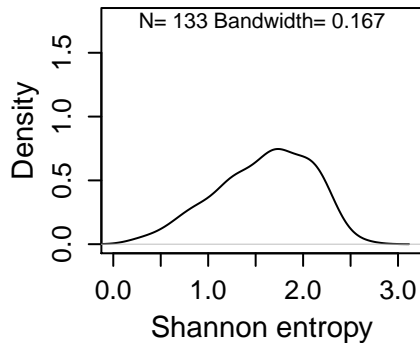**CD16**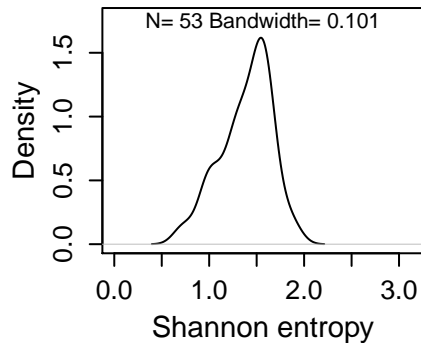**CD4-CD8**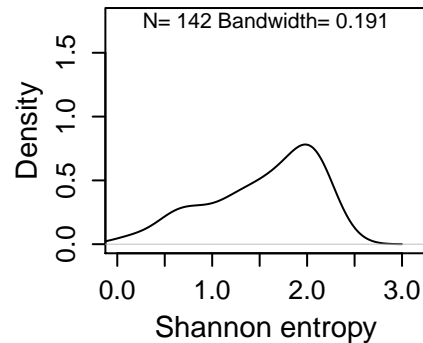**CD14-CD16**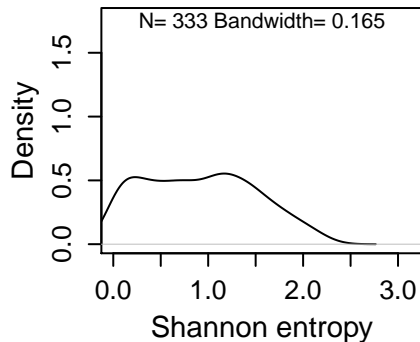**CD4-CD8-CD14**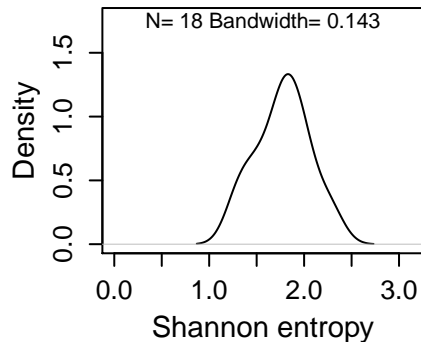**CD4-CD8-CD19**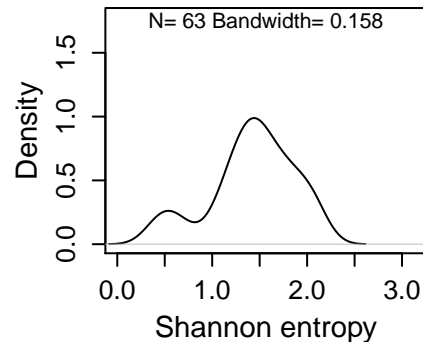**CD4-CD8-CD14-CD19**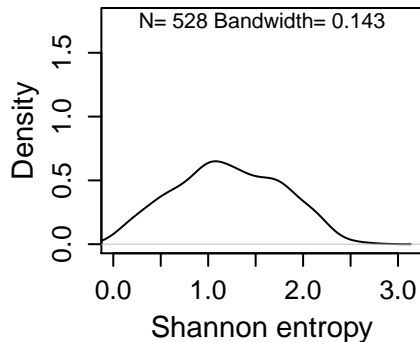**CD4-CD8-CD14-CD16-CD19**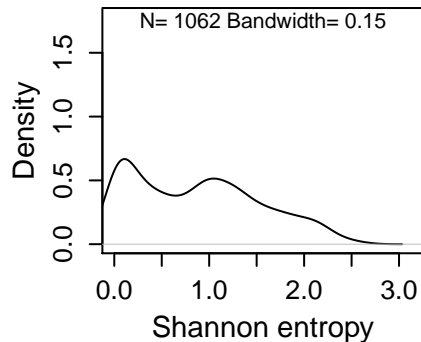**All configurations**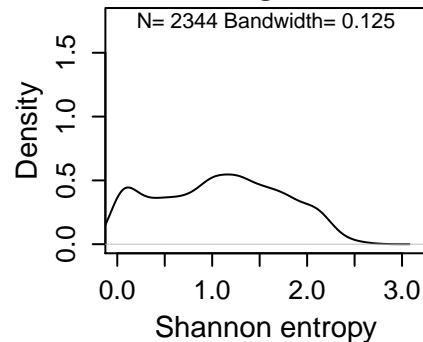

Supplement: S20 Fig — The Shannon entropy is a measure of uncertainty, where a higher entropy indicates more uncertainty. Shannon entropies were calculated on the configuration posterior probabilities for the best SNP for each gene with a significant eQTL (5% Bayes FDR). Results from joint modelling across 5 cell types using eQTLBMA on the 65 individuals (IBD-HV dataset) with complete expression data across all 5 cell types. CD4 = CD4 T cells, CD8 = CD8 T cells, CD14 = monocytes, CD16 = neutrophils, CD19 = B cells. (PDF) [file pgen.1005908.s020.pdf]
