# Supplementary material for: Insight into Genotype-Phenotype Associations through eQTL Mapping in Multiple Cell Types in Health and Immune-Mediated Disease
Source: PLoS Genet. 2016 Mar 25;12(3):e1005908. doi: 10.1371/journal.pgen.1005908 (PMC4807835; doi:10.1371/journal.pgen.1005908)
Supplement: S8 Fig — Each point represents a SNP-gene association that was statistically significant in both cell types (FDR <0.05). For each SNP-gene association the axes show the estimated effect size (beta) in each cell type. eQTLs with opposing directions of effect between cell types have a positive effect size in one cell type but a negative one in the other (points in the upper left and lower right quadrants). The names of genes with such eQTLs are printed in red. As a consequence of LD, there are sometimes multiple eSNPs significantly associated with one gene, so there are more points in the upper left and lower right quadrants than gene names printed on the plot. (PDF) [file pgen.1005908.s008.pdf]

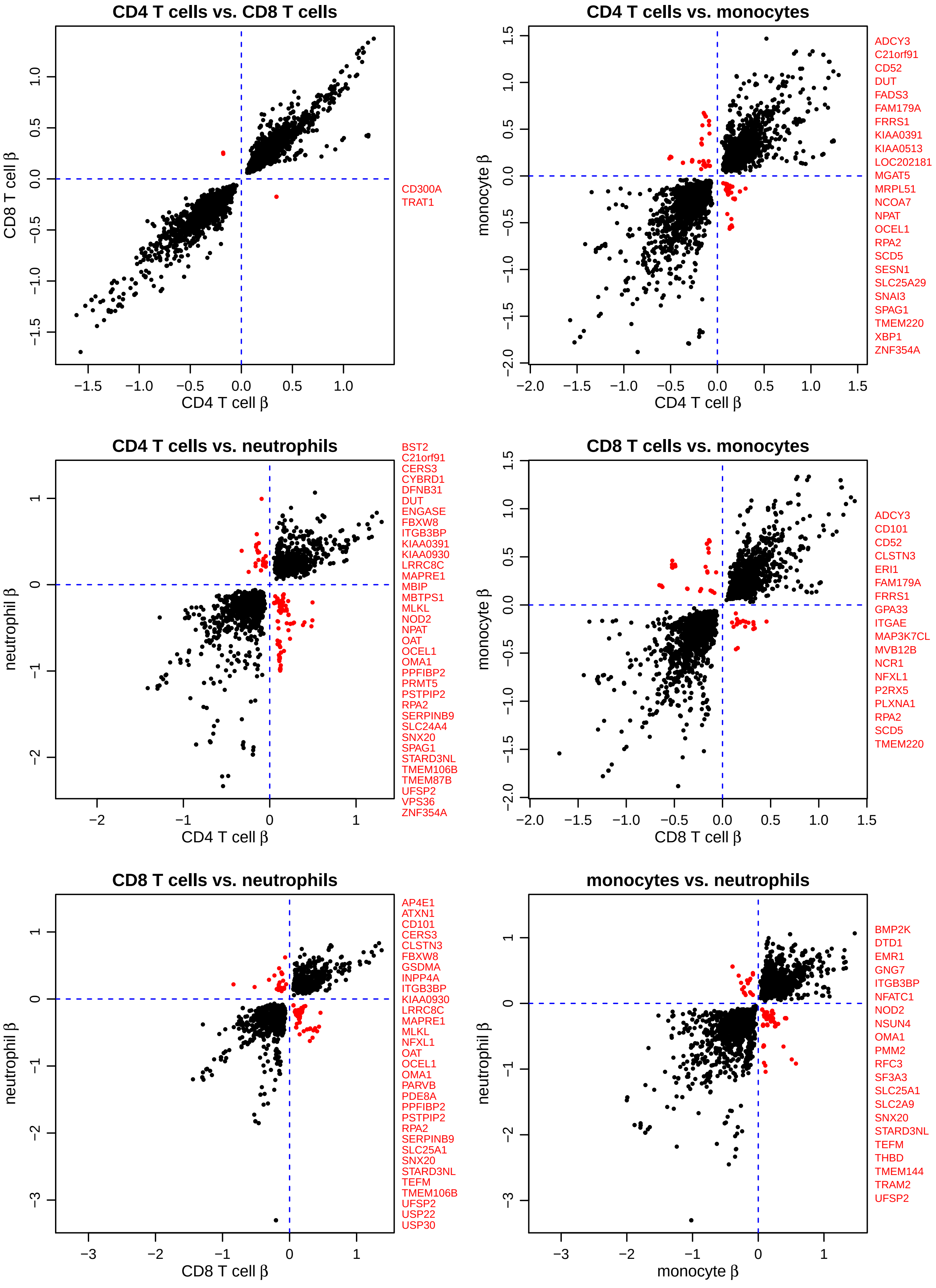
CD4 T cells vs. CD8 T cells
CD8 T cell β
CD4 T cell β
CD300A
TRAT1
CD4 T cells vs. monocytes
monocyte β
CD4 T cell β
ADCY3
C21orf91
CD52
DUT
FADS3
FAM179A
FRRS1
KIAA0391
KIAA0513
LOC202181
MGAT5
MRPL51
NCOA7
NPAT
OCEL1
RPA2
SCD5
SESN1
SLC25A29
SNAI3
SPAG1
TMEM220
XBP1
ZNF354A
CD4 T cells vs. neutrophils
neutrophil β
CD4 T cell β
BST2
C21orf91
CERS3
CYBRD1
DFNB31
DUT
ENGASE
FBXW8
ITGB3BP
KIAA0391
KIAA0930
LRRC8C
MAPRE1
MBIP
MBTPS1
MLKL
NOD2
NPAT
OAT
OCEL1
OMA1
PPFIBP2
PRMT5
PSTPIP2
RPA2
SERPINB9
SLC24A4
SNX20
SPAG1
STARD3NL
TMEM106B
TMEM87B
UFSP2
VPS36
ZNF354A
CD8 T cells vs. monocytes
monocyte β
CD8 T cell β
ADCY3
CD101
CD52
CLSTN3
ERI1
FAM179A
FRRS1
GPA33
ITGAE
MAP3K7CL
MVB12B
NCR1
NFXL1
P2RX5
PLXNA1
RPA2
SCD5
TMEM220
CD8 T cells vs. neutrophils
neutrophil β
CD8 T cell β
AP4E1
ATXN1
CD101
CERS3
CLSTN3
FBXW8
GSDMA
INPP4A
ITGB3BP
KIAA0930
LRRC8C
MAPRE1
MLKL
NFXL1
OAT
OCEL1
OMA1
PARVB
PDE8A
PPFIBP2
PSTPIP2
RPA2
SERPINB9
SLC25A1
SNX20
STARD3NL
TEFM
TMEM106B
UFSP2
USP22
USP30
monocytes vs. neutrophils
neutrophil β
monocyte β
BMP2K
DTD1
EMR1
GNG7
ITGB3BP
NFATC1
NOD2
NSUN4
OMA1
PMM2
RFC3
SF3A3
SLC25A1
SLC2A9
SNX20
STARD3NL
TEFM
THBD
TMEM144
TRAM2
UFSP2
