# Supplementary material for: Insight into Genotype-Phenotype Associations through eQTL Mapping in Multiple Cell Types in Health and Immune-Mediated Disease
Source: PLoS Genet. 2016 Mar 25;12(3):e1005908. doi: 10.1371/journal.pgen.1005908 (PMC4807835; doi:10.1371/journal.pgen.1005908)
Supplement: S11 Fig — N = 41, 40, 45, and 43 for CD4 T cells, CD8 T cells, monocytes and neutrophils respectively. (PDF) [file pgen.1005908.s011.pdf]

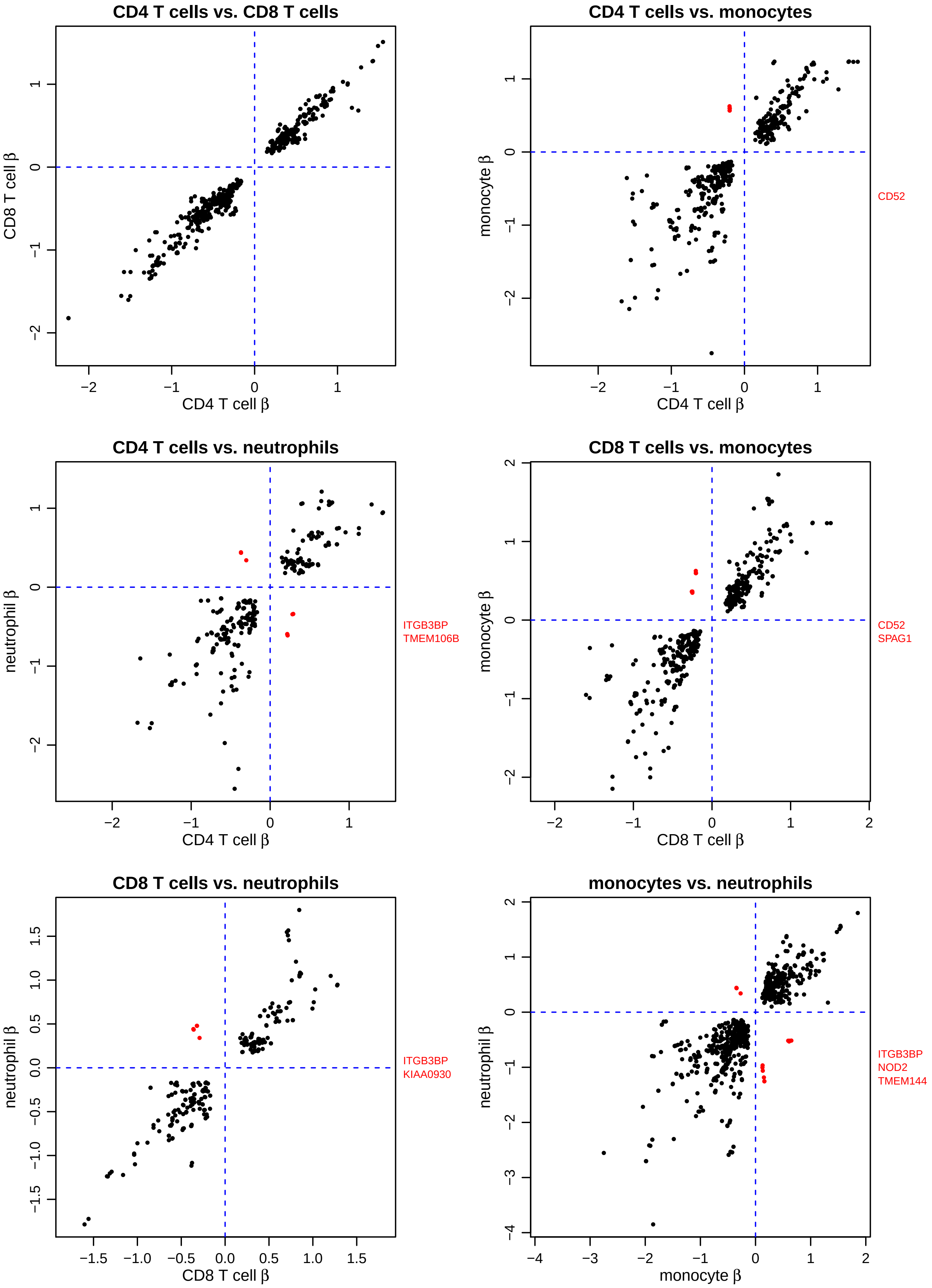
CD4 T cells vs. CD8 T cells
CD8 T cell β
CD4 T cell β
CD4 T cells vs. monocytes
monocyte β
CD4 T cell β
CD52
CD4 T cells vs. neutrophils
neutrophil β
CD4 T cell β
ITGB3BP
TMEM106B
CD8 T cells vs. monocytes
monocyte β
CD8 T cell β
CD52
SPAG1
CD8 T cells vs. neutrophils
neutrophil β
CD8 T cell β
ITGB3BP
KIAA0930
monocytes vs. neutrophils
neutrophil β
monocyte β
ITGB3BP
NOD2
TMEM144
