# Supplementary material for: Insight into Genotype-Phenotype Associations through eQTL Mapping in Multiple Cell Types in Health and Immune-Mediated Disease
Source: PLoS Genet. 2016 Mar 25;12(3):e1005908. doi: 10.1371/journal.pgen.1005908 (PMC4807835; doi:10.1371/journal.pgen.1005908)
Supplement: S1 Text — (PDF) [file pgen.1005908.s026.pdf]

Supplementary Information

Insight into genotype-phenotype associations through eQTL mapping in multiple cell types in health and immune-mediated disease

James E. Peters, Paul A. Lyons, James C. Lee, Arianne C. Richard,
Mary D. Fortune, Paul J. Newcombe, Sylvia Richardson, Kenneth G.C. Smith

Supplementary Methods

Permutation method for estimating FDRs

Here we describe the application of the 'plug-in' method[1] for estimating the FDR associated with a given significance threshold to eQTL analysis. Having run the eQTL association scan using the observed data, we empirically estimate the distribution of the test statistics under the null hypothesis that there is no association between genotype and expression using a permutation approach. The expression matrix has p rows and n columns, where each row represents expression values for one probeset and each column expression values for one sample (one individual). The columns of the expression matrix are randomly permuted so that the expression values assigned to each individual are randomly swapped, but the correlation structure between transcripts is unchanged. The genotype matrix is not altered. The eQTL association scan is run again using the permuted expression data, generating a set of test statistics. The permutation procedure is repeated multiple times, and for each permutation we repeat the eQTL scan, generating another set of test statistics. The empirical distribution of the statistics thus generated can be used to approximate the null distribution as any genotype-expression relationship should have been broken through the permutation procedure.

For each value of the test statistics produced by running the eQTL scan with the real data, we set the cut-off C for significance to that value. We can then estimate the FDR for that value of C as follows.

1. We count the number of test statistics (chi-squared scores) greater than or equal to this threshold in the real data to give us the number of results called significant, R.

2. We count how many test statistics in the permuted data (i.e. under the null hypothesis) were greater than or equal to threshold C. Dividing this number by the number of permutations, gives an estimate of the number of false positives, $\hat{V}$.

3. Our estimated FDR using that C as a significance threshold is then $\hat{V}/R$.

4. We repeatedly perform this procedure, each time changing the value of the cut point C to the next score in the real data. We eventually have FDR estimates for setting C to the value of each score in the real data.

Thus for each SNP-gene association we have a chi-squared score, and a corresponding estimated FDR were we to use that score as the threshold for significance.

eQTLBMA versus comparison of lists

As described in the Results, the application of a strict significance cut-off to declare the presence of eQTLs in each cell type, followed by intersection of the resulting lists of significant hits, as used in previous studies[2], might lead to erroneous claims of cell-type specificity. We therefore used the Bayesian joint modelling method, eQTLBMA[3], to investigate eQTL sharing between cell types. In Fig 1, we presented results using all available samples in order to maximise power (data from 91 IBD patients and 43 HVs in the IBD-HV analysis, and from 46 patients in the AAV analysis). eQTLBMA can be run if even if some individuals lack expression data for certain cell types using the option `--error hybrid` (see Methods). For estimates of cell-type specificity from one-at-a-time cell-type analysis, we limited analysis to the 65 individuals (IBD-HV) with expression data available for all cell types, to ensure equal power for each cell type. In order to present a direct and fair comparison between eQTLBMA and single tissue analysis, we here present the results from running eQTLBMA

on expression data from only these 65 individuals. As the expression data for each cell type came from the same set of individuals, we used option `--error mvlr`. Unsurprisingly, fewer eQTLs were detected when confined analysis to the 65 individuals, but the patterns in the results were very similar compared to those seen when using all samples, with 45% of eQTLs shared across all 5 cell types, and 8% declared unique to one (S19 Fig).

Does eQTLBMA unduly favour finding that eQTLs are shared?

To investigate whether the Bayesian model unduly favoured declaring eQTLs as shared across all cell types, we randomly permuted the sample labels for the CD4 T cell expression data, leaving the genotype data and the expression data for the other cell types unchanged. We then re-ran the analysis with eQTLBMA. Permutation of the CD4 T cell expression data should break up any genotype-expression relationship in this cell type. Therefore if the eQTLBMA method is behaving appropriately we would expect very few eQTLs to be found in CD4 T cells. Indeed, we found that after permutation of the CD4 T cell expression data, only 10 genes were declared as having an eQTL in CD4 T cells (0.5% of the number of genes declared to have an eQTL in any cell type). The Jaccard coefficient between CD4 and CD8 T cells was 0.1% (S6 Fig), compared to 100% in the real (unpermuted) data. These findings indicate that eQTLBMA is not inappropriately favouring the model of eQTL sharing between cell types.

How confident is the 'best' model?

As described in the Methods, our approach to deciding the best model using eQTLBMA was as follows. We took genes found to have a significant eQTL (5% Bayes FDR) in at least one cell type. For these genes, we identified the SNP with highest posterior probability of being the eQTL. For these SNP-gene pairs, we then examined the 'best' model of presence/absence across the cell types i.e. the configuration with the highest posterior probability. From this we were able to calculate the numbers of eQTLs for which the best model was presence in 1, 2, 3, 4, or all 5 cell types. However, it is possible that two or more competing models could have very similar PPs. In such a situation, we are less confident in the 'best' model. To investigate this further, we used the Shannon entropy to measure uncertainty. The Shannon entropy is defined as $H(X) = -\sum_i P(x_i) log_b P(x_i)$, where b is the base of the logarithm used. A high Shannon entropy indicates a high degree of uncertainty. Where the posterior probability for one configuration is much higher than all others, the Shannon entropy will be low. In contrast, where there are competing models with similar probabilities the Shannon index will be high. The distribution of Shannon entropies across SNP-probeset pairs is shown in S20 Fig, grouped according to the best configuration. This shows that where the best configuration was presence of the eQTL across all 5 cell types, the Shannon entropies were more skewed towards zero than for other configurations. This indicates that confidence in the model selection was highest for those eQTLs declared common to all cell types. Therefore the high proportion of eQTL sharing we observed from using eQTLBMA cannot be ascribed to the the 'common to all cell types' configuration narrowly 'beating' alternative models with similar posterior probabilities.

Notes on the use of a linear model with a genotype $\times$ disease interaction term to find IBD-dependent eQTLs

In the Methods section, we described how use of a linear model with a genotype$\times$disease interaction term was superior to the naive approach of separate eQTL analyses in IBD patients and in healthy volunteers (HVs), followed by comparison of the resulting lists of eQTLs detected in each cohort. Where the genotype$\times$disease interaction term is significant, we have good evidence that the effect

of genotype on expression differs in IBD versus health. In contrast, the naive approach of comparison of lists could lead to an eQTL that is truly present in both IBD and health being falsely declared IBD-specific simply because the test statistic for the association just passed the significance threshold in IBD and narrowly failed to reach significance in health. The short-comings of the comparison of lists approach are compounded because the IBD and HV cohorts were of different sizes, and so the statistical power to detect eQTLs was not the same in each.

More formally, the two approaches are testing different null hypotheses.

Consider first the naive approach of separate analyses of the IBD and HV cohorts, followed by the comparison of lists of eQTLs discovered in each. Simple linear models of the form shown in Equation (1) are fitted for the HV and IBD datasets separately. For clarity of presentation we first consider the case of testing association of expression of one gene with genotype at one SNP.

$$Y = \alpha + \beta X + \epsilon \tag{1}$$

where Y is the vector of gene expression levels for individuals 1 to n; X is the vector of genotypes for individuals 1 to n (which can take values 0, 1, or 2); α and β are constants, and ϵ is the error term. $\hat{y}_i$, the predicted value of Y for the i th individual is given by:

$$\hat{y}_i = \alpha + \beta X_i \tag{2}$$

The error term or residual for the i th individual, ϵ_i, is the observed value (y_i) minus the predicted value $(\hat{y}_i)$. The error term, ϵ, is assumed to be normally distributed with mean zero.

In Equations (3) and (4) we fit this form of linear model separately to each dataset (IBD and healthy volunteers). Subscripts $_H$ and $_{IBD}$ indicate in health and in IBD respectively. For clarity, at this stage we are still considering only one SNP-gene pair.

$$Y_i = \alpha_H + \beta_H X_i + \epsilon_{1i} \qquad \text{for healthy individuals } i = 1, ..., n_1 \tag{3}$$

$$Y_j = \alpha_{IBD} + \beta_{IBD} X_j + \epsilon_{2j} \qquad \text{for IBD patients } j = 1, ..., n_2 \tag{4}$$

In Equation (3) we can test the null hypothesis that β_H equals zero i.e. in healthy people there is no effect of genotype on expression (no eQTL). Similarly in Equation (4) we can test the null that β_{IBD} equals zero (there is no eQTL in IBD patients).

Now consider testing expression of the gene against all *cis* SNPs, and repeating this process for all genes in the expression matrix. These models are simply fitted again for each SNP-gene pair. At the end of this process, we have a list of SNP-gene pairs where we have found sufficient evidence to reject the null hypothesis that β_H equals zero, and a list where we have rejected the null that β_{IBD} equals zero. These lists can be intersected to find eQTLs detected in only one condition or the other.

Using a significance threshold corresponding to a false discovery rate of 5% means that on average 5% of the eQTLs we declare as significant in each group are false positives. In addition there will be type 2 errors i.e. eQTLs that are truly present that we do not identify. Thus false positives in each list (and false negatives not in the lists) may cause eQTLs to falsely be declared condition-specific.

In order to take a more robust approach to finding eQTLs influenced by the presence or absence of disease, we instead jointly analysed the IBD and healthy volunteer data together using the following linear model with a genotype×disease interaction term:

$$Y_i = \alpha + \beta_1 X_i + \beta_2 D_i + \beta_3 X_i D_i + \epsilon_i \qquad \text{for individuals } i = 1, ..., n_1 + n_2 \tag{5}$$

where
Y is expression level
X is genotype
D is disease status (0= healthy, 1=IBD)
$D_i = 0$ for individuals $i = 1, ..., n_1$
$D_i = 1$ for individuals $i = n_1 + 1, ..., n_2$
and α and the βs are constants.

Equation (5) is a multivariate regression model as we have more than one predictor variable. We can test the following null hypotheses:

1. α equals zero (i.e. the intercept is zero), which is generally not of biological interest.
2. β_1 equals zero (i.e. there is no main effect of genotype on expression).
3. β_2 equals zero (i.e. there is no main effect of IBD on expression). Rejecting this null is equivalent to stating that the gene is differentially expressed in IBD versus health.
4. β_3 equals zero i.e. there is no genotype×disease interaction effect.

SNP-gene pairs where the genotype×disease interaction term is significant indicate that the effect of genotype on expression is significantly different in IBD versus health. In biological terms, this includes (i) eQTLs present in health but abrogated in IBD, (ii) eQTLs present in IBD but not in health, (iii) eQTLs with opposing directions of effect in health compared to IBD, and (iv) eQTLs whose effects in health and IBD are in the same direction, but of significantly different magnitudes (S12 Fig). More formally, the interaction term assesses whether there is a significant difference in the slope of the genotype-expression regression line between healthy individuals and IBD patients i.e. whether the effect size of a unit change in allele dose on expression is significantly different between health and IBD.

Consider individuals $i = 1, ..., n$. They are healthy (i.e. $D_i = 0$). Therefore

$$Y_i = \alpha + \beta_1 X_i + \epsilon_i$$

If there is no genotype×disease interaction, β_3 equals zero. Therefore for individuals $i = n_1+1, ..., n_2$ who have IBD (i.e. $D_i = 1$),

$$\begin{aligned} Y_i &= \alpha + \beta_1 X_i + \beta_2 + \epsilon_i \\ &= (\alpha + \beta_2) + \beta_1 X_i + \epsilon_i \end{aligned}$$

$\alpha + \beta_2$ is simply a constant (the intercept). Therefore if there is no genotype×disease interaction, the slope of the regression line is β_1 in both IBD patients and healthy individuals. Thus we can think of testing the null hypothesis that there is no genotype×disease interaction ($\beta_3 = 0$) as equivalent

to testing whether $\beta_H = \beta_{IBD}$ in Equations (3) and (4). The only difference is that in Equation (5) the error term is assumed to come from the same distribution for both HVs and IBD patients. By testing whether β_H and β_{IBD} from Equations (3) and (4) are equal, we have to first estimate the error separately for IBD patients and HVs resulting in lost of power. Note that the naive approach of asking which eQTLs are significant in HVs and which are significant in IBD patients is not the same thing. The latter compares the list of eQTLs for which we have significant evidence to conclude $\beta_H \neq 0$ and that $\beta_{IBD} \neq 0$, which is problematic for the reasons outlined previously.

eQTLs with contrasting direction of effect on gene expression between cell types

Comparisons between eQTL direction of effect in CD8 T cells and monocytes were shown in Fig 3. Here we show the results for all cell type pairings. We show results from the joint analysis of the IBD-HV cohort, the analysis of the IBD cohort alone, and the analysis of the AAV cohort.

Joint IBD-HV analysis

eQTLs with opposite directions of effect after *in vitro* inflammatory stimuli have been described[4]. Given this, we considered the possibility that some eQTLs might have the opposite direction of effect in IBD versus health, and that this could confound our comparison of direction of effect between cell types in the joint IBD-HV analysis if the sample composition for each cell type differed. It is unlikely that eQTLs with opposing directions of effect between IBD and health would be declared significant in the joint analysis of the IBD-HV cohort, as the opposing signals in each cohort would be expected to mask one another. Nevertheless, to err on the side of caution, when comparing directions of effect between eQTLs in different cell types using the joint IBD-HV dataset we restricted analysis to the same set of individuals to avoid the potential for any such confounding. There were 65 individuals in the IBD-HV dataset with expression data available for all five cell types, and 93 individuals with expression data available in CD4 and CD8 T cells, monocytes and neutrophils. Therefore to increase power we performed an analysis omitting B cells using the 93 samples (S8 Fig; analysis using expression data after adjustment with PEER[5]). We emphasise that we only plotted the effect sizes where the SNP-gene association was significant in *both* cell types to reduce the possibility that eQTLs that appeared to have discordant effects were false positives. S9 Fig shows results limited to the 65 individuals. Clearly, fewer eQTLs are detected (either in total or with discordant effects) in the latter analysis.

Separate analysis of IBD cohort

We repeated the comparisons of directions of eQTL effect between cell types using results from eQTL mapping restricted to patients with IBD. When analysing the IBD cohort on its own, concerns about eQTLs having opposite effects in IBD versus health no longer apply. Therefore, to maximise power, we performed eQTL mapping in each cell type using all IBD available samples. This meant that, although highly overlapping, the sets of individuals used for analysis in each cell type were not identical. (S10 Fig). Because, unlike in the IBD-HV analysis we were not restricted to the subset of individuals with full expression data, we had more power and found more eQTLs with discordant effects between cell types.

AAV cohort

We are reassured that the majority of eQTLs with discordant effects between cell types are not false positives from analysis using the independent AAV dataset (S11 Fig). Unsurprisingly we found

many fewer eQTLs (either in total or with discordant effects) in the AAV analysis, given the AAV sample size was around half that of the IBD-HV data. However, of the genes with statistically significant eQTLs that had discordant effects between cell types in the AAV data, all but one of these genes (*SPAG1*) were identified as having a discordant effect in the IBD-HV analysis (S11 Fig). For example, the finding of eQTLs with discordant effects on *CD52* expression in CD8 T cells versus monocytes in the IBD-HV data, was replicated in the independent AAV analysis. Moreover, where a discordant eQTL was found in the IBD-HV data, but the eQTL did not pass significance in both cell types in the AAV dataset when controlling the FDR at <5%, the trend of discordant effects could nevertheless frequently be seen. An example is the *CD101* eQTL, which was significant in the IBD-HV data in both monocytes and CD8 T cells, but with opposing directions of effect between the cell types (Fig 3). In AAV, the eQTL was also significant in monocytes with direction of effect consistent with the IBD-HV data. In CD8 T cells, the eQTL was not significant in AAV, but the direction of effect was consistent with that in the IBD-HV data.

Intersecting eQTLs with disease-associated SNPs identified through GWAS

We took IBD-associated SNPs from Supplementary Table 2 of the IBD meta-analysis by Jostins *et al*[6], and proxy SNPs in high linkage disequilibrium ($r^2 > 0.8$), and intersected these with eQTL SNPs (eSNPs) from our analysis (Figs S13-S14, and Table S4). The IBD meta-analysis paper[6] defines SNPs which are associated specifically with Crohn's disease ('CD-associated') or with ulcerative colitis ('UC-associated'), and those which are associated with both ('IBD-associated'). We use the same nomenclature here. We compared the results of our eQTL analysis with those from eQTL database mining in the IBD meta-analysis paper[6], which synthesised eQTL information from three different sources: The University of Chicago eQTL database (http://eqtl.uchicago.edu/cgi-bin/gbrowse/eqtl), The Dixon *et al* eQTL dataset (http://www.sph.umich.edu/csg/liang/asthma/), and the Merck Research Laboratories eQTL dataset[7]. These databases contain results from eQTL studies in LCLs, fibroblasts, T cells, monocytes, liver, omental adipose tissue, and subcutaneous adipose tissue. The SNP annotation in Table S4 (e.g. intronic, intergenic) was performed with SNPnexus[8] (http://snp-nexus.org/index.html).

We also intersected all IBD-associated SNPs (CD, UC, or both) listed in the NHGRI GWAS catalogue (and proxy SNPs in high linkage disequilibrium) with eSNPs from our analysis. The genes whose expression is associated with these eSNPs are shown in S15 Fig, according to which cell type the eQTL is found in. Using this information, we may now update our list of 'IBD-associated genes' based not on proximity to disease risk SNPs but on the effects of these SNPs on gene expression. The list of IBD-associated SNPs in the NHGRI catalogue is not confined to those listed in the IBD meta-analysis[6] as it includes the lead SNPs reported by all previous IBD genome-wide association studies.

Figs S13-S15 were generated by intersecting *all* significant (FDR <0.05) eQTL SNPs with GWAS SNPs and their proxies. We also implemented a more conservative approach to declaring overlap of eQTLs and GWAS hits, described as follows. Where there were multiple significant eSNPs for a given gene (as typically occurs due to linkage disequilibrium), we took only the most-highly associated eSNP per gene (the 'peak' eQTL signal). The list of peak eQTL SNPs thus generated was then intersected with the list of GWAS SNPs and their proxies. S16 Fig has been generated using this approach, and as a result contains fewer genes than S15 Fig.

Figs S13-16 have all been generated based on the eQTL mapping performed using PEER-adjusted[5] expression data and all available IBD and HV samples (i.e. not restricted to the individuals for whom genotype and expression data was available in all cell types). As a result, the sample size, and hence

power to detect eQTLs, vary between cell types (see Table 1 for sample sizes for each cell type).

References

[1] Hastie T, Tibshirani R, Friedman J, editors. The elements of statistical learning. 2nd ed. Springer; 2001.

[2] Fairfax BP, Makino S, Radhakrishnan J, Plant K, Leslie S, Dilthey A, et al. Genetics of gene expression in primary immune cells identifies cell type-specific master regulators and roles of HLA alleles. Nat Genet. 2012 May;44(5):502–510.

[3] Flutre T, Wen X, Pritchard J, Stephens M. A statistical framework for joint eQTL analysis in multiple tissues. PLoS Genet. 2013 May;9(5):e1003486.

[4] Fairfax BP, Humburg P, Makino S, Naranbhai V, Wong D, Lau E, et al. Innate immune activity conditions the effect of regulatory variants upon monocyte gene expression. Science. 2014 Mar;343(6175):1246949.

[5] Stegle O, Parts L, Piipari M, Winn J, Durbin R. Using probabilistic estimation of expression residuals (PEER) to obtain increased power and interpretability of gene expression analyses. Nat Protoc. 2012 Mar;7(3):500–507.

[6] Jostins L, Ripke S, Weersma RK, Duerr RH, McGovern DP, Hui KY, et al. Host-microbe interactions have shaped the genetic architecture of inflammatory bowel disease. Nature. 2012 Nov;491(7422):119–124.

[7] Greenawalt DM, Dobrin R, Chudin E, Hatoum IJ, Suver C, Beaulaurier J, et al. A survey of the genetics of stomach, liver, and adipose gene expression from a morbidly obese cohort. Genome Res. 2011 Jul;21(7):1008–1016.

[8] Dayem Ullah AZ, Lemoine NR, Chelala C. A practical guide for the functional annotation of genetic variations using SNPnexus. Brief Bioinformatics. 2013 Jul;14(4):437–447.
